# An account on using bio-renewable solvents in organic synthesis: advances, prospects, and challenges

**DOI:** 10.1039/d6ra01629d

**Published:** 2026-07-22

**Authors:** Yaqoob A. Teli, Sangita Kalita, S. Aleena Chanu, Saikat Dutta, Chandi C. Malakar

**Affiliations:** a Department of Chemistry National Institute of Technology Manipur Imphal-795004 India chdeepm@gmail.com cmalakar@nitmanipur.ac.in; b Department of Chemistry, National Institute of Technology Karnataka (NITK) Surathkal Mangalore-575025 Karnataka India sdutta@nitk.edu.in

## Abstract

The use of solvents during organic transformations is ubiquitous in synthetic organic chemistry and chemical manufacturing due to clear advantages. Biomass-derived, renewable, and innocuous organic solvents have garnered significant interest in recent years as economically feasible and environmentally sustainable alternatives to petroleum-derived solvents. This review provides an account of the recent advances in using various bio-renewable organic solvents during organic transformations and their functional efficacy compared to conventional solvents. Synthetic routes for producing the bio-renewable solvents from biomass-based feedstock have been deliberated. The bio-renewable solvents have been classified based on the functional groups in their molecular structure, and their physicochemical properties have been elaborated. Various classes of organic transformations, including acid- and base-catalysed reactions, metal-catalysed coupling reactions, and redox reactions, have been discussed with mechanistic insights. This literature review will assist the researchers in appreciating the major achievements, identifying the challenges, and expanding the scope of employing bio-renewable solvents in organic synthesis.

## Abstract 2

Biorenewable solvents are gaining prominence in organic synthesis for their environmental benefits. This work classifies them by functional groups, summarizes key physicochemical properties, and highlights their roles as reaction media and beyond.

## Graphical abstract

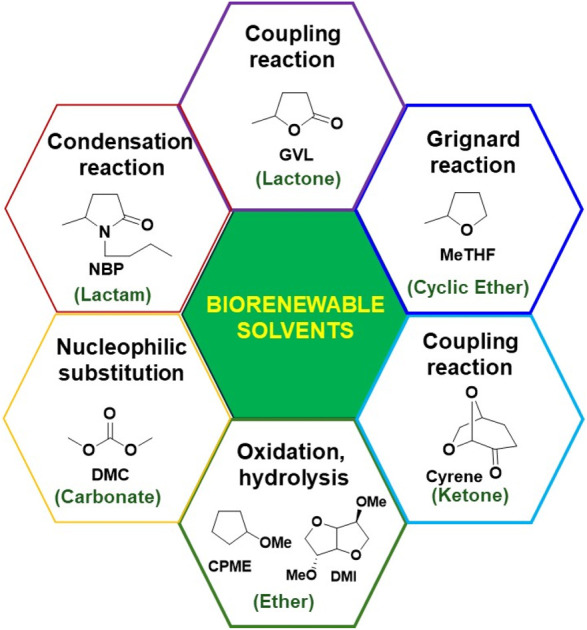

## 1. Introduction

Organic solvents are commonly used during the manufacturing or formulation of pharmaceuticals, agrochemicals, fragrances, cosmetics, paints, and polymers, among others. The current use of organic solvents in the paints industry alone is around 46%; 9% in the pharmaceutical industry; 6% in cosmetics; 6% in household care; 4% in polymer manufacturing; 4% in industrial cleaning; 2% in agrochemicals; and 11% in others.^1^ Solvents are used as reaction media or during the purification processes, such as chromatography, extraction and recrystallisation. Many of the organic solvents encountered daily pose serious concerns regarding their human health and ecological hazards, toxicity, environmental persistence, and poor sustainability.^2,3^ The chemical industries release a huge amount of value-added chemicals, which are highly reliant on fossil fuels. Upon processing crude oil, bulk chemicals are produced using conventional organic solvents for the fine chemical and petrochemical industries. The depletion of crude oil and increasing sustainable and environmental concerns have drawn attention to the identification and application of renewable feedstocks and alternative resources, for which the selection of an appropriate solvent is highly important. The rapid increase in biomass conversion is the preferred solution, reducing reliance on fossil-based products and serving as an alternative solvent.^4^ Most commonly, the organic compounds used as solvents include aliphatic and aromatic hydrocarbons, esters, ketones, ethers, and halogenated compounds. The limitations of these solvents include toxicity, explosivity, flammability, and volatility. The National Institute for Occupational Safety and Health (NIOSH) has categorised some of these solvents as neurotoxic (*e.g.*, toluene, *n*-hexane, and tetrachloroethylene), carcinogenic (*e.g.*, carbon tetrachloride, benzene, and trichloroethylene), and teratogenic (*e.g.*, methyl chloride, 2-methoxyethanol).^5^ Major waste streams generated in the organic chemicals manufacturing (including pharmaceuticals) industries are associated with solvent use.^6^ It is not surprising that scientific communities and industrialists are more concerned about solvent use.^7^ Thus, limiting the use of traditional solvents, which are derived almost entirely from fossil sources, and implementing biomass-derived solvents represents a major advancement in the current era of research in the chemical industry, in good agreement with the twelve principles of green chemistry.^8^

**Scheme 1:**
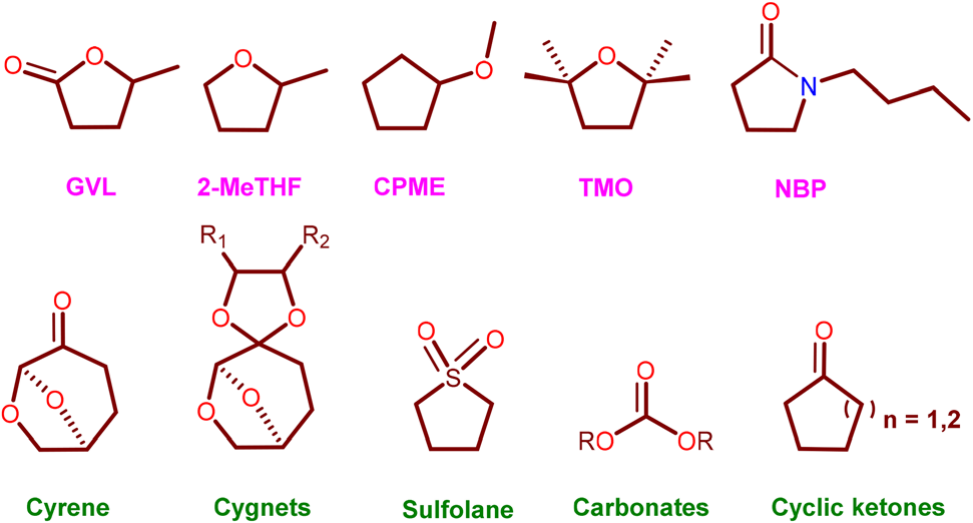
Molecular structure of some selected bio-renewable solvents.

Biomass-derived solvents are considered to be more viable than traditional solvents. These solvents are produced mainly from renewable biomass, including food waste, crops, and forestry residues. The main characteristics of these solvents are that they are less toxic and hazardous than traditional solvents and are biodegradable, which makes them easy to dispose of. In addition to this, many other solvents are also placed in this class, including water, ionic liquids, gas-expanded liquids, liquid polymers, supercritical fluids and biomass-derived solvents. So, by implementing biomass-derived solvents in chemical reactions and other areas of research, replacing a traditional solvent can dramatically influence reaction outcomes.^9^ The most commonly used biomass-based solvents are listed in (Scheme 1).

## 2. Physicochemical properties of biomass-derived solvents and traditional solvents

The physicochemical properties of the biomass-derived solvents cover Hansen Solubility Parameters (HSP), GlaxoSmithKline (GSK), and Kamlet–Taft (KT) parameters, which were employed as a reference guide to differentiate the solvent of interest. Based on a GlaxoSmithKline (GSK) solvent selection guide, factors such as boiling point, environmental impacts, recycling, reactivity/stability, and health hazards (Table 1) are given due importance in evaluating the sustainability of existing and new solvents.^7^ The revised GlaxoSmithKline (GSK) solvent selection guide consists of 110 solvents^10^ among which a few are mentioned in Table 2. The guidance of these solvents is particularly dependent on the user's requirements.

**Table 1:** Physicochemical properties of some biomass-derived solvents

Solvents	B.P. (°C)	Recycling	Environmental impact		Health hazards	Reactivity/stability
			Aquatic	Air		
γ-Valerolactone (GVL)	207	7	7	4	4	10
2-Methyltetrahydrofuran (2-MeTHF)	78	5	10	10	4	6
Propylene carbonate	242	5	9	6	10	10
Cyrene	203	4	8	6	4	10
DMSO	189	4	10	5	7	5
DCM	39.6	8	3	2	2	9
Cyclopentanone	131	9	6	6	7	10
Cyclohexanone	156	7	5	5	6	9
TMO	102	6	6	6	4	5
Eucalyptol	176	6	9	6	5	8
Dimethyl isosorbide (DMI)	236	4	4	3	4	8
Cyclopentyl methyl ether	106	4	7	5	4	9
Methyl *tert*-butyl ether	75	8	10	8	7	9
Acetonitrile	82	5			7	10
Propylene carbonate	242	5	10	6	10	10
Acetone	56	6	10	6	10	9
*N*,*N*-Dimethylacetamide (DMAc)	165	6	10	4	1	9
*N*,*N*-Dimethylformamide (DMF)	153	6	10	6	1	9
*N*-Methyl-2-pyrrolidone (NMP)	202	4	5	7	1	9
*N*-Butylpyrrolidinone (NBP)	146	6	8	4	5	8
1,4-Dioxane	102	1	8	7	4	6
Diethyl ether	35	7	9	7	1	6
Dimethyl carbonate (DMC)	91	3	9	6	10	10
Cygnet	180	5	9	6	6	8

**Table 2:** Hansen solubility parameters (HSP) and Kamlet–Taft parameters (KT) of selected bio-renewable solvents

Solvent	Hansen solubility parameters (HSP)			Kamlet–Taft parameters (KT)		
	*δ*_D_	*δ*_P_	*δ*_H_	π*	*β*	*α*
γ-Valerolactone (GVL)	15.5	4.7	6.6	0.83	0.60	0.00
2-MeTHF	16.9	4.0	5.3	0.53	0.58	0.00
Propylene carbonate	20	18	4.1	0.90	0.38	0.00
Cyrene	20.3	18.2	10.9	0.90	0.39	0.00
DMSO	18.8	10.6	6.9	0.00	0.76	1.00
DCM	18.2	6.3	6.1	0.13	0.10	0.13
Cyclopentanone	18.4	16.4	10.2	1.00	0.76	0.00
Cyclohexanone	17.9	11.9	5.2	0.76	0.52	0.00
TMO	17.8	8.4	5.1	0.68	0.53	0.00
Eucalyptol	15.4	2.4	2.1	0.35	0.77	0.00
DMI	16.7	4.6	3.4	0.36	0.61	0.00
Cyclopentyl methyl ether	17.6	7.1	7.5	0.84	0.43	0.00
Methyl *tert*-butyl ether	16.7	4.3	4.3	0.42	0.53	0.00
Acetonitrile	14.8	4.3	5.0	0.25	0.45	0.19
Acetone	15.5	10.4	7.0	0.71	0.48	0.08
DMAc	16.8	11.5	10.2	0.88	0.76	0.00
DMF	17.4	13.7	11.3	0.88	0.79	0.00
NMP	18.0	12.0	7.0	0.92	0.77	0.00
NBP	17.5	9.9	5.8	0.77	0.92	0.00
1,4-Dioxane	17.5	1.8	9.0	0.55	0.37	0.00
Diethyl ether	14.5	2.9	5.1	0.27	0.47	0.00
DMC	15.5	8.6	9.7	0.47	0.38	0.00
Cygnet	18.3	7.3	7.4	1.09	0.17	0.00

Table 1 covers the boiling points of the solvents, their environmental impacts on air and water, recycling, health hazards, including acute and chronic effects on human health, and the reactivity/stability of the solvents. The Hansen solubility parameters and Kamlet–Taft parameters listed in Table 2 have also been included as a reference guide for selecting a solvent. The solvents that display similar Hansen space in a three-dimensional sphere exhibit similar solvency.^11^ The Hansen solubility parameters were based on the method described by Charles M. Hansen, following the principle of “like dissolves like”, which provides a quantitative assessment of the dispersed and continuous phases. The miscibility of these solvents was described using the specific contributions from non-polar dispersion energy (*δ*_D_), hydrogen-bonding energy (including Lewis acid–base interactions) (*δ*_H_), and polar-dipolar energy (*δ*_P_), measured in units of per molar volume MPa^1/2^. Solvents in proximity in a three-dimensional Hansen space exhibit similar solvency. For example, Pacheco *et al.*^12^ identified NMP, DCM, and other aprotic and chlorinated solvents used in polymer processing can be replaced with solvents near 3 MPa^1/2^, such as levoglucosenone.^13^ The Kamlet–Taft parameters underscore the effects of solvent change on chemical reactions through a Gibbs free energy relation, using the parameters of acidity (*α*), basicity (*β*), and solvent polarizability/polarity (π*).^14^ The basicity (*β*) of polar aprotic solvents can be measured by the highest energy molecular orbital and the most electronegative atom of the solvent molecule. For example, NBP shows the highest bond basicity (0.92), whereas Cygnet shows the lowest (0.17). In contrast, polarity/polarizability depends on the quadrupolar amplitude, the dipole moment, and the highest-energy molecular orbital, and it diminishes as the solvents' polarizability increases. Solvents listed in Table 2 do not exhibit hydrogen bond acidity (*α*) except acetone (0.06) and acetonitrile (0.19).

## 3. Different types of solvents

### 3.1. γ-Valerolactone (GVL)

**Scheme 2:**
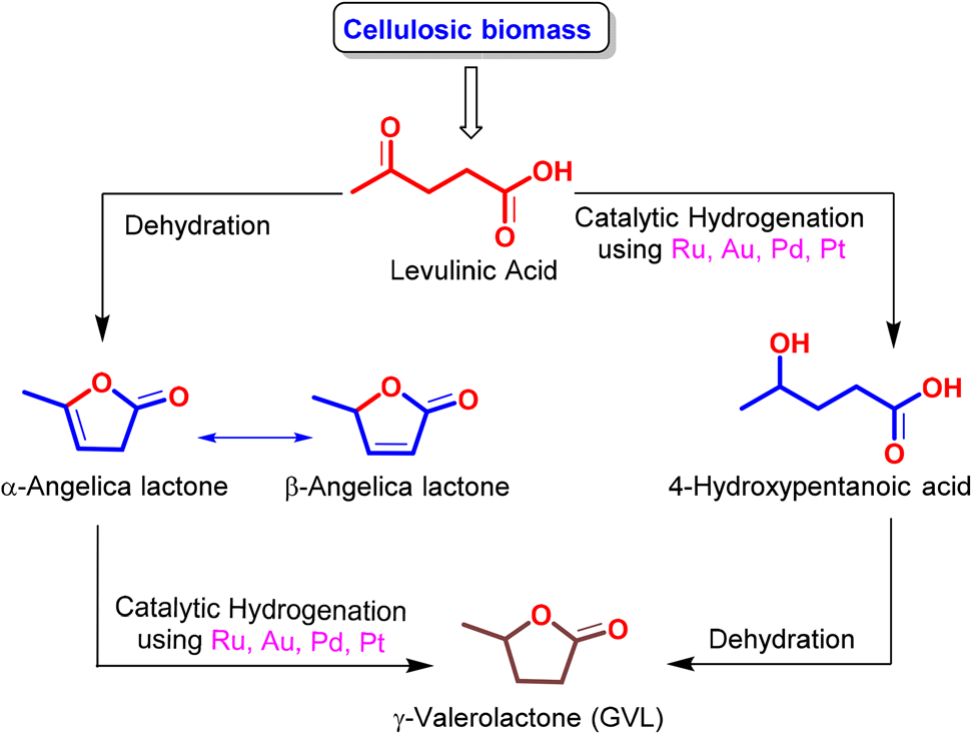
Transformation of LA into GVL.

γ-Valerolactone (GVL, b.p. 205 °C) is a dipolar aprotic solvent obtained from biomass-derived carbohydrates *via* LA as a platform chemical (Scheme 2).^15,16^ Numerous publications have discussed the synthesis of GVL by reducing LA under homogeneous and heterogeneous catalysis.^16–18^ Homogeneous catalysts often face challenges, such as high cost, poor catalyst recycling, and complicated product purification. Heterogeneous catalysts are preferred due to their convenient recovery, superior recyclability, and straightforward product purification.^19*b*^ GVL is a popular bio-renewable solvent because of its Kamlet–Taft and HSP parameters that are comparable to those of conventional solvents such as acetone, NMP, DMAc, and DMF.^19*a*^ Some of the major organic transformations where GVL is used as a solvent includes palladium-catalysed cross-coupling reactions,^20^ lignin depolymerisation,^21^ C–H functionalization chemistry,^22^ solid-phase peptide synthesis,^23^ and electrochemistry.^20*b*,24^ GVL is biodegradable, less toxic to humans, and less ecotoxic than traditional solvents.

### 3.2. Carbonates and cyclic carbonates

**Scheme 3:**
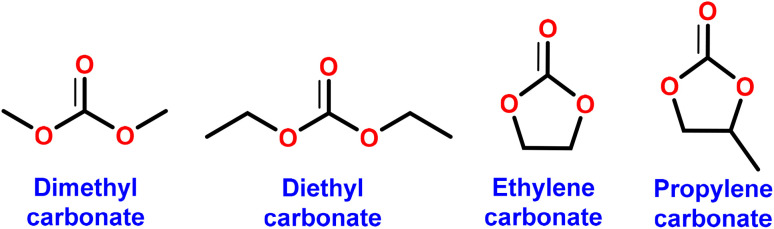
Some commonly employed carbonate-based solvents.

Cyclic carbonates have applications in cosmetics, oil processing, surfactant formulations, and as a green solvent in organic reactions. Carbonate-based solvents include cyclic carbonates, such as propylene carbonate, ethylene carbonate and acyclic carbonates, whereas acyclic carbonates include DMC and diethyl carbonate. Cyclic carbonates are favourable solvents based on the GlaxoSmithKline (GSK) solvent selection guide.^25^ Some of the commonly employed carbonate-based solvents are listed in (Scheme 3).

**Scheme 4:**
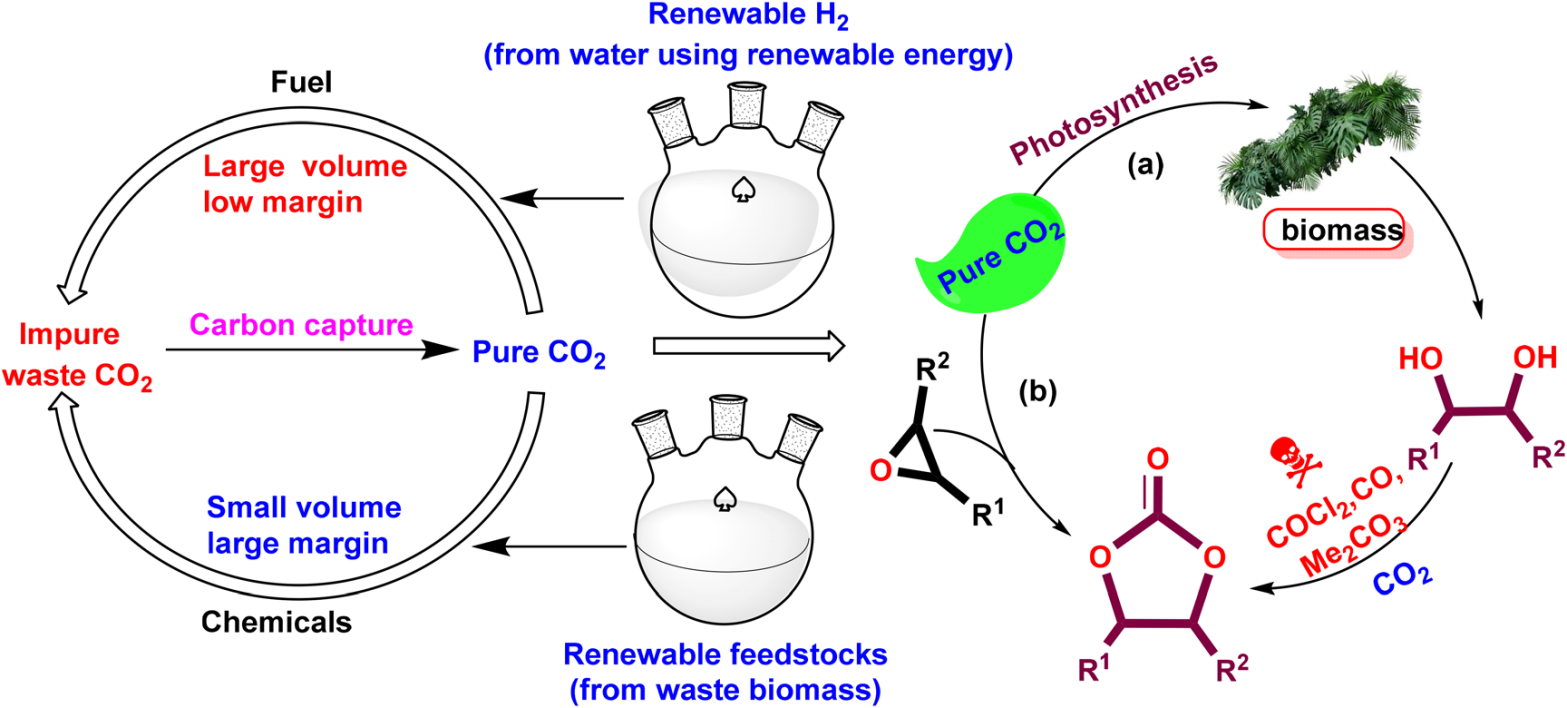
Carbon dioxide sourcing and sequestration for synthesising cyclic carbonates.

Carbonate solvents are well-suited due to their excellent features, such as easy accessibility, low cost, moderate boiling points, biodegradability, and low toxicity. As depicted in (Scheme 4), biomass-derived CO_2_ can be used as feedstock for bio-renewable chemicals and biofuels. While the biofuels market is enormous, the value addition is limited. On the other hand, the market for bio-renewable chemicals is relatively small but offers significant value addition. The synchronous use of CO_2_ for synthesising chemicals (*e.g.*, organic carbonates) and fuels (*e.g.*, methane) can effectively utilise up to 25% of CO_2_ produced annually.^26,27^

The cyclic carbonates are also produced from hydroxyl compounds, which are easily accessible. Cyclic carbonates are also produced from readily accessible hydroxyl compounds. These inexpensive and readily available compounds are obtained mainly from natural product separation, organic synthesis and from biomass conversion. These substrates are ideal for producing carbonates using various carbonating agents. From the previous literature, we conclude that carbon monoxide,^28^ phosgene,^29^ urea,^30^ DMC,^31^ and carbon dioxide (CO_2_)^32^ have been exploited with vicinal diols to synthesise cyclic carbonates. Due to limitations such as causticity, toxicity, and potential for poisoning, the use of most carbonating agents is limited. However, carbon dioxide produced from biomass can be used to produce carbonates by direct condensation with vicinal diols, leaving water as the sole product.^33,34^ On the other hand, cyclic carbonates are prepared from the reaction between ethylene oxide or propylene oxide and carbon dioxide^35^ with a 100% atom economy. This process is being carried out by different homogeneous and heterogeneous catalysts under ambient reaction conditions.

### 3.3. Cyrene

**Scheme 5:**
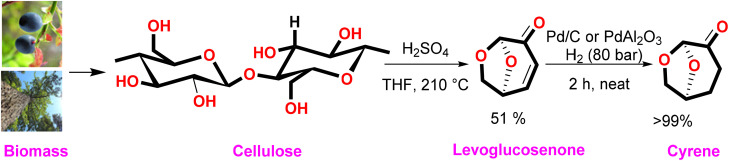
Synthesis of Cyrene from cellulose as a biomass precursor.

Modern synthetic chemistry is searching for less toxic, less hazardous solvents that may be derived from biomass and could be a potential alternative to REACH-restricted solvents. Consequently, the chemical industry will be sustainable and more eco-friendly by using biomass-derived feedstocks as the potential solvent. This has inspired the scientific community to find an alternative to the REACH-restricted DMF and NMP solvents, which are dipolar aprotic in nature.^36^ Cyrene (dihydrolevoglucosenone) has been proposed as the potential biomass-derived dipolar aprotic solvent for this purpose.^37^ Initially, the cellulose in biomass is dehydrated into LGO under acid-catalysed pyrolysis, followed by hydrogenation of the olefin group in LGO (Scheme 5).^38–42^ It has been found that cellulose is the highest-yielding LGO feedstock. Broido *et al.* in 1973 reported the pyrolytic synthesis of LGO from cellulose.^43^ Subsequently, Kudo *et al.* devised an efficient Pd/C-catalysed hydrogenation of LGO to Cyrene.^44^ The preparation technique and yield of LGO have been improved over the past decade.^45–47^ The preparation technique and yield of LGO have been improved over the past decade. Cyrene has a wide temperature range for use as a solvent, with a boiling point of 227 °C and a melting point of −27 °C. In addition, Cyrene is biodegradable, has very low acute oral toxicity, and is not mutagenic.^48^ The calculated Kamlet–Taft and HSP parameters indicate that Cyrene occupies the Hansen solvent space near NMP.^49^

Over the past few years, Cyrene has been employed as a potential green solvent in diverse synthetic transformations, including MOF synthesis,^50^ Pd-catalysed cross-coupling reactions,^51^ and amide synthesis,^52,53^ among others. It is further interesting to note that Cyrene's ketonic form remains in dynamic equilibrium with the corresponding diol form, and the maximum solubility of solid reactants is found to be dependent on the diol form. As a consequence, Cyrene can be used as a biomass-derived green solvent with tuneable properties.^54^ The drawback of Cyrene is that inorganic bases do react with it, catalysing aldol reactions. Furthermore, ocular irritation can occur upon contact with this solvent.^48^

### 3.4. Cygnet solvent

**Scheme 6:**
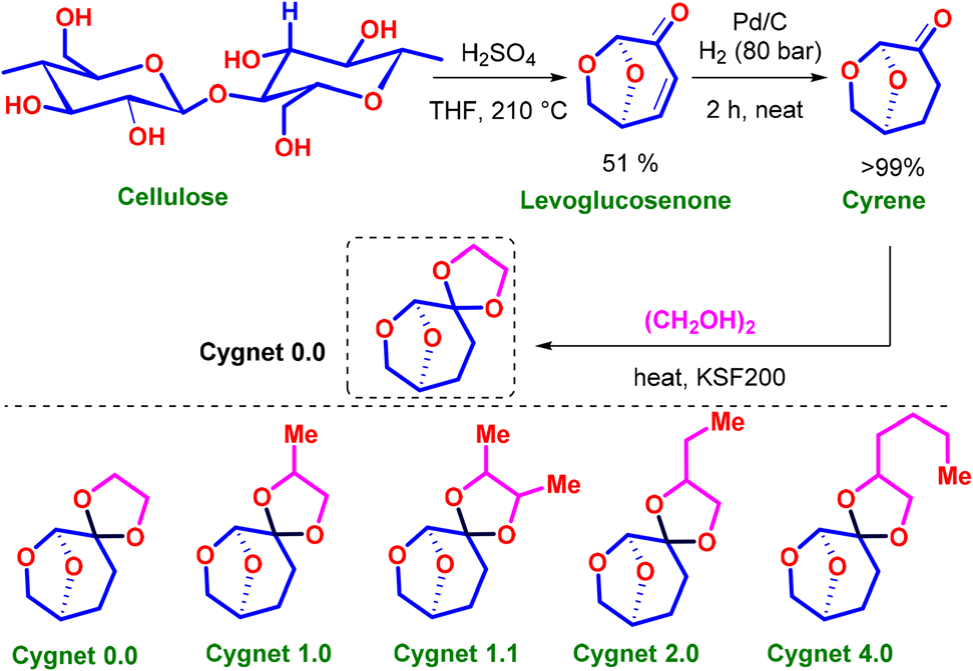
Synthesis of Cygnet solvents from biomass and the molecular structure of some common Cygnets.

Considering the limitations of Cyrene solvent in alkaline conditions, a suitable modification was required. It was achieved by protecting the ketal counterpart of the Cyrene solvent with ethylene glycol or its functionalised derivatives to form the corresponding ketals, called Cygnet.^1^ To date, five variants of the Cygnet solvent have been reported. Cygnet is produced by condensing Cyrene and ethylene glycol in the presence of an acid-clay catalyst, with water as a byproduct (Scheme 6).^55^ Cygnet 0.0 is the simplest, prepared by condensation of Cyrene and ethylene glycol.^56^ The melting points of Cygnet solvents are significantly higher than those of Cyrene. For example, Cygnet 0.0 has a melting point of 70 °C, making it unsuitable for low-temperature reactions.^57^ In this respect, it is suitable to mention that the nomenclature of the variants of the Cygnet solvent does depend on the number of carbon atoms attached to each carbon of the ethylene glycol unit. Cygnet 1.1 represents the presence of one carbon atom and one methyl group in each of the carbon atoms of the ethylene glycol unit. The Hansen solubility parameters of the Cygnet solvent have been found to match closely with the Hansen solubility space of DCM. Cygnet 0.0 has been assumed to be a green solvent with no toxicity and/or mutagenicity reported to date. Cygnet 0.0 has been assumed to be a green solvent with no toxicity and/or mutagenicity reported to date. Cygnet 0.0 has been placed as a biomass-derived green solvent in the NIPs membrane casting process.^56^ Additionally, in combination with Cyrene, Cygnet 0.0 has been utilised as a green solvent in the biocatalytic synthesis of polyester. Moreover, Cygnet 0.0 has been used as a green solvent in Heck coupling reactions and fluorination reactions. It is interesting to note that Cygnet 0.0 is miscible with water, which makes its separation and purification from the aqueous reaction medium challenging.^58^

### 3.5. *N*-Butylpyrrolidinone (NBP)

**Scheme 7:**
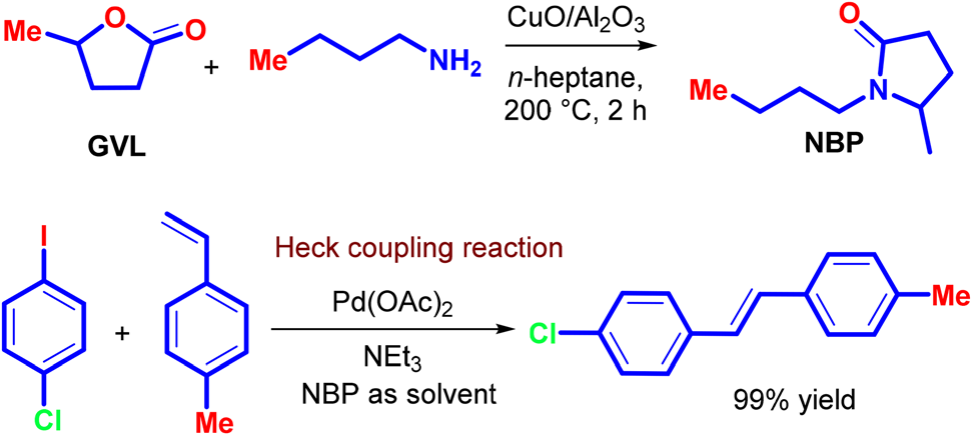
Sustainable synthesis of NBP and its application as a solvent in Heck coupling.

NBP is known as a reproductive toxin and was declared a substance of great concern by the EU and the Environmental Regulation Committee. Naturally, there was a need for an alternative dipolar, aprotic solvent which could replace NMP. Starting from NEP, eventually it was reported that NBP could be the best possible alternative to the NMP solvent, having most of the characteristics of a dipolar aprotic solvent. Based on experimental findings, it was concluded that NBP is not reprotoxic, non-mutagenic, and, more importantly, biodegradable.^59^ Although NBP can be prepared from renewable feedstocks, it has limitations like acute oral toxicity and a very high boiling point of 241 °C (Scheme 7). So, this solvent should be used with all the necessary precautions as a green solvent and alternative to toxic dipolar, aprotic solvents like DMF, NMP, and DMAc. NBP has been employed as an alternative to DMF in solid-phase peptide synthesis.^60^ Comparable yields in Heck cross-coupling reactions for the synthesis of diverse heterocycles have been achieved using NBP as the solvent rather than dipolar aprotic solvents.^59^ In addition, numerous nucleophilic substitution reactions have been performed in NBP to demonstrate its viability in synthetic organic chemistry as a competitive alternative to conventional dipolar aprotic solvents. Recently, Zaccheria *et al.* (2023) developed a novel protocol using a copper-catalyst system to synthesise NBP from GVL.^61^

### 3.6. 2-Methyltetrahydrofuran (2-MeTHF)

**Scheme 8:**
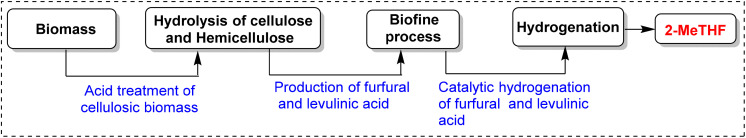
Summary of the overall process to convert biomass into 2-MeTHF.

**Scheme 9:**
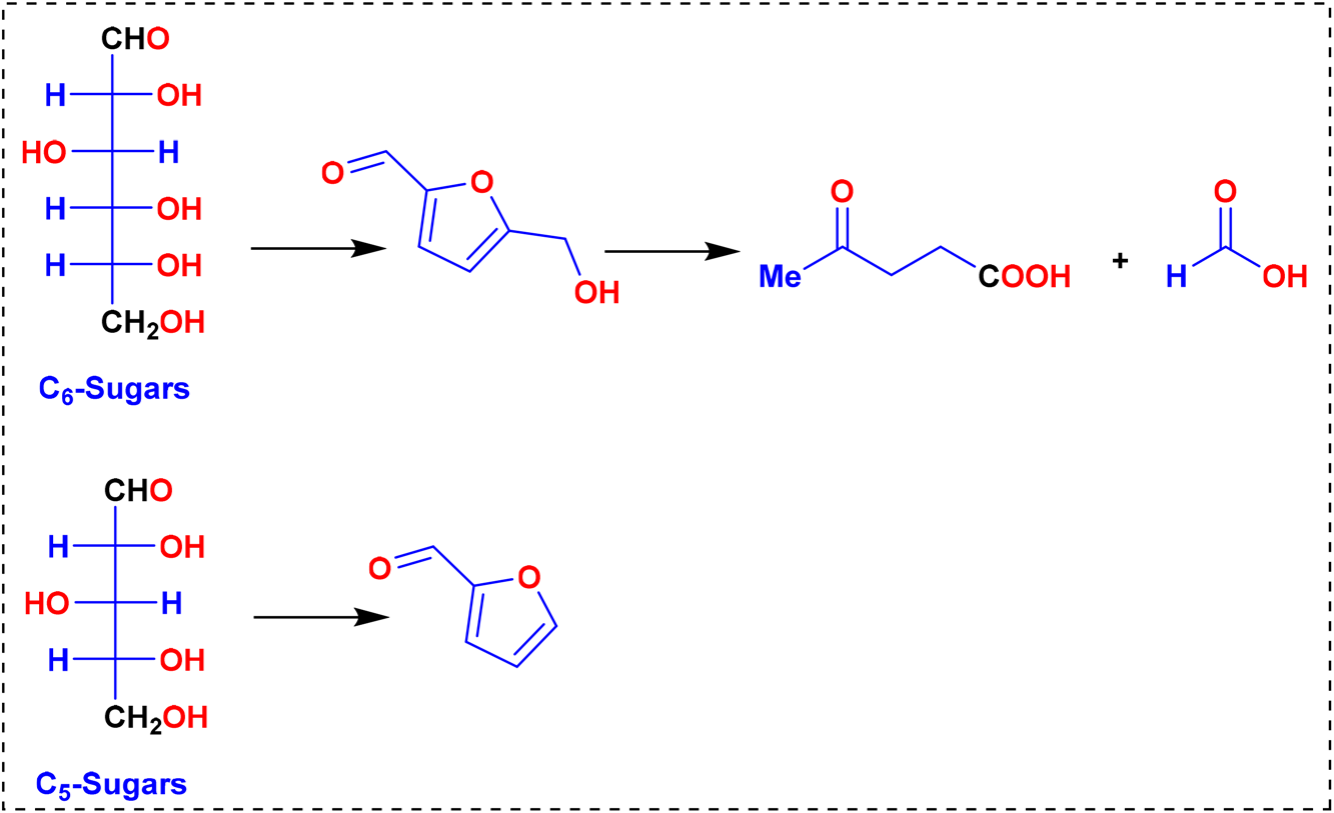
Chemical transformations in the Biofine process.

**Scheme 10:**
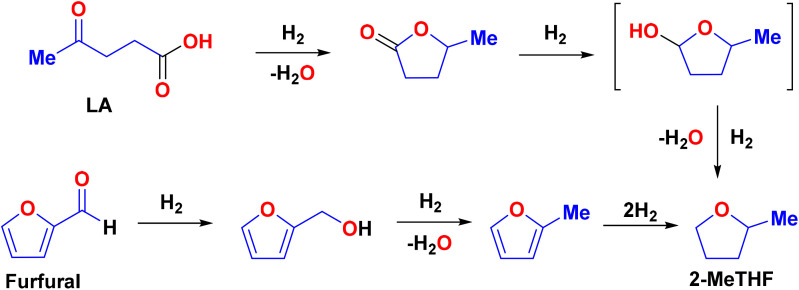
Hydrogenation of furfural and LA into 2-MeTHF.

2-MeTHF, a cyclic ether-based solvent, can be synthesised from LA or furfural,^62^ which is derived from agricultural waste like rice straw, corn stover and sugar cane bagasse.^63^ The conversion of biomass to 2-MeTHF requires few number of consecutive steps as shown in (Scheme 8), which includes acidic treatment of biomass (lignocellulosic material) by sulphuric acid to produce C_5_ and C_6_ sugars, in the next step these C_5_ and C_6_ sugars are used to produce LA or furfural. The major drawback of the synthesis is the separation of C_5_ sugars from C_6_ sugars, so the bio-fine process is the only industrial method to produce either LA or furfural from a mixture of C_5_ and C_6_ sugars, with up to 50 wt% yield of LA from cellulosic biomass on a dry mass basis (Scheme 9). The final step involves the catalytic hydrogenation of LA or furfural, yielding 2-MeTHF in good selectivity (Scheme 10).

Compared to a conventional chemical synthesis of tetrahydrofuran (THF),^64^ the life cycle analysis of 2-MeTHF has shown that manufacturing of the solvent from waste corn cob furfural can cut emissions by up to 97%. It is appealing for use in organometallic,^65^ organocatalytic,^66^ and biotransformation^67^ syntheses as well as the processing of lignocellulosic materials^68^ due to its favourable physical and chemical characteristics, including low miscibility with water,^69^ boiling point,^70^ remarkable stability compared to other cyclic-based solvents and others.^71^ It has been marketed as a “drop-in” alternative to THF, offering a more environmentally friendly and long-lasting solution, as 2-MeTHF occupies a solvent space that is complementary to THF. Many reagent solutions, including Grignard and lithium reagents,^72^ are now commercially available in 2-MeTHF, further encouraging their use as workable substitutes for THF and diethyl ether.^62^ 2-MeTHF has already been used as a sustainable solvent in several organic transformations, often yielding satisfactory results.^73–75^ Favourable toxicological evaluations of 2-MeTHF promise to expand its usage in multistep organic synthesis, process chemistry, and also as a biofuel.^76^ Even though 2-MeTHF is bio-renewable and biodegradable, more research is warranted on its techno-economic and life-cycle analysis.^77^

### 3.7. Cyclopentyl methyl ether (CPME)

**Scheme 11:**
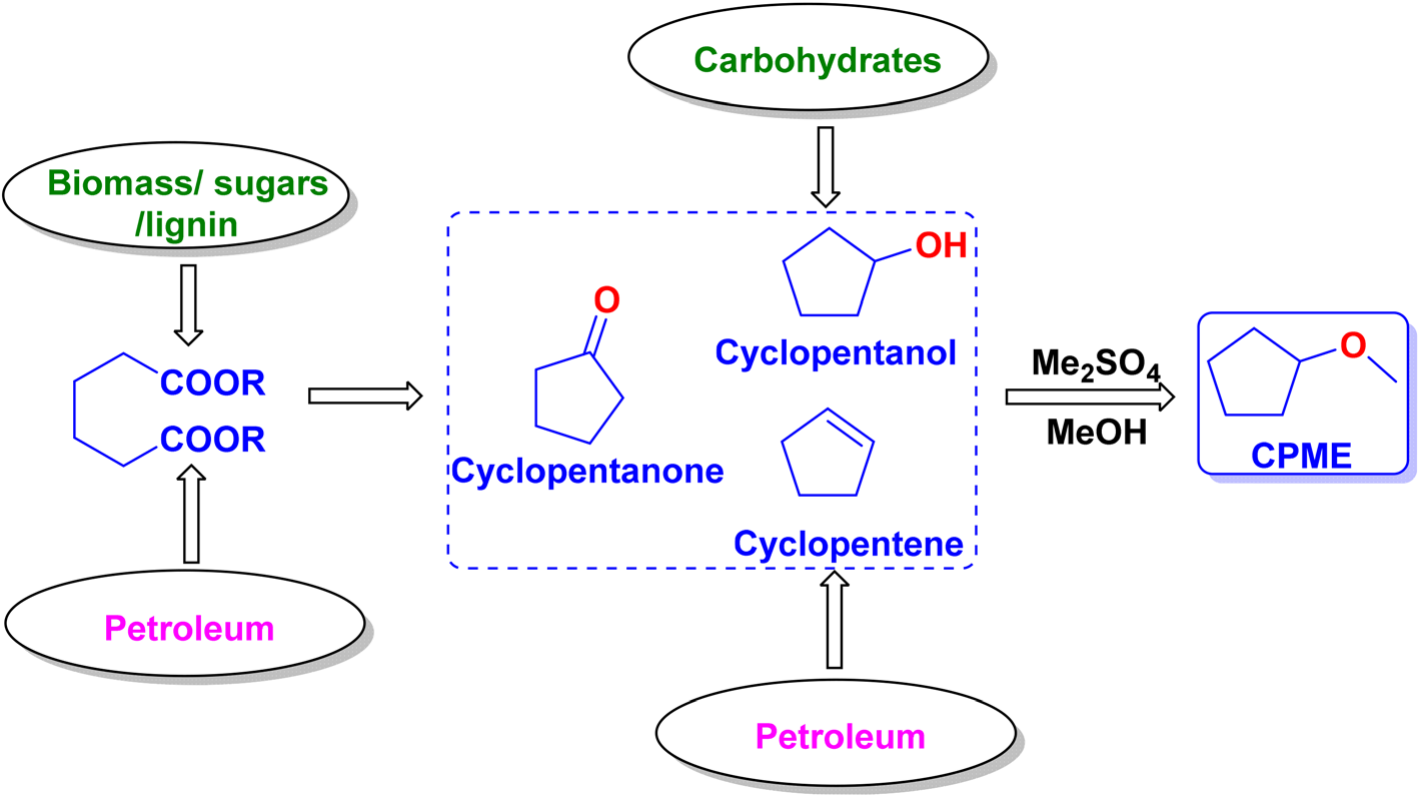
Production of CPME from cyclopentanol and cyclopentene.

Cyclopentyl methyl ether (CPME), a hydrophobic ethereal solvent, has a reasonably high boiling point of 106 °C.^78^ Since 2007, CPME has been increasingly used in synthetic organic chemistry as a solvent and a substitute for conventional ether-based solvents, such as THF and 1,4-dioxane.^79^ In 2019, it was placed in a more sustainable and environmentally benign class of solvents.^79*b*^ Several synthetic routes are available for the renewable production of CPME from its chemical precursors, like cyclopentanol and cyclopentene. Both precursors can be produced by the hydrogenation of furfural to furfuryl alcohol, followed by Piancatelli rearrangement under hydrothermal conditions. Finally, cyclopentanol or cyclopentene is converted to its methyl ether by reaction with methanol or DMC (Scheme 11).^80,81^ The petroleum route of producing cyclopentanone involves the decarboxylative ketonisation of adipic acid.^80^ Interestingly, adipic acid can be sourced renewably from biomass *via* a chemical-catalytic or an enzymatic pathway.^80^ Given that 1,4-dioxane and THF are two other ethereal solvents with similar HSP values close to CPME, they possess very similar solubilising properties.^82^ As a bio-renewable solvent, CPME offers high chemical stability in acids and bases, oxidative stability, a wide operating temperature range, low toxicity, safer handling, low latent heat of vaporisation, and ease of recycling.^79^ CPME's toxicological evaluation by Watanabe *et al.* in 2013 revealed that it had a reasonably low acute toxicity and was neither a skin sensitiser nor a mutagen.^83^ CPME has been successfully used in a variety of organic reactions, including organometallic chemistry,^84^ acid catalysis,^85^ transition metal catalysis,^86^ amidation reactions,^87^ oxidations,^88^ radical reactions,^89^ and an organic phase in biphasic chemistry due to its low solubility in water (1.1 wt%).^90^ Finally, it is noteworthy that the production of CPME, which outperforms a prospective derivation from renewable sources, is based on non-renewable sources and uses a 100% atom-economy and highly effective synthesis. New developments in the conversion of furfural to cyclopentanol,^91^ a potential CPME precursor, foresee the realisation of an advantageous bio-based production of this solvent in the future.

### 3.8. 2,2,5,5-Tetramethyloxolane (TMO)

**Scheme 12:**
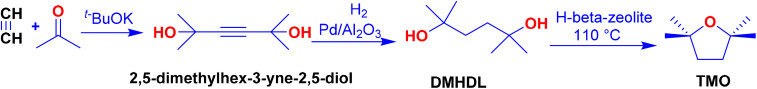
Synthetic route of TMO.

As part of the ReSolve initiative, 2,2,5,5-tetramethyloxolane (TMO), also known as 2,2,5,5-tetramethyltetrahydrofuran, is a cyclic ether and shows properties more aligned with toluene than the other traditional solvents. When exposed to UV light, heat, and air, epoxides do not form as they do with other conventional solvents, such as CPME, THF, and diethyl ether.^92^ Out of a variety of ethereal solvents, TMO has a favourable boiling point of 112 °C.^93^ Through a dehydration reaction, 2,5-dimethylhexyne-2,5-diol (which can also theoretically be made from renewable resources) can be converted into TMO (Scheme 12).^94^ In addition, a recent study by Byrne *et al.* evaluated several bio-based synthetic pathways to TMO from glucose, one of which utilised methyl levulinate (a byproduct of the Avantium process for synthesising polyethene furanoate).^94^ Solutes with hydrogen-bonding ability show a significant solubility difference between TMO and toluene; these solutes prefer the organic phase in the TMO/water system over the aqueous phase in the toluene/water system. This is because the TMO contains a lone pair on the ethereal oxygen atom, which leads to secondary interactions with the solutes, unlike in toluene. Hence, creates new opportunities for the use of this sustainable solvent in liquid–liquid extraction, especially in the separation of natural products.^95^ TMO has been studied by Pellis *et al.* as a potential substitute for toluene and THF in the biocatalysed synthesis of polyesters,^96^ whereas Yanrui *et al.* have evaluated its resin-swelling capacity.^97^ TMO works well as a solvent for the oxidation of 5-(hydroxymethyl)furfural to 2,5-diformylfuran, which is a more specialised application.^98^ Unlike conventional ethers, the absence of a hydrogen atom at the ethereal oxygen at the alpha location avoids the possibility of dangerous peroxides forming.^99^ Due to the four methyl groups covering the ethereal oxygen at the alpha-position, this unique structure also results in decreased basicity when compared to many typical ethers.^100^ As a result, this molecule exhibits solvent-like properties similar to those of typical hydrocarbon solvents, particularly toluene.^101^ Initial acid-stability experiments indicate that TMO may withstand acid attack at room temperature, and results under reflux are encouraging.^94^

## 4. Applications of bio-renewable solvents in organic synthesis

### 4.1. 2-Methyltetrahydrofuran (2-MeTHF)

Amide synthesis, a fundamental transformation in the pharmaceutical industry,^102,103^ is traditionally performed using coupling agents.^104–109^ But they are often hazardous, costly, and problematic for the environment.^110–117^ These reactions typically rely on polar aprotic solvents such as DCM and DMF, or toluene in certain cases, and all these solvents are facing increasing regulatory restrictions.^118–120^ Consequently, there is growing interest in catalytic methods and safer alternative solvents to enhance sustainability, reduce toxicity, and improve overall reaction performance.

**Scheme 13:**
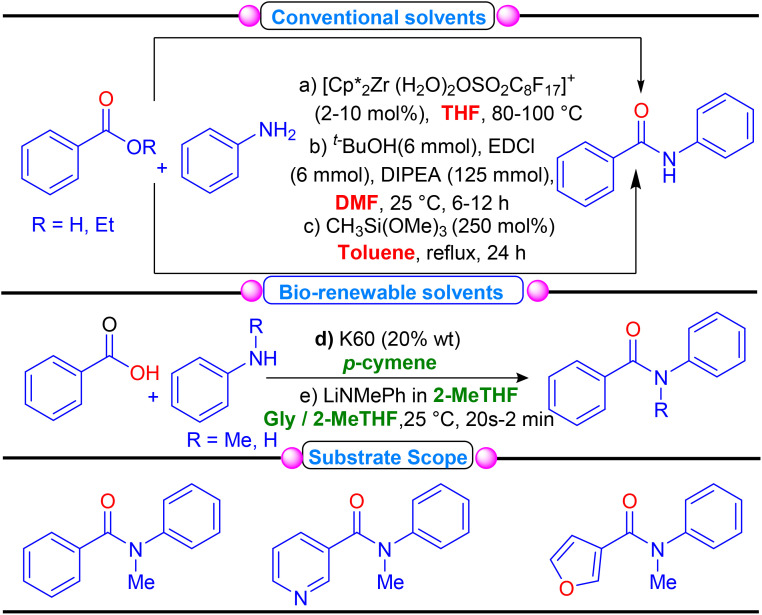
Strategies for the synthesis of amides.

The amide bond is a common structural motif found in many pharmaceutical compounds and natural products.^121,122^ Liang and colleagues (2018) reported a zirconium-based Lewis acid-catalysed amidation of carboxylic acids with aromatic amines in THF (Scheme 13a).^123^ In the same year, Jizhen *et al.* described a related transformation in DMF (Scheme 13b).^124,126^ Recently, methyltrimethoxysilane has been employed as a catalyst in toluene for amide synthesis (Scheme 13c).^125^ These studies primarily focus on catalyst development, while the role of alternative and sustainable solvents remains comparatively underexplored. Later, Petchey group reported the use of 2-MeTHF as a cosolvent in organolithium-mediated ester amidation reactions (Scheme 13e).^127^ The efficiency of 2-MeTHF is attributed to its ability to solubilise the lithium reagent and promote the formation of reactive aggregates. However, it should be noted that the reaction conditions and mechanism differ significantly from conventional amidation protocols.

**Scheme 14:**
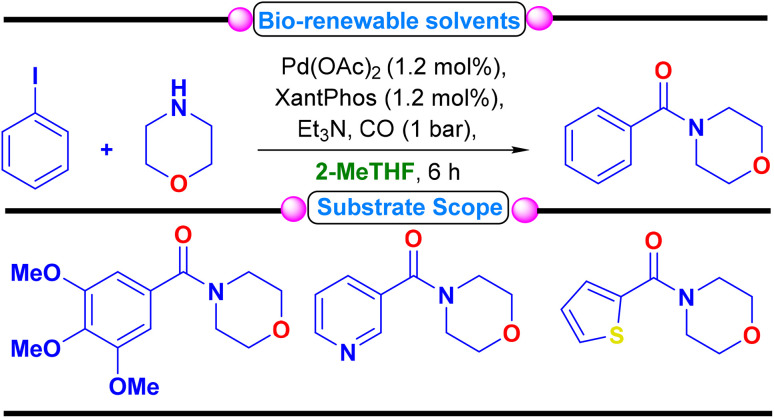
Palladium-catalysed amino-carbonylation.

The stabilizing effect of 2-MeTHF was also exploited by Takacs *et al.* in 2023 during the process optimisation of amide synthesis (Scheme 14).^128^ This palladium-catalysed amino-carbonylation process involves bio-renewable solvents, such as 2-MeTHF, methyl levulinate and ethyl levulinate. The optimization was carried out by employing iodobenzene, morphine, and CO as standard reactants under various conditions, including temperature, pressure, and ligands.

**Scheme 15:**
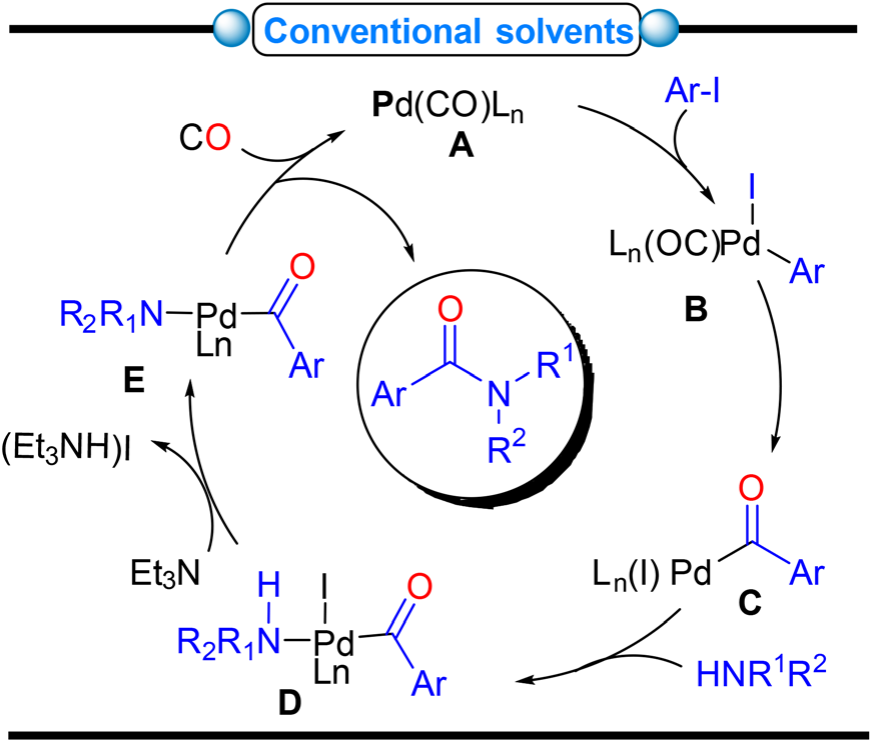
Mechanism for the synthesis of amides.

The proposed reaction mechanism involves some basic steps that are similar to those in conventional solvents (Scheme 15). The first step proceeds with the oxidative addition reaction of aryl halide to palladium(0) complex (Pd(CO)L_*n*_), A to form the palladium(ii)–aryl intermediate B. Next, the palladium(ii) undergoes a migratory insertion with carbon monoxide to generate palladium(ii)–acyl complex C. Further, the nucleophile (NuH) attacks intermediate C which results in the formation of catalytic intermediate D. The amido-acyl–palladium(ii) species E are produced by the elimination of HI in the presence of the base (Et_3_N) from complex D. The final step involves reductive elimination leading to the desired product, where Pd(0) species recycle back in the catalytic cycle.

**Scheme 16:**
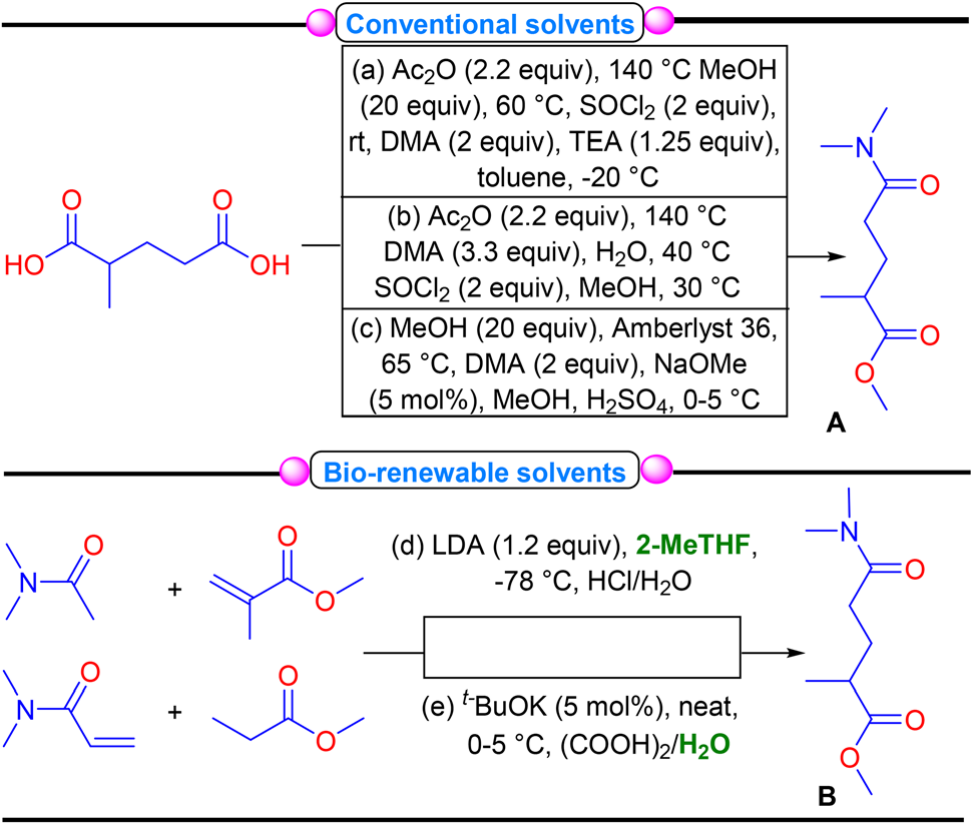
Synthesis of methyl 5-dimethylamino-2-methyl-5-oxopentanoate.

Methyl 5-(dimethylamino)-2-methyl-5-oxopentanoate, commercially available as Rhodiasolv PolarClean, has been proposed as a bio-based alternative to conventional polar aprotic solvents such as DMF, DMAc, and NMP.^129–131^ Three synthetic routes to PolarClean have been reported in the patented production process (Scheme 16a–c).^132^ Cseri *et al.* developed alternative synthetic strategies based on retrosynthetic analysis (Scheme 16d and e), enabling the preparation of high-purity methyl 5-(dimethylamino)-2-methyl-5-oxopentanoate *via* base-catalysed Michael additions using LDA and *t*-BuOK from readily available starting materials.^133^ The application of this solvent has been demonstrated in *N*- and *O*-arylation reactions proceeding *via* nucleophilic aromatic substitution (S_N_Ar) mechanism.

Application of this PolarClean's solvent was found in *N*- and *O*-arylation reactions proceeding through a nucleophilic aromatic (S_N_Ar) mechanism. This solvent shows magnificent stability even in raucous reaction conditions. This solvent provides an excellent yield of 77–91%, which is far better compared to traditional polar aprotic solvents are shown in Table 3.

**Table 3:** Screening of green polar aprotic solvents for the S_N_Ar reaction of benzimidazole with bis(4-fluorophenyl) sulfone

Entry	Solvent	Dielectric constant	Isolated yield (%)	HPLC purity (%)
1	B	28.3	91	98
2	PolarClean X	29.9	92	95
3	DMAc/toluene	37.8/2.4	70	N/A
4	PC	65.5	1	37
5	GVL	36.5	86	92
6	Cyrene	37.3	11	99

**Scheme 17:**
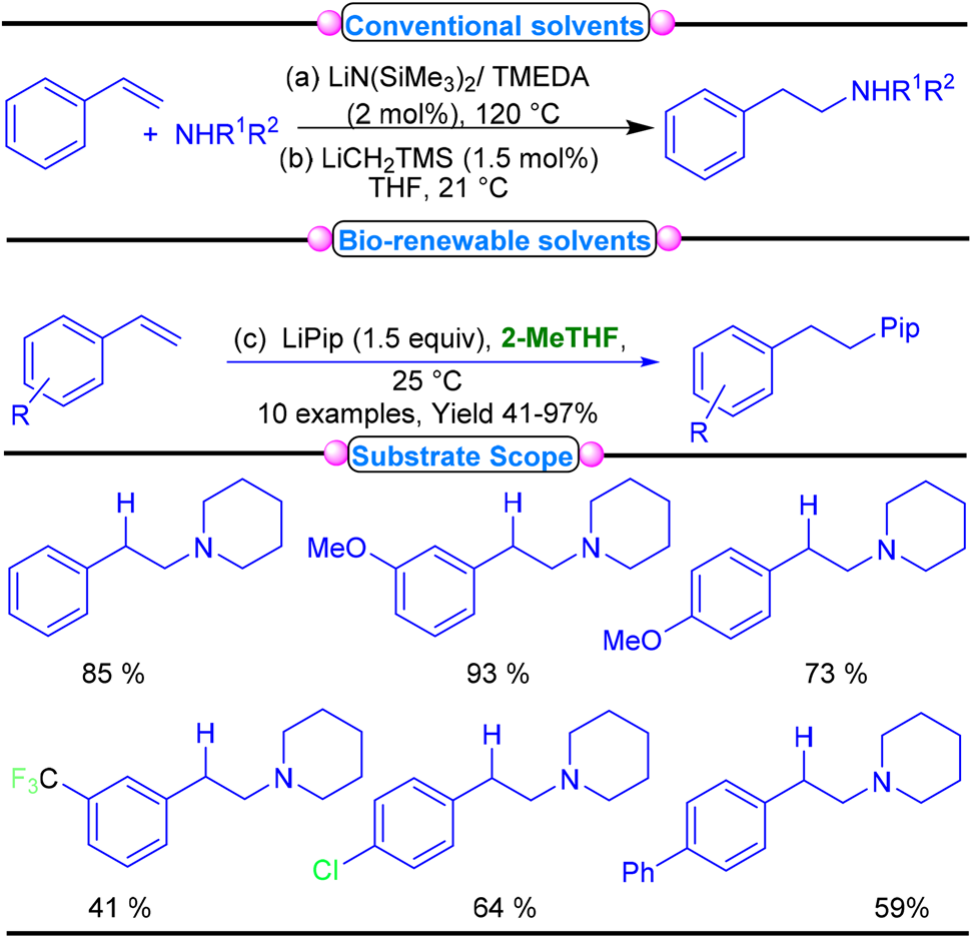
Hydroamination of vinyl arenes with amines by alkali metal amides.

Mulks *et al.*, investigated the hydroamination reactions for the synthesis of phenethylamine scaffolds, which are important intermediates in biologically relevant and synthetic applications.^141^ Despite their significance, such transformations remain challenging due to high activation barriers and relatively flat energy profiles.^134–137^ To address these limitations, several catalytic systems have been developed, including lithium-based systems in combination with TMEDA (Scheme 17a) or THF (Scheme 17b).^138–140^ In this context, the use of the bio-derived solvent 2-MeTHF was explored for the hydroamination of styrene (Scheme 17c).^141^ Under ambient temperature and pressure, the reaction proceeded efficiently in 2-MeTHF, affording the desired phenethylamine derivatives in excellent yields. While 2-MeTHF appears to be a suitable medium for this transformation, particularly under ambient conditions, the observed improvements in yield cannot be attributed solely to the solvent, as multiple reaction parameters differ between the reported conditions.

**Scheme 18:**
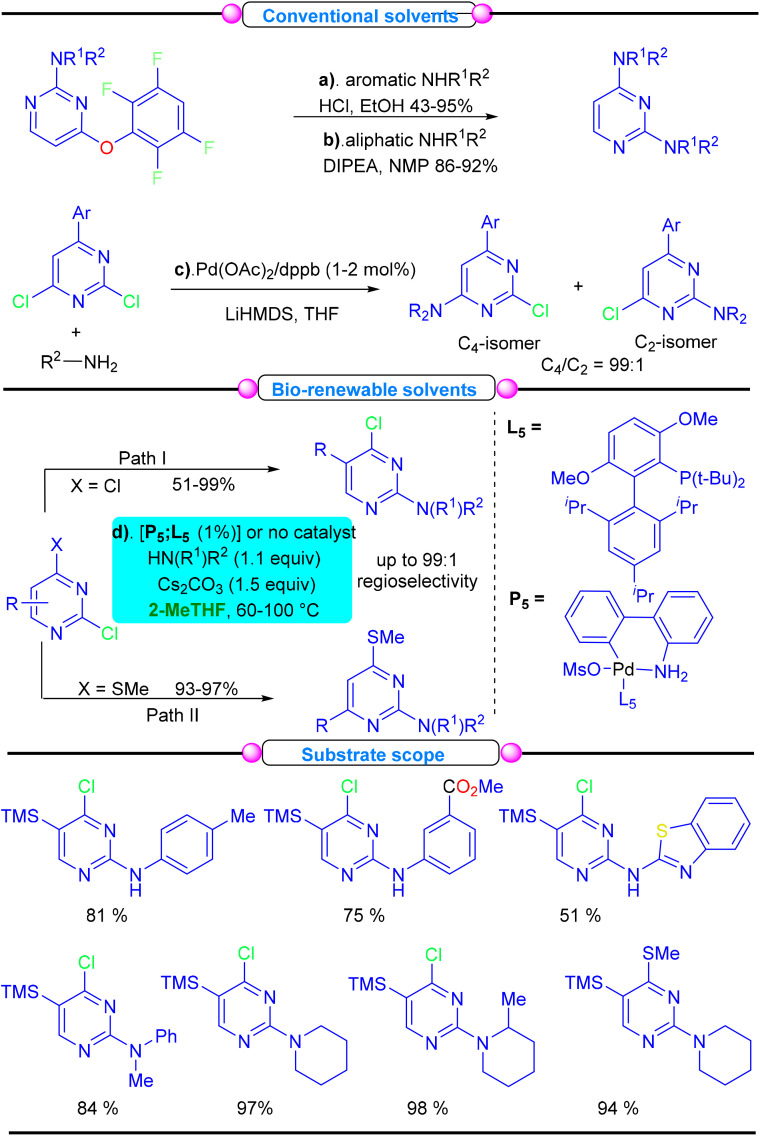
Synthesis of 2-aminopyrimidines.

C–N bond formation in pyrimidines, particularly 2,4-diaminopyrimidines, is important but challenging. Michael O'Donnell (2015) reported a regioselective multi-step synthesis using 2-chloro-4-tetrafluorophenoxypyrimidine and imines in EtOH, NMP, and THF solvents (Scheme 18).^142^ Earlier, Peng *et al.* (2006) developed a Pd-catalysed protocol in THF for 4-amino-2-chloropyrimidines (Scheme 18c).^143*a*^ Typically, aryl or heteroaryl amines require Pd catalysts, while dialkylamines react *via* S_N_Ar without a catalyst. Smith in 2016 introduced a regioselective domino process to synthesise 2-amino-4-chloropyrimidines from polychloropyrimidines and 4-chloro-6-methoxy-2-(methylthio)pyrimidine [path I (X = Cl), path II (X = SMe)] and R = OMe using 2-MeTHF (Scheme 18d).^143*b*^

**Scheme 19:**
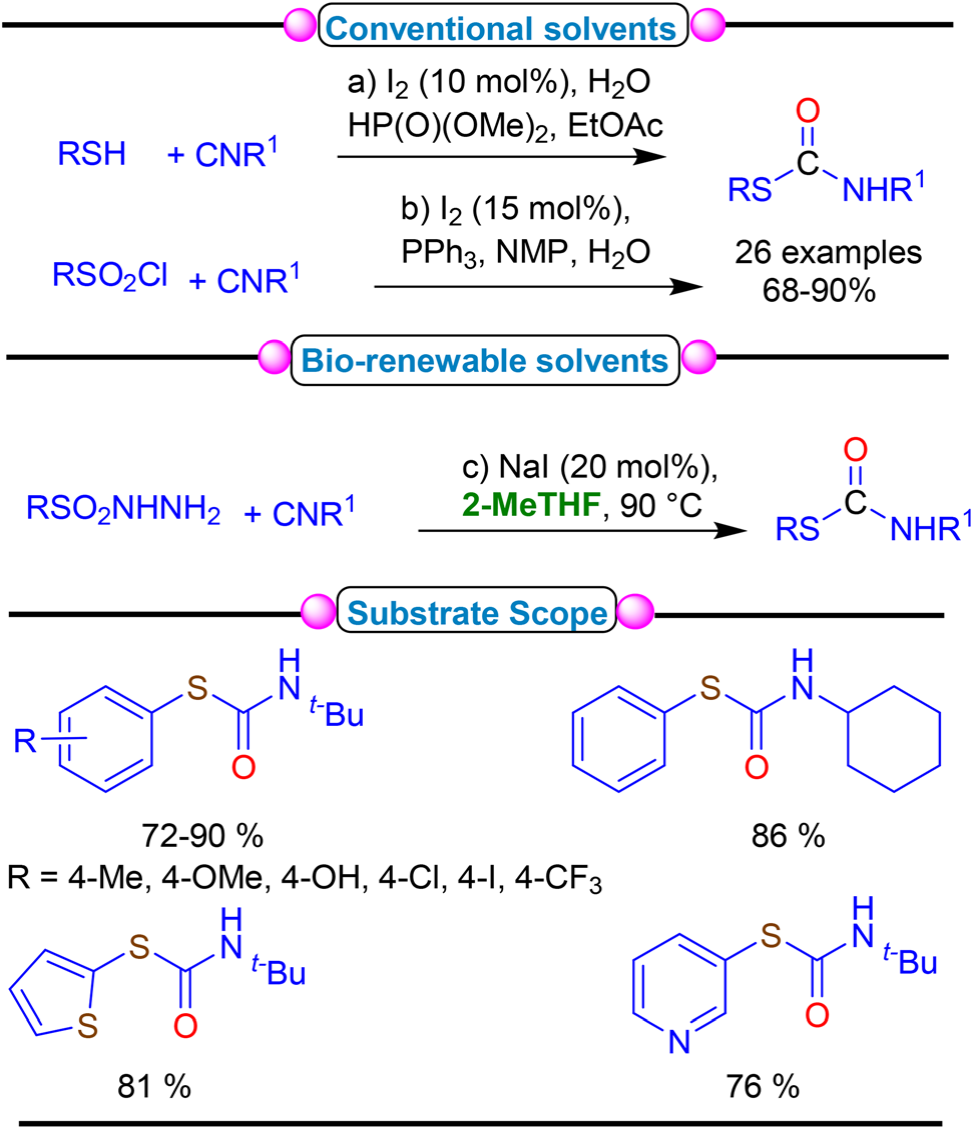
Synthesis of *S*-thiocarbamates.

*tert*-Butyl nitrite is an important reagent for the synthesis of key intermediates in organic, pharmaceutical, and agrochemical applications.^144–146^ Wei *et al.* reported an iodine-catalysed multicomponent synthesis of thiocarbamates from aryl sodium sulfinates, isonitriles, and water using dimethyl phosphite as a reductant in ethyl acetate (Scheme 19a).^147^ Similarly, He *et al.* developed an I_2_-catalysed protocol employing sulfonyl chlorides and triphenylphosphine in *N*-methylpyrrolidone (Scheme 19b).^148^ In 2019, Bao *et al.* reported an alternative approach for the synthesis of alkyl and (hetero)aryl *S*-thiocarbamates using benzenesulfonyl hydrazine and *tert*-butyl isocyanide, with NaI (10 mol%) as catalyst in 2-MeTHF under air at 90 °C (Scheme 19c).^149^ This method avoids the use of phosphorus-based reductants and generates benign byproducts such as H_2_O and N_2_, while showing good substrate scope and regioselectivity.

**Scheme 20:**
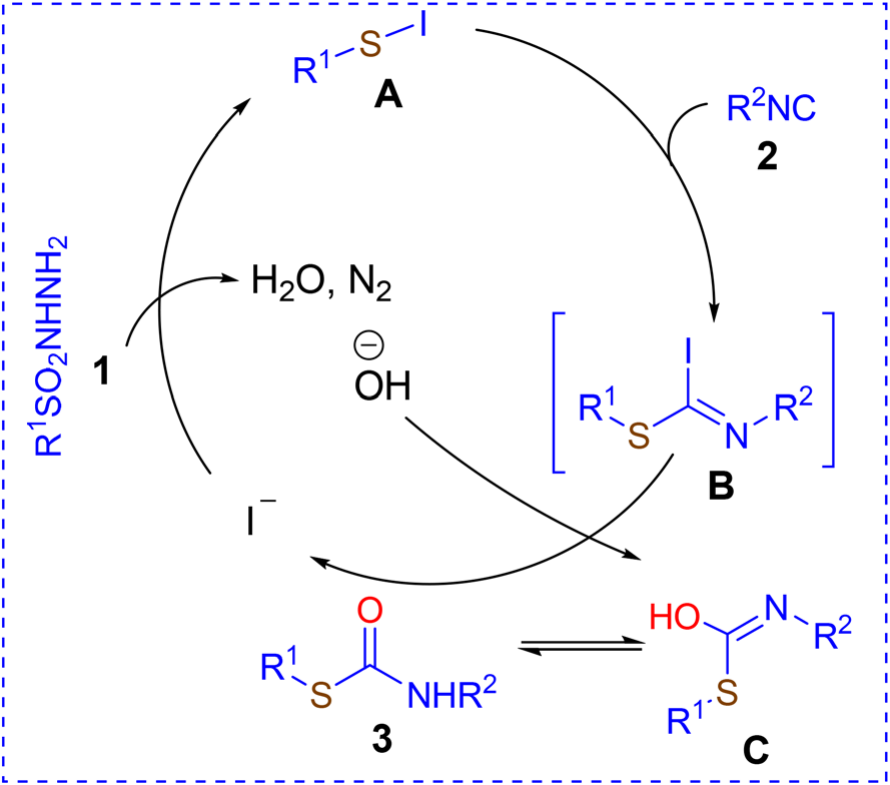
Mechanistic pathway of NaI-mediated formation of thiocarbamates.

In the proposed reaction mechanism (Scheme 20), initially sulfonyl hydrazine 1 reacts with a catalytic amount of iodine, which results in the formation of R–S–I A intermediate with the concomitant release of H_2_O, N_2_ and hydroxide (OH^−^) ions. Subsequently, the intermediate A react with iso-nitrile 2 generated the intermediate B, which is then attacked by *in situ* generated OH^−^ to produce carbamimidothioate intermediate C and the iodide ion is regenerated back to the catalytic cycle. In the last step, the intermediate C undergo tautomerization (*i.e.*, migration of a proton) to form the target product 3.

**Scheme 21:**
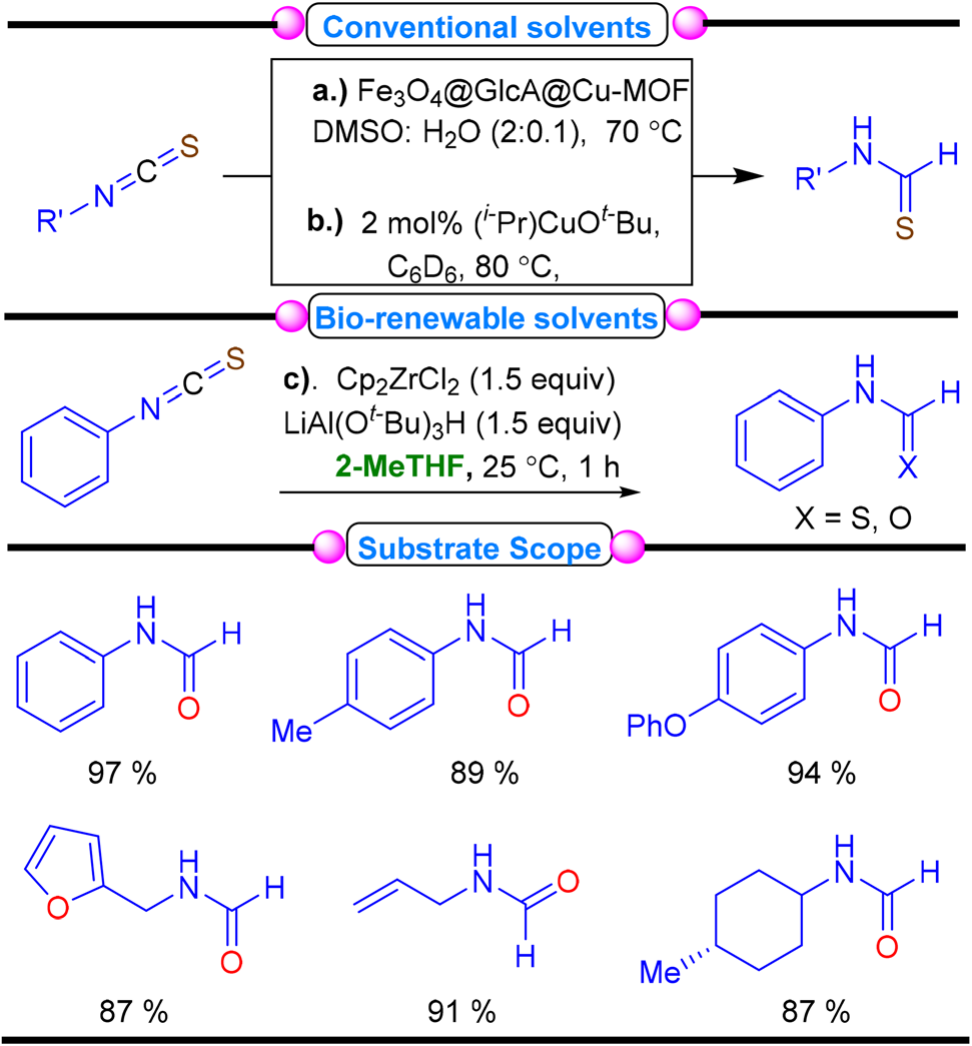
Different synthetic routes for the preparation of formamides.

The synthesis of thioformamides from isothiocyanates using molecular iodine has been reported in 2-MeTHF as a solvent (Scheme 21c). Such transformations are often achieved using homogeneous or heterogeneous catalysts under mild conditions (Scheme 21a and b).^150–152^ In 2016, Pace *et al.* developed a method employing an *in situ* generated Schwartz's reagent (Cp_2_ZrHCl) for the conversion of isothiocyanates to thioformamides in sustainable solvents (Scheme 21c).^153^ This approach afforded high yields, excellent chemoselectivity, and retention of stereochemistry, even in the presence of sensitive functional groups. Solvent screening indicated that 2-MeTHF performed comparably or better than conventional solvents such as THF under the reported conditions.^154^

**Scheme 22:**
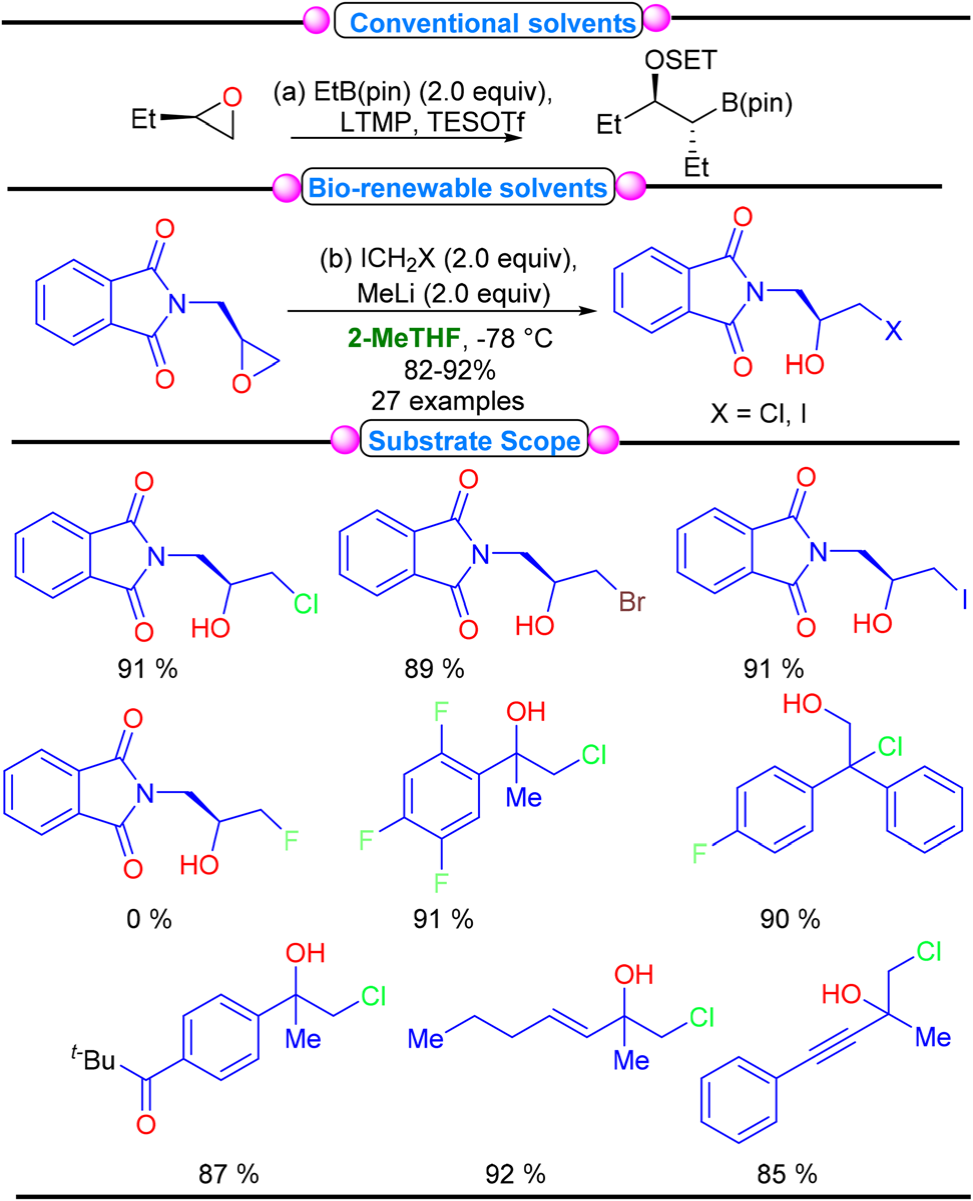
Epoxide ring-opening giving β-halohydrins.

Lithium monohalocarbenoids are versatile C1 synthons capable of participating in both nucleophilic and electrophilic transformations, enabling homologation processes.^155–157^ One such application involves the use of terminal lithiated epoxides for the homologation of boronic esters (Scheme 22a).^158^ However, their utility is often limited by competing α-elimination pathways. Ielo *et al.* addressed this challenge by developing a method that promotes controlled epoxide ring opening to generate β-halohydrins *via* a Kirmse-type elimination (Scheme 22b).^159^ Solvent screening, including conventional ethers (THF, Et_2_O) and biomass-derived alternatives (CPME, 2-MeTHF), showed that reaction outcomes are sensitive to solvent effects. Under the optimized conditions, high yields and good regioselectivity were obtained with a range of epoxides and carbonyl compounds, without racemization of enantiopure substrates.

**Scheme 23:**
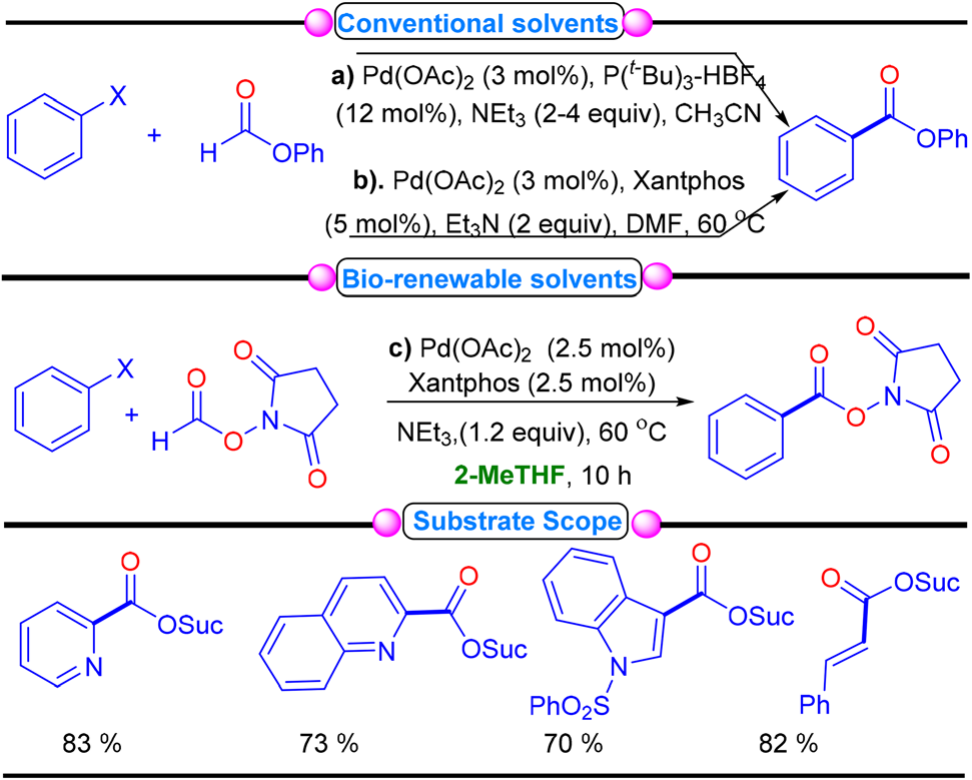
Palladium catalysed NHS esters synthesis.

2-MeTHF has also been explored as a solvent for the synthesis of *N*-hydroxysuccinimidyl (NHS) esters. In 2012, Manabe *et al.* reported an organocatalytic one-pot cross-coupling strategy using MeCN as the reaction medium (Scheme 23a).^160^ In the same year, Y. Tsuji developed a related one-pot protocol in DMF (Scheme 23b).^161^ Subsequently, Levacher *et al.* (2015) described a palladium-catalysed approach employing Pd(OAc)_2_ and trimethylamine in a biomass-derived 2-MeTHF solvent (Scheme 23c).^162^ This method utilizes NHS formate as a CO source and enables the conversion of aryl, vinyl, allyl, and benzyl halides into the corresponding NHS esters, affording moderate to excellent yields with a broad substrate scope.

**Scheme 24:**
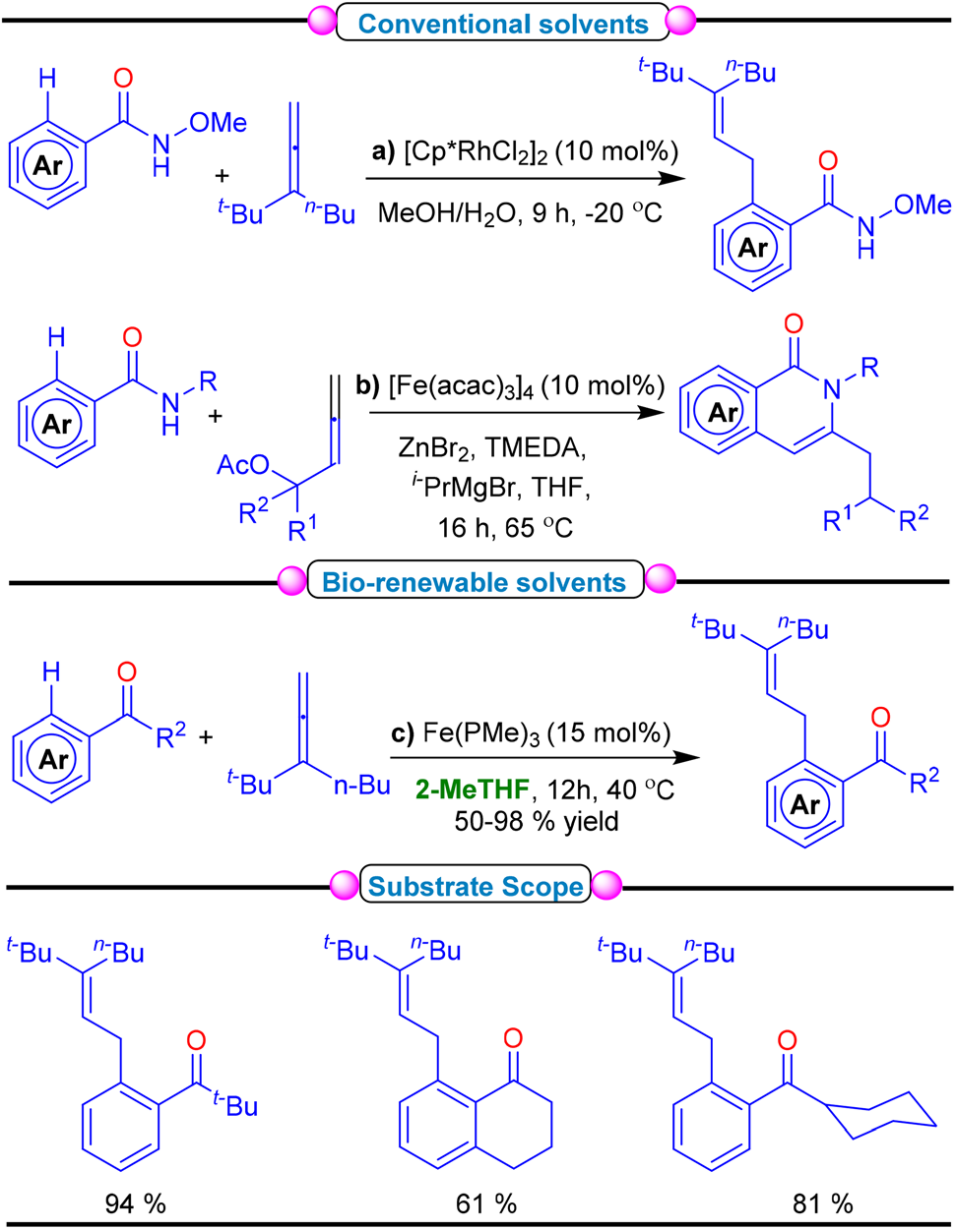
Robustness of metal-catalysed C–H activation with allenes.

More recently the article published by Ackermann *et al.*^163^ presents a sustainable method of iron-catalysed hydroarylation of allenes under mild reaction conditions (Scheme 24c). Audits regarding the matter of arising eccentric biobased solvents for C–H bond functionalization have been reported by Shengming Ma in 2012 using Rh-catalyst in aqueous alcoholic solvent (Scheme 24a)^164^ and Ackermann *et al.* in 2018 using an iron catalyst in THF solvent (Scheme 24b)^165^ with a required stoichiometric quantity of Grignard reagents. The replacement of THF by a biobased solvent (2-MeTHF) in C–H functionalization of allenes and aromatic carbonyl compounds proceeds with high efficiency and controlled regioselectivity at the *ortho*-position even in the proximity to the weakly-coordinating carbonyl group of the substituted phenones. In terms of synthetic potential, a broad range of substrate scope has been developed by employing various phenones and allenes in 12 h at 40 °C.

**Scheme 25:**
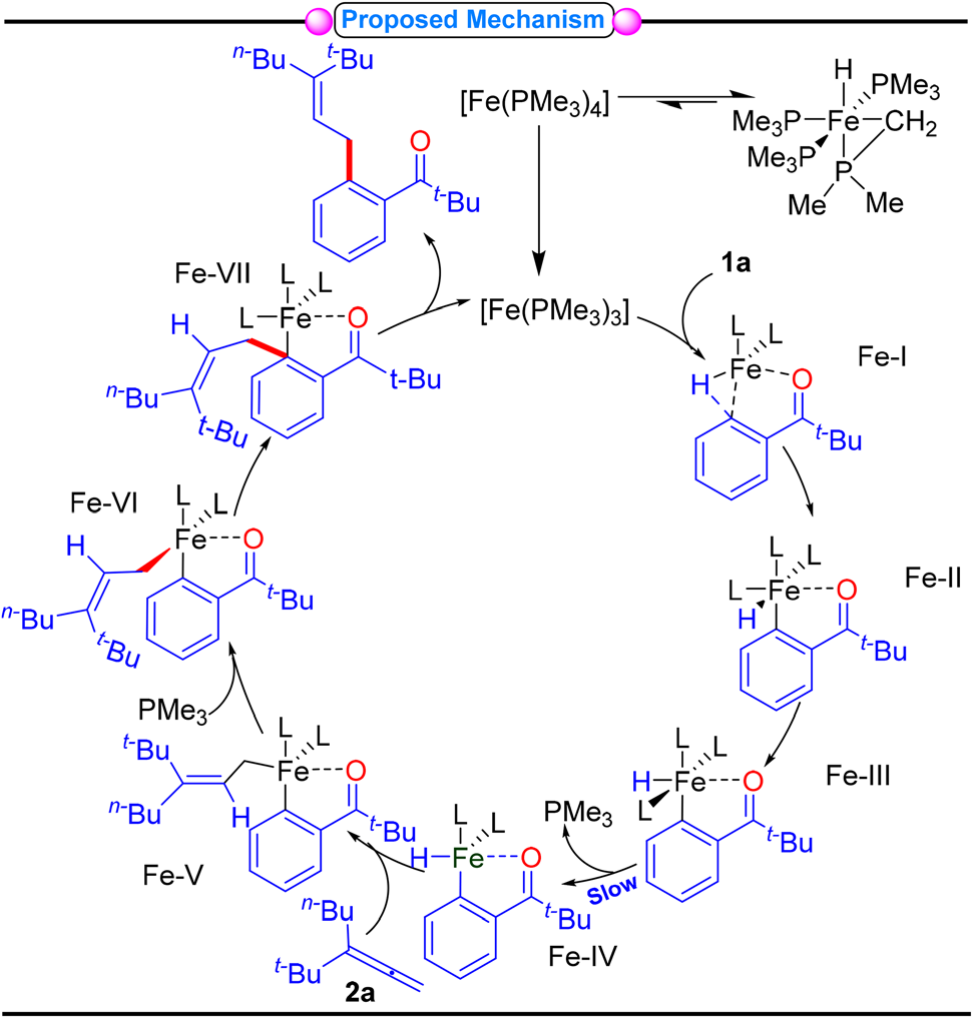
Proposed mechanistic pathway of iron-catalysed allylation of phenones with allenes.

Ackermann *et al.* suggested that the catalytic cycle (Scheme 25) be started by the formation of the co-ordinatively unsaturated intermediate B*via* the de-coordination of one PMe_3_ ligand, based on the experimental, kinetic, and computational findings. The proposed mechanism illustrates that the pre-catalyst A and unsaturated species B are in equilibrium, which results in the induction time that is shown in experiments. Then, phenone 1 weakly coordinates B to create the agostic complex Fe-I. This complex then swiftly undergoes oxidative addition to produce iron(ii) hydride intermediate Fe-II, which isomerizes to Fe-III very quickly. Fe-III gradually loses PMe_3_, becoming the coordinatively unsaturated complex Fe-IV, and this step has been assigned as the rate-determining step. Next, the intermediate Fe-V is generated through the coordination and migratory insertion of the allene. The desired product is liberated by phosphine coordination, ligand exchange, reductive elimination, and regeneration of [Fe(PMe_3_)_3_] as the active catalyst.

**Scheme 26:**
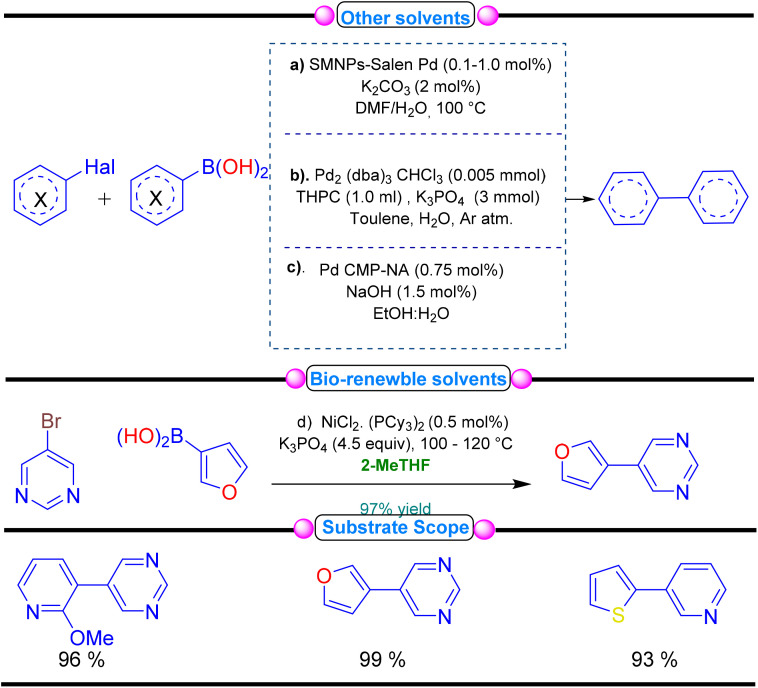
Synthesis of heterocyclic biphenyls by Suzuki–Miyaura cross-coupling reactions.

Palladium catalysed cross-coupling reactions are used extensively in both industrial and academic laboratories on a large scale for the formation of C–C bonds. Various synthetic approaches have been reported for the preparation of biaryl scaffolds *via* Suzuki–Miyaura cross-coupling reactions. In 2012, Jin *et al.* reported the use of a Schiff base-supported salen-Pd nanoparticle functionalized with ferrite as a catalyst in Suzuki–Miyaura coupling reaction^166^ employing DMF as the solvent (Scheme 26a). In 2002, McNulty *et al.*^167^ described the use of a palladium complex for this purpose in toluene as solvent in an inert atmosphere (Scheme 26b). On the other hand, more recently, Zang *et al.* developed a Pd/CMPs catalyst for the synthesis of biaryls through Suzuki–Miyaura coupling reaction in an ethanolic solvent at 80 °C (Scheme 26c).^168^ However, apart from the use of commercial solvents, Ramgren *et al.* in 2013 investigated the reactivity of a nickel complex as a catalyst for the synthesis of biaryls using biomass-derived 2-MeTHF as a solvent *via* the Suzuki–Miyaura coupling method.^169^ This reaction condition is tolerated with an extensive range of substrates, including both heterocycles and coupling partners, producing a yield up to 97% in gram-scale synthesis.

**Scheme 27:**
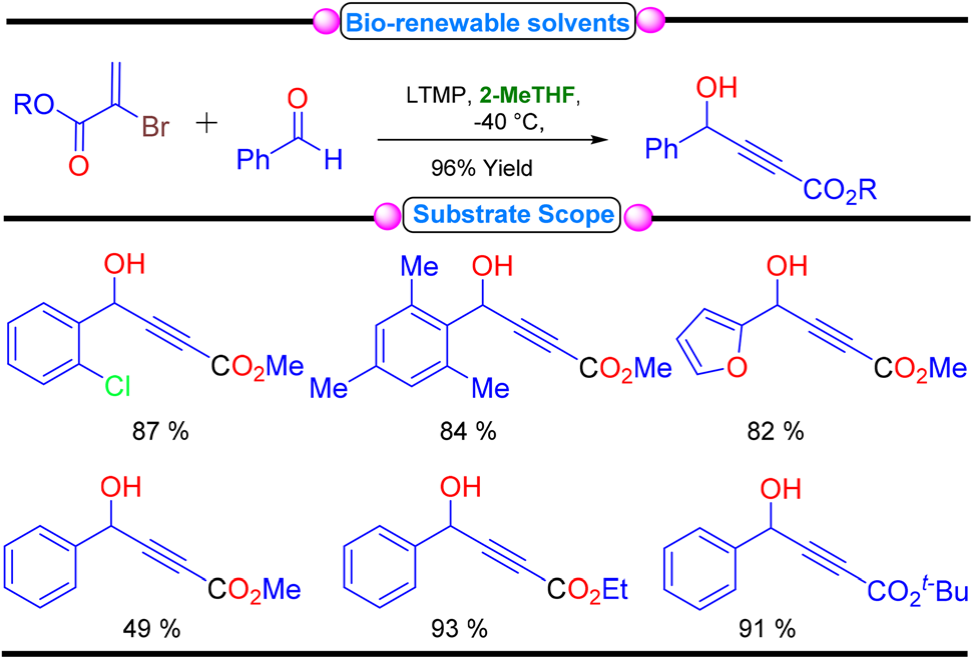
Synthesis of γ-hydroxy-α, β-acetylenic esters.

A report by Holzer *et al.* showed a green approach to synthesise γ-hydroxy-α,β-acetylenic esters using lithium 2,2,6,6-tetramethylpiperidide (LTMP) as a base in 2-MeTHF as an environmentally acceptable solvent (Scheme 27).^170^ Based on this method, a propiolate nucleophile has been generated through a dehydrohalogenation process under mild reaction conditions, which may then react directly with aldehydes before quenching. This approach avoids the instability of the propiolate nucleophile compared to previous methods.^171–175^ Employing 2-MeTHF as a solvent delivers a yield of 94% in a short time of 1.5 hours, which is a more precise method compared to other traditional solvents.

**Scheme 28:**
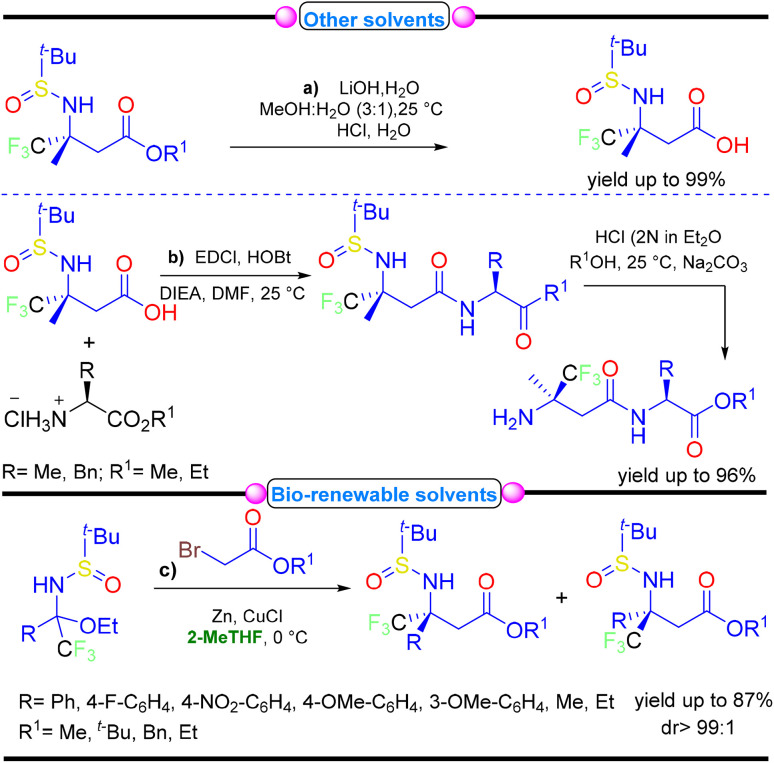
Synthesis of β-trifluromethyl β-amino esters.

2-MeTHF has been used as solvent in C–C bond-formation reactions, such as β-amino acids, which are subunits of beta, as well as alpha/beta peptides,^176,177^ are crucial building blocks of many medicinally active scaffolds and peptidomimetics.^178^ It is noteworthy to mention that β-trifluromethyl β-amino acids were studied in detail in recent years. Diverse diastereoselective and enantioselective synthetic protocols of the mentioned scaffolds are reported.^179,180^ F. Grellepois in 2013 reported a highly efficient, stereoselective imino-Reformatsky reaction for the synthesis of β-trifluromethyl β-amino esters (Scheme 28c).^181^ This, novel synthetic protocol was the first method for synthesising β-trifluoromethyl β-amino acid derivatives containing a quaternary stereo-centre adjacent to the amine function in the desired scaffolds. High diastereoselectivity and moderate to excellent yield were achieved from this synthetic protocol by using 2-MeTHF as a biomass-derived green solvent. Generally, Reformatsky reagent was added to α-aryl (alkyl) α-trifluoromethyl *N-tert*-butanesulfinyl hemiaminals in moderate reaction conditions to access the desired compound. In a further extension of this reported protocol, C and N-terminal coupling with natural amino acids was achieved. In addition, hetero di/tripeptides were also synthesised using this sustainable, mild synthetic method (Scheme 28a and b).

**Scheme 29:**
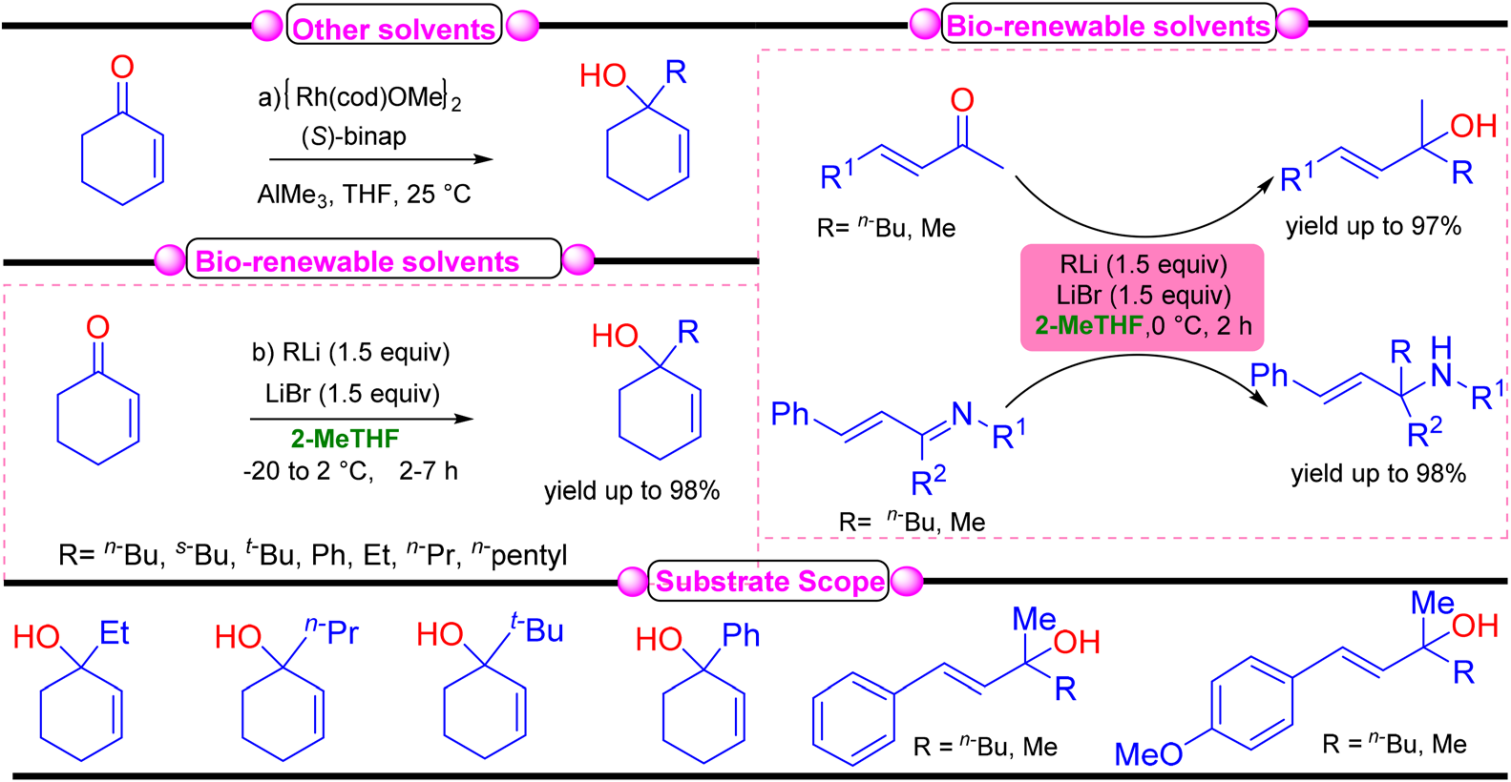
Regioselective synthesis of allylic alcohols.

Generation of tertiary carbon centres by using metal or metal free conditions in various polar, polar aprotic, hydrocarbon and ethereal solvents has become a point of interest.^182*a*,*b*^ Examples of these reactions involving the use alkyl lithium reagents, Grignard reagents, and metal hydride reagents and Lewis acids.^182*c*^ By the replacement of hydrocarbon and ethereal solvents with the 2-MeTHF has been successfully bring into the service as a multitude of organometallic reactions, these sustainable solvents often acting as a swing for the replacement of THF, 1,4-dioxane, *etc.* Pace *et al.*^183^ in 2011 demonstrated that LiBr in 2-MeTHF shows very high regioselectivity towards 1,2-addition of aldehydes, ketones, and α,β-unsaturated imines to give a variety of allylic alcohols and amines has been demonstrated. This method utilises mild reaction conditions, high yield, and high regioselectivity, which were the crucial features of this novel synthetic protocol (Scheme 29b). Furthermore, 1,4-addition products were not detected by ^1^H NMR, and the reactions were conducted at 0 °C in 2 h, making this an attractively simple method. Similar reactions were conducted in THF solvent (Scheme 29a), which took a longer reaction time and gave poorer yields when compared to 2-MeTHF (*e.g.*, 6 h 79% yield).

**Scheme 30:**
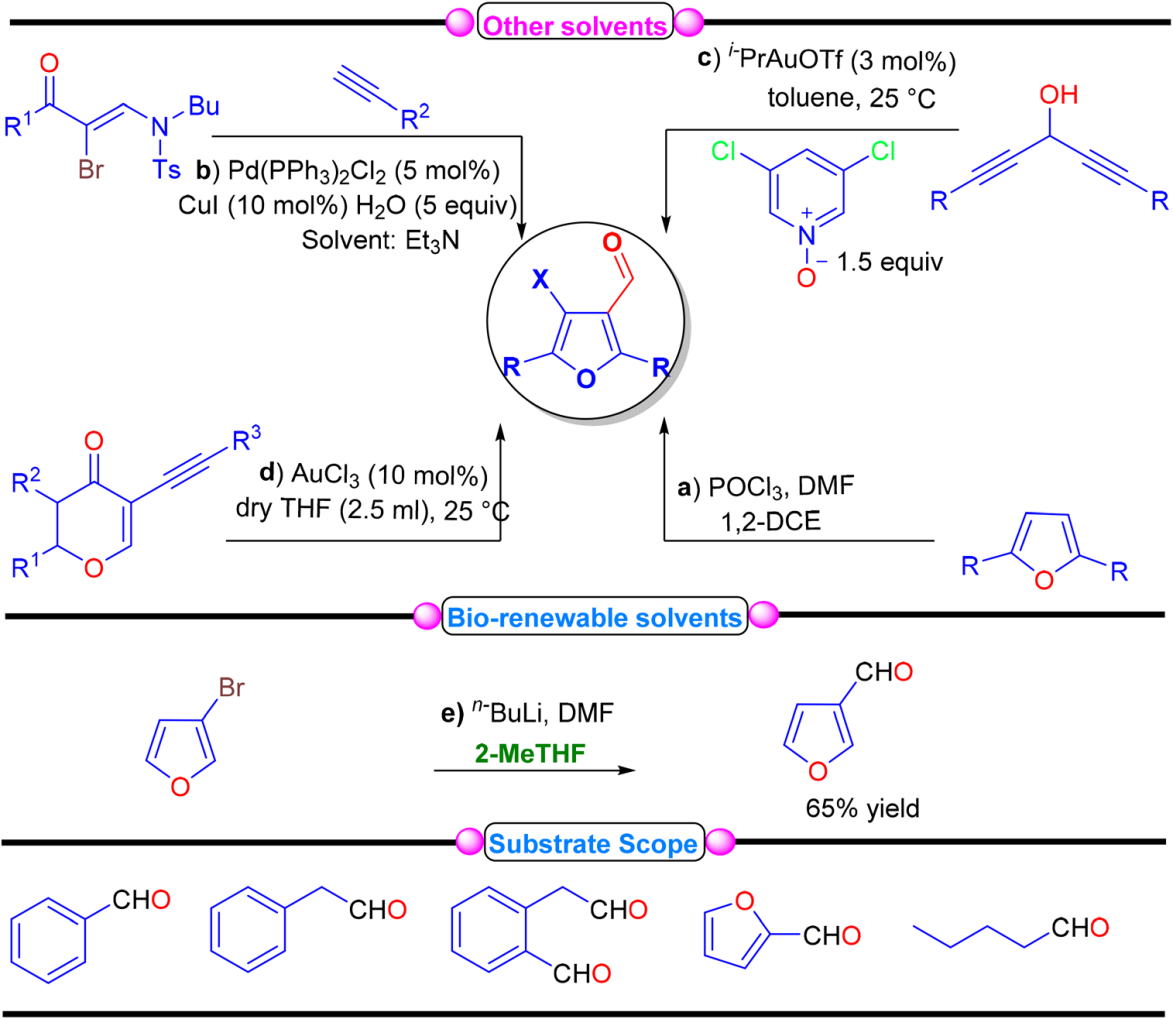
Hydroformylation of alkenes and alkynes.

A solvent-reagent guide for aldehyde formation from alkynes, alkenes by a metal catalysed reaction or by employing Vilsmeier–Haack reaction has also been recently developed. Traditionally, 3-formyl furans were prepared either by direct formylation of furans or by coupling and transformation of other organic moieties. One of the examples of direct formylation involves a Vilsmeier–Haack reaction by using POCl_3_ in DMF and 1,2-DCE (Scheme 30a).^184^ From the past, some methods were devised to synthesise aldehyde functional group moieties. In 2011, Yang *et al.* also developed an efficient method *via* palladium/copper-catalysed approach to the synthesis of highly substituted 3-formyl furans (Scheme 30b).^185^ Later, A. S. K. Hashmi and Indrajit Das uses gold catalyst in toluene and THF solvent to carry out this transformation (Scheme 30c and d).^186,187^ In 2007, David F. Aycock has replaced THF and diethyl ether in one of the organometallic reactions by 2-MeTHF for the synthesis 3-furaldehyde (Scheme 30e)^188^ using DMF as a C1 synthon and *n*-BuLi as a base. It was also reported that 2-MeTHF has been found to be more promising than toluene in extracting polar compounds from water mixtures.^203^

**Scheme 31:**
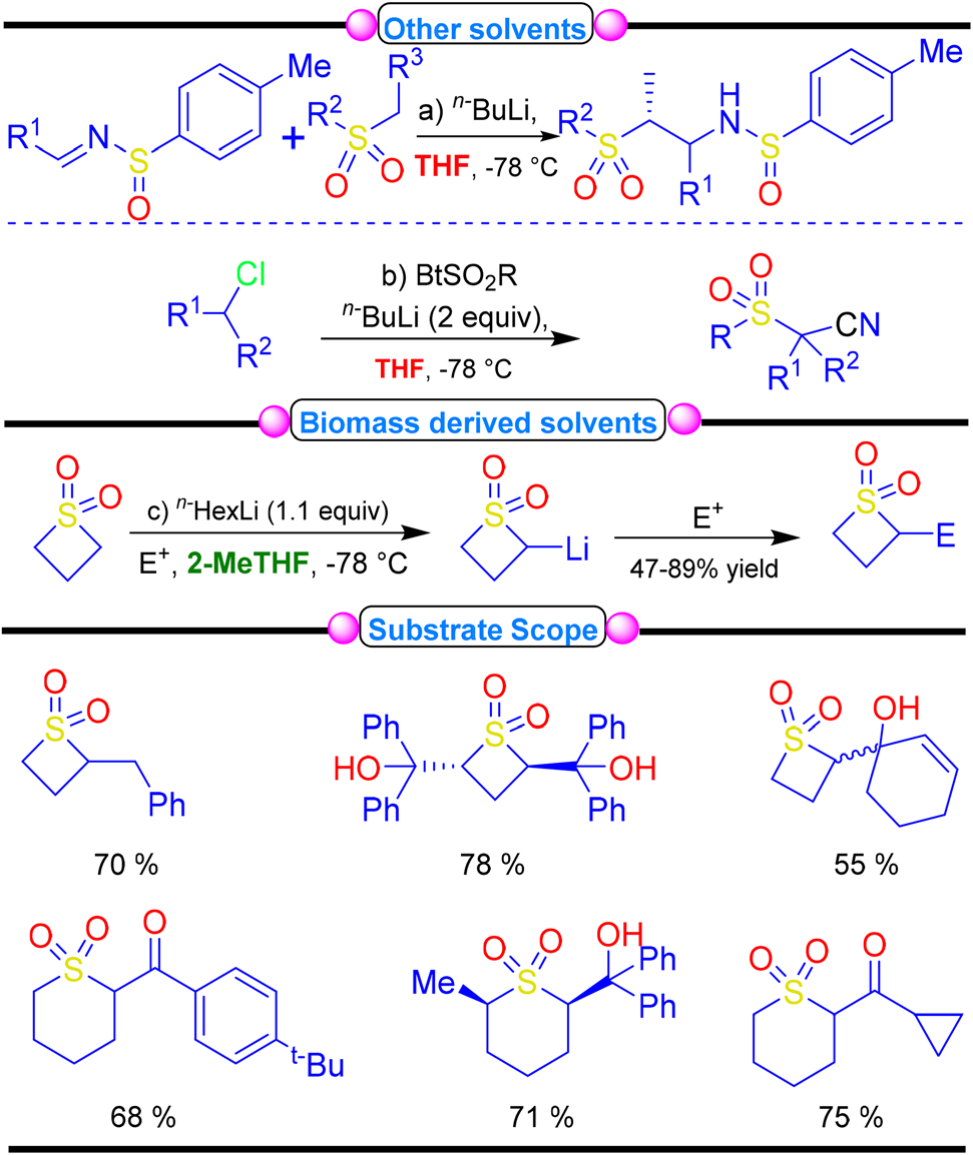
Functionalization of substituted thietane 1,1-dioxides.

Further the synthesis of sulfones was devised by Katritzky *et al.* by a readily available *N*-acylbenzotriazoles with acyclic and alicyclic sulfones to produce equivalent α-keto sulfones (Scheme 31a).^189^ Later, the authors have also observed that readily accessible *N*-benzotriazoles produce *R*-cyanoalkyl sulfones when they were combined with various nitriles, reactive heteroaromatics, alkylheteroaromatics, sulfones, and esters in THF solvent (Scheme 31b).^190^ Velazquez *et al.* developed a highly stereoselective synthesis of α-amino sulfones and sulfonamides by adding sulfonyl anions to chiral *N*-sulfinyl imines.^191^ In 2017, Parisi *et al.* have scrutinized the reactivity of cyclic sulfones with six and four membered rings (*i.e.* thietanes and thiopyrans) in sequences including electrophilic entrapment lithiation strategy (Scheme 31c).^192^ When the functionalization of heterocycles is at C_2_ or C_2_, C_4_ (C_6_), a similar reactivity profile has been observed. Several C_2_ or C_2_, C_4_, (C_6_), substituted cyclic sulfones were synthesised with moderate to excellent (55–85%) yields. The synthesised procedure is compatible with Csp^2^ coupling processes and transmetallation, according to preliminary data.

**Scheme 32:**
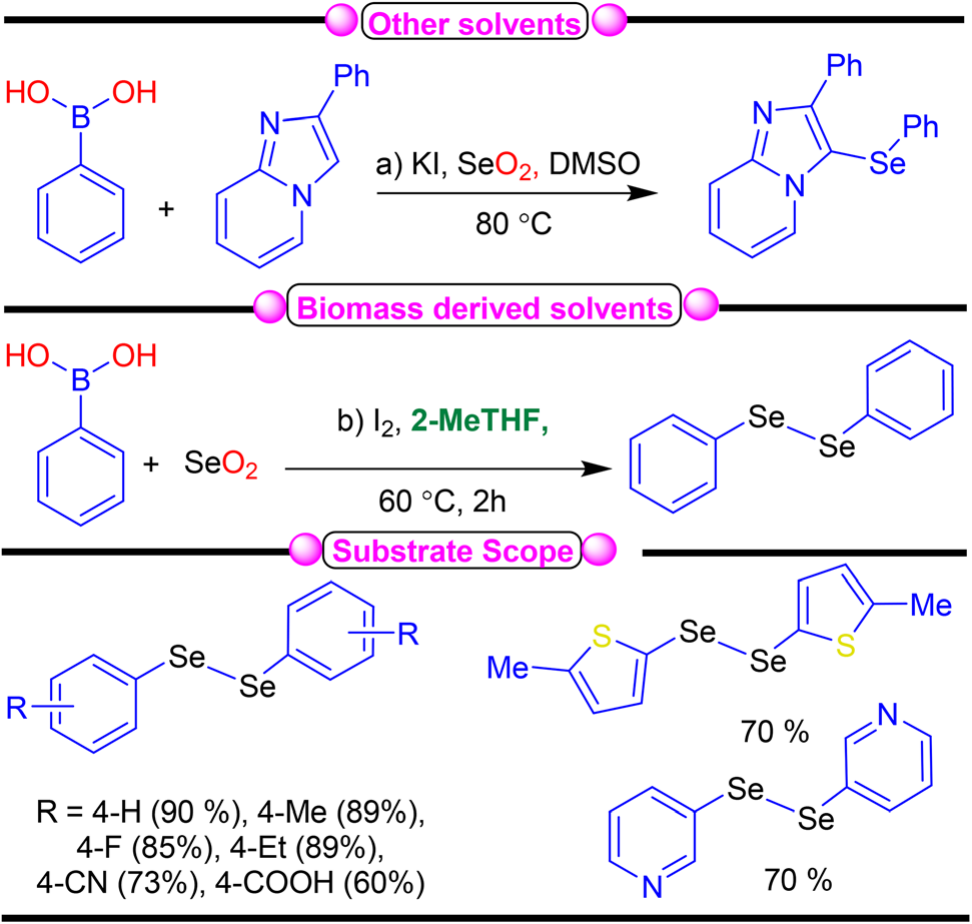
Methods for organo-selenium synthesis using SeO_2_ as the selenium source.

Besides C–S bonds, the research extends towards the formation of C–Se bond by employing biomass-derived solvents, with several reports available in the literature,^193^ in 2019, Ren. *et al.* reported the formation of C–Se bond using DMSO as a reaction medium (Scheme 32a).^194^ Later in 2019 K. Zhao and his team demonstrated the organic transformation of benzene boronic acids in biomass-derived solvents for the construction of symmetrical diselenide Se–Se bond. The potential of reaction conditions has been demonstrated by the synthesis of various symmetrical diselenide compounds in excellent yields.^195^ Furthermore, they have utilised 1 mmol% of iodine in 2-MeTHF at 80 °C to carry out this transformation of the symmetrical Se–Se bonds (Scheme 32b).

**Scheme 33:**
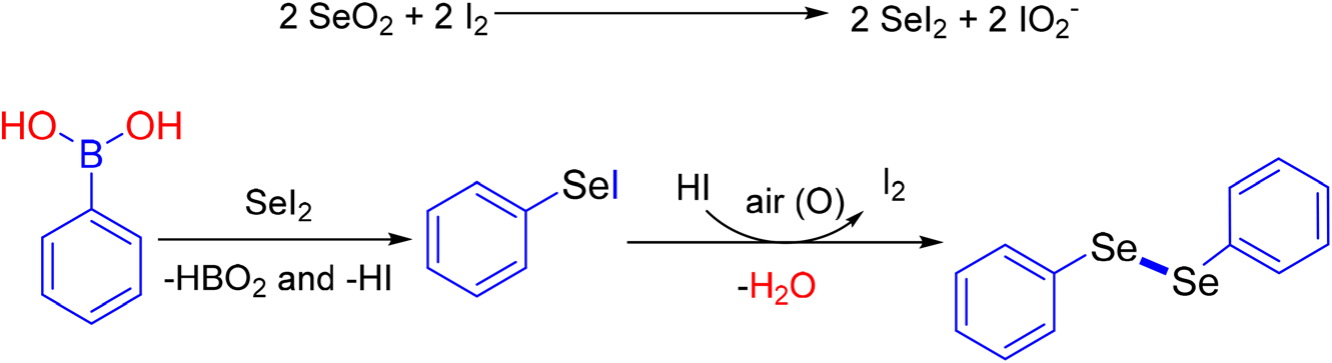
Plausible mechanistic pathway for the synthesis of diaryldiselenides.

Experiments with free radical trapping were carried out to propose a plausible reaction pathway (Scheme 33) that described a process for the simple synthesis of aryldiphenyl diselenides mediated by iodine. Initially, the nucleophilic aryl group from the phenylboronic acid combines with selenium iodide to produce phenylselenium iodide, which was identified by GC-MS study. Furthermore, the molecular iodine and selenium dioxide are reported to react to make selenium iodide and iodide. Afterwards, diphenyl diselenide was produced when it was reduced by HI. Ji and Lumb have also reported a similar Pomeranz–Fritsch cyclization towards the synthesis of 1,2-dihydroisoquinolines under Fujioka/Kita conditions.

**Scheme 34:**
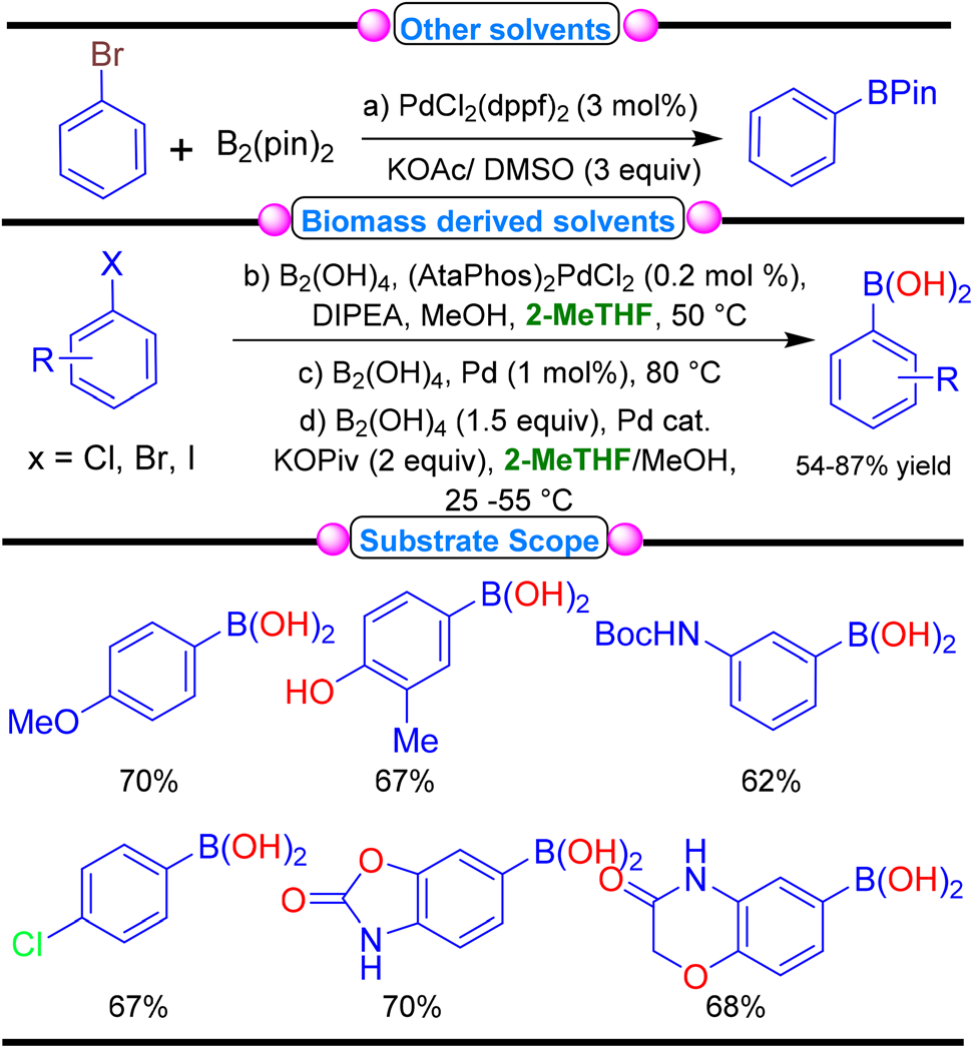
Palladium-catalysed borylation of aryl halides.

One of the two main “workhorses” in medicinal chemistry, the Suzuki–Miyaura coupling of aryl and heteroaryl (pseudo)halides with organoboron coupling partners is used more frequently than any other cross-coupling reaction on both the discovery and process scales^196–198^ In 1995, N. Miyaura developed a palladium catalysed cross-coupling reaction by employing haloarenes and pinacol ester of diboronic in DMSO solvent (Scheme 34a).^199^ Later, Fouggatakis *et al.* created a productive one-pot palladium [(AtaPhos)_2_PdCl_2_] catalysed borylation process using B_2_(OH)_4_ (Scheme 34b).^200^ Tetrahydroxydiboron (B_2_(OH)_4_) which directly generates aryl boronic acids from the appropriate aryl halide in one step, was created by Molander and Dreher (Scheme 34c).^201^ Under mild reaction conditions with green solvent Me-THF, two complementary Pd-based systems have been created by Frantz and team to efficiently borylate aryl bromides and iodides or aryl chlorides (Scheme 34d).^202^

**Scheme 35:**
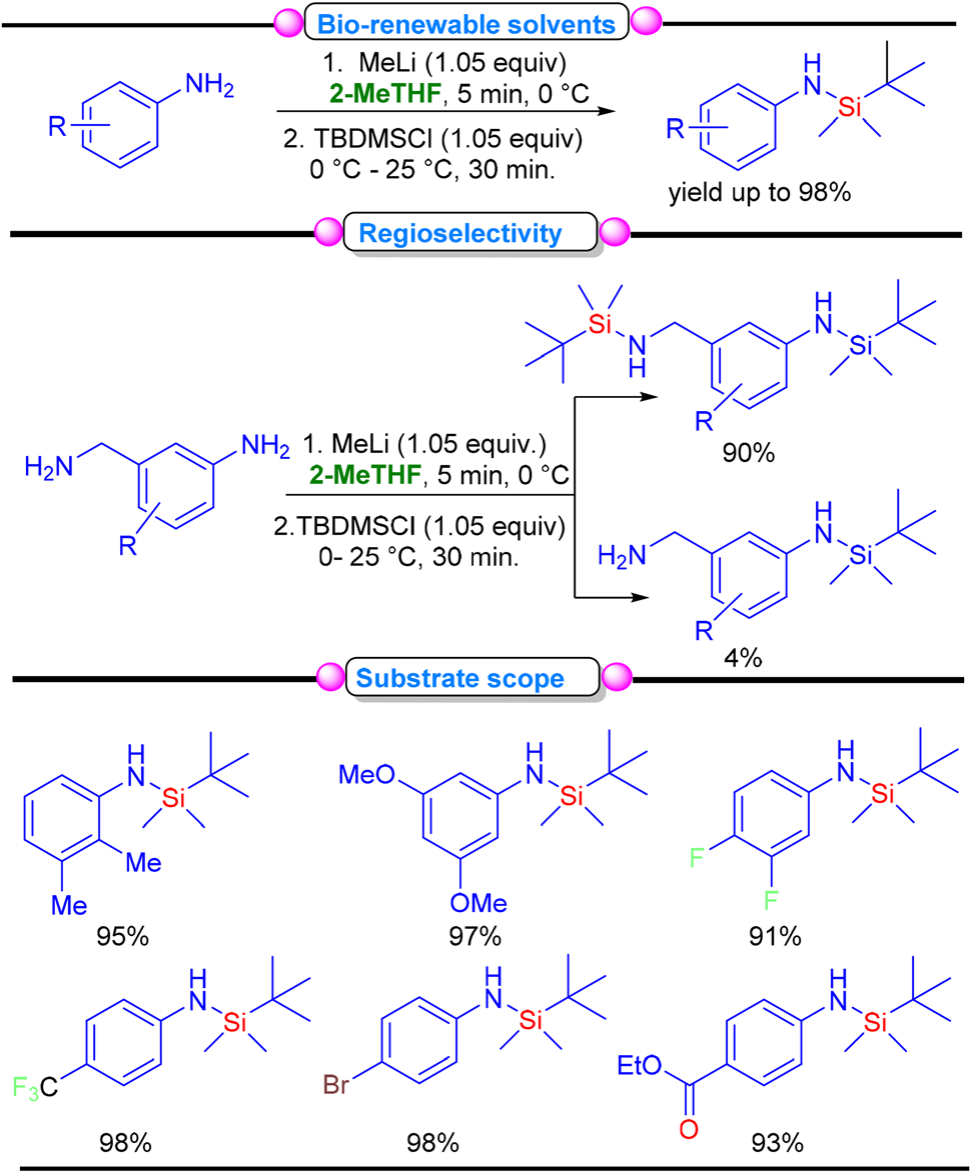
*N*-TBS protection of substituted anilines.

In synthetic organic chemistry, protection of a particular functional group among others is essential to fruitfully drive a reaction without any hamper or not to allow any other functional group in a particular reaction condition.^203^ Therefore, in pursuit of developing a more sustainable synthetic protocol, this protection–deprotection method violates the principle of green chemistry.^204^ In this context, it is noteworthy to mention that the protection of the amino functional groups in the course of the reaction is crucial as many biologically active molecular scaffolds contain these functional groups.^205,206^ In 2011, Pace *et al.*^207^ reported a highly efficient synthesis of *N*-TBS as the amine-protecting group in the presence of biomass-derived solvent 2-MeTHF at mild reaction conditions. The desired compound was obtained in high yields with selectivity upon following a sustainable synthetic protocol (Scheme 35). In this approach apolar aprotic solvents shows less compatibility in these reactions whereas polar aprotic solvents like 2-MeTHF led to higher yields. On the contrary, the use diethyl ether and THF results in some unknown impurities in the product and reduces yield when the reaction is carried above −50 °C. This characteristic feature of 2-MeTHF is because of its higher stability in strongly alkaline media when compared to other ethereal solvents.

**Scheme 36:**
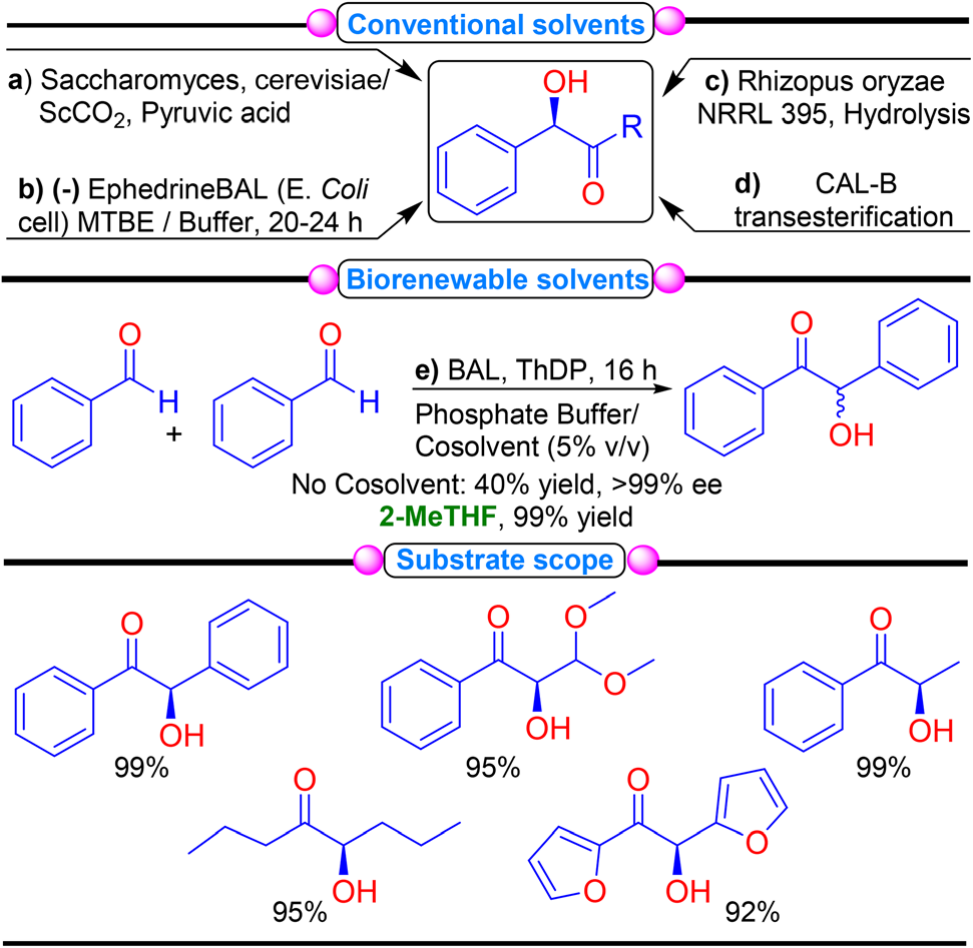
Bio-catalysed synthesis of α-hydroxy ketones.

In past decade, bio-catalysed or enzyme catalysed synthetic transformations have been an integral part of scientific research due to its high regioselectivity, enantioselectivity and more importantly mild reaction conditions.^208–210^ In this context, medicinally viable scaffolds, α-hydroxy ketones have been accessed through the involvement of ThDP-Lyases (thiamine-diphosphate dependent lyases) as a biocatalyst for the carboligation of aldehydes.^211,212^ Smallridge *et al.* reported pyruvate decarboxylase (PDC) catalysed synthesis of (−)-ephedrine in supercritical carbon dioxide (Scheme 36a).^213,214^ de Maria *et al.* devised benzaldehyde lyase (BAL) catalysed biphasic systems in the presence of *E. coli* whole cells and MTBE/buffer to access α-hydroxy ketones (Scheme 36b).^215,216^ Demir *et al.* and Lee *et al.* reported hydrolase catalysed efficient kinetic resolutions of alkyl/aryl α-hydroxy ketones (Scheme 36c and d).^217,218^ Furthermore, de Maria *et al.* in 2010 reported the efficient synthesis of α-hydroxy ketones in the presence of benzaldehyde lyase (BAL). MTBE and DMSO were used as a second organic phase during the course of reaction, but at the time of purification DMSO faces difficulties during separation process and MTBE has been dropped due to its petrochemical origin. Thus, 2-MeTHF acts as a promising candidate to carry out this biocatalysed organic transformation by using both aromatic and aliphatic moieties.^219^ It was observed that high yield and excellent enantioselectivity were possible due to the use of 2-MeTHF as cosolvent in the biphasic system (Scheme 36e). In addition, they reported the use of 2-MeTHF as extracting solvent like ethyl acetate and DCM.

**Scheme 37:**
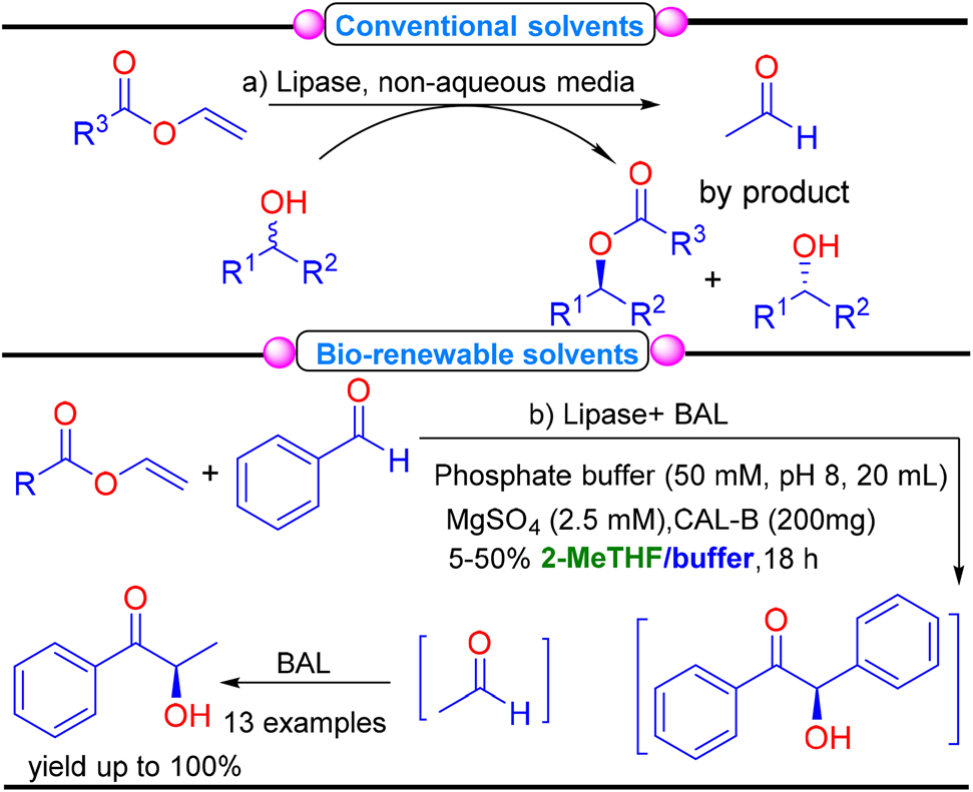
Enantioselective C–C bond formation using benzaldehyde ligase.

Lipases are vital biocatalyst for dynamic kinetic resolution of alcohols and amines in non-aqueous media, where acetaldehyde forms as a byproduct (Scheme 37a).^220,221^ The byproducts can be recovered from the reaction mixture and employed as reagent in various transformations.^222^ It is noteworthy to mention that CH_3_CHO is an important chemical for diverse chemical transformations, but it has high volatility and reactivity even with itself, which makes it less feasible in synthetic transformations.^223–225^ de Maria *et al.* in 2012 reported that lipase B from *Candida antarctica* (CAL-B) could be viable for the efficient production of acetaldehyde not only in organic media but also in biphasic systems and aqueous media (Scheme 37b).^226^ To further utilize this generated acetaldehyde, one-pot cross-carboligation of acetaldehyde and benzaldehyde was carried out in the presence of benzaldehyde lyase (BAL) to access 2-HPP (2-hydroxypropiophenone). Furthermore, the synthesis of 2-HPP was assessed in the presence of 5% v/v 2-MeTHF bio-based cosolvent in aqueous media.

**Scheme 38:**
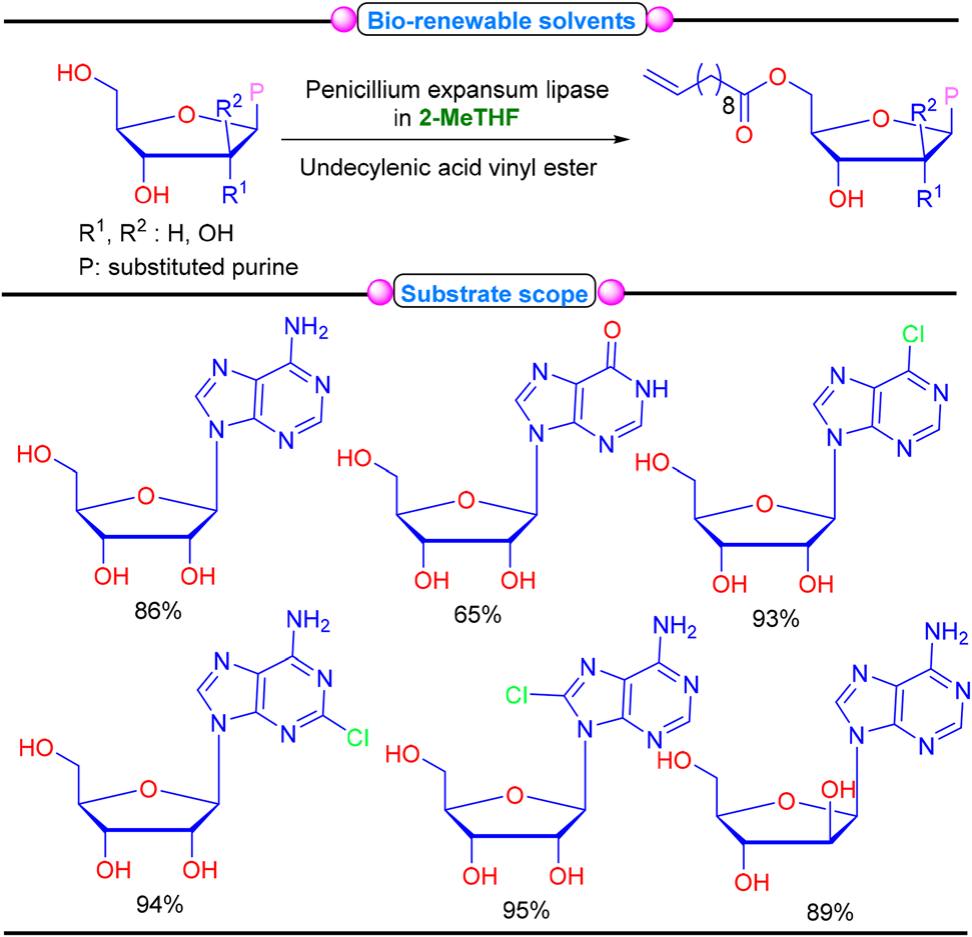
Synthesis of 5-undecylenic acid esters in 2-MeTHF as a bio-renewable solvent.

Although the majority of biocatalytic transformations are performed in organic solvents like 2-MeTHF because of their high resilience. Some of the enzyme catalysed modification has also been observed to be a solution to this issue. Though THF has been a vital solvent for these transformations, recent studies have shown alternative use of 2-MeTHF as the biomass-derived solvent^227,228^, which has a higher boiling point, no toxicity, and a higher tolerance to HCl than THF.^229^ Inspired by the enzyme catalysed modification of nucleosides, Zong *et al.* in 2012 reported *Penicillium expansum* lipase catalysed regioselective acylation of 8-chloroadenosine (8-Cl-Ado)^230^ in the presence of green solvent 2-MeTHF with a yield up to 95% at a temperature of 35 °C in 3 hours (Scheme 38). The lipase enzyme depicts much better thermostability and twice enhances its catalytic activity in 2-MeTHF solvents compared to other traditional solvents.

### 4.2. γ-Valerolactone (GVL)

**Scheme 39:**
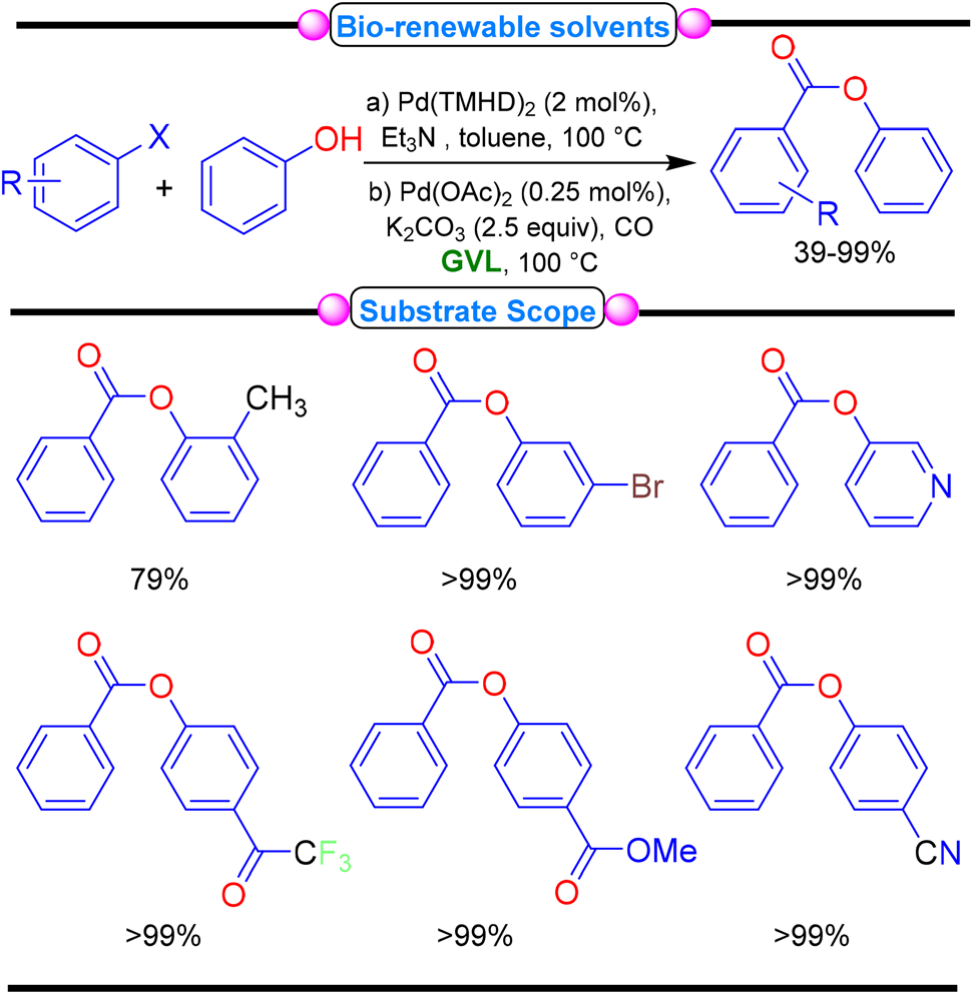
Alkoxy and aryloxycarbonylation of arylhalogenides.

A direct path to the synthesis of aromatic esters is provided by the transformation of aryl halides with alcohols and phenols as *O*-nucleophiles. These structures are frequently used as building blocks for physiologically active substances, such as anti-inflammatory, antiseptic, and photosensitising agents.^231–233^ Tambade *et al.* revealed that under more tolerant operating conditions, the structurally well-defined transition metal complex palladium bis(2,2,6,6-tetramethyl-3,5-heptanedionate) is an effective catalyst for alkoxycarbonylation and aminocarbonylation processes (Scheme 39a).^234^ By adapting the phosphine-free Pd catalyst systems, Tukacs *et al.* effectively replaced fossil-based solvents and triethylamine, a toxic and volatile organic base, with GVL as a bio-based, non-volatile solvent, and K_2_CO_3_, as an inorganic base, for the carbonylation-coupling between phenols and aryl iodides (Scheme 39b).^235^ This study examined the impact of several reaction parameters, including catalyst and base loadings, temperature, carbon monoxide pressure, and other solvents, on the efficiency of the carbonylation reaction.

**Scheme 40:**
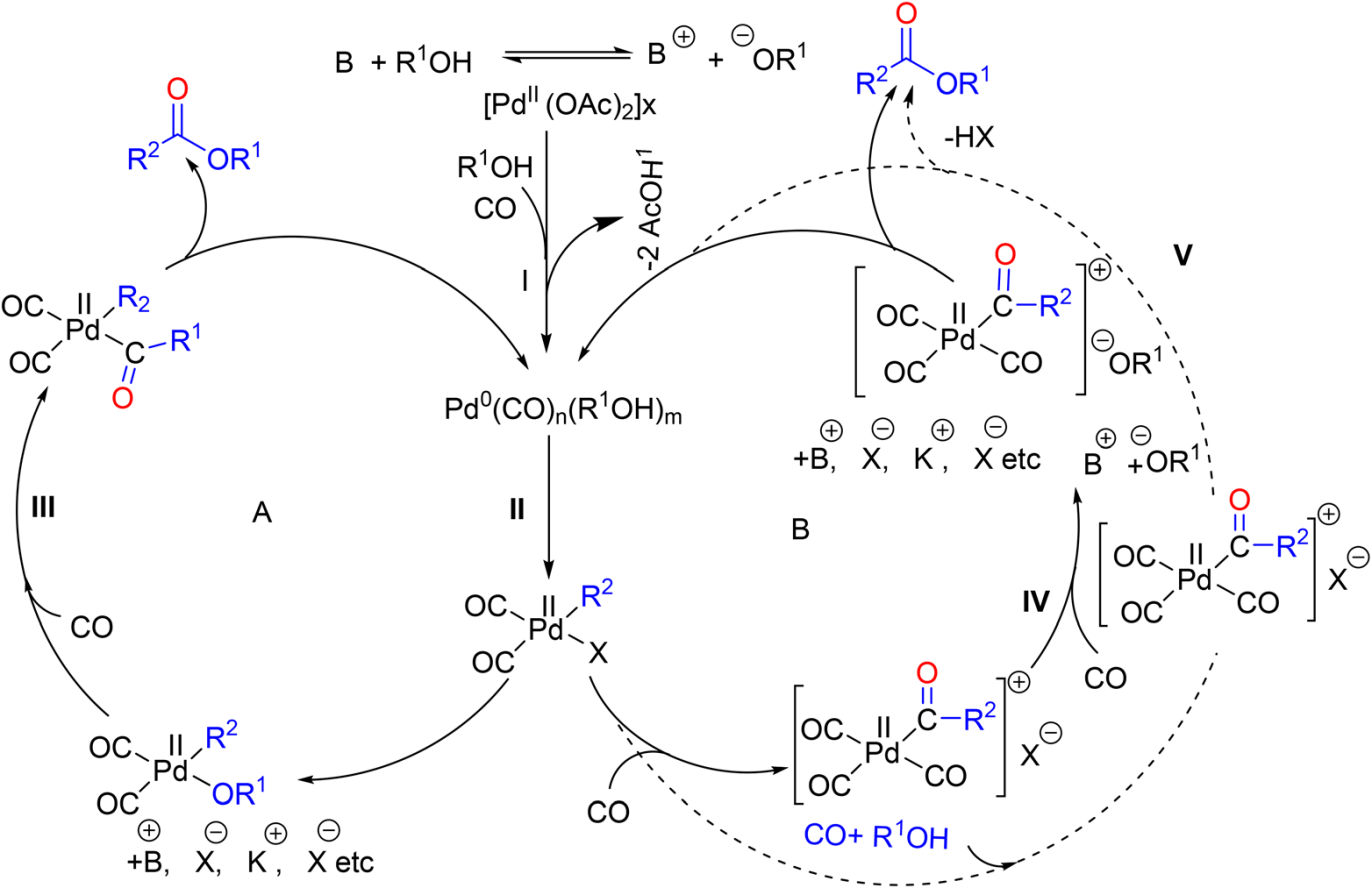
Plausible mechanisms for the carbonylation of aryl halogenides using homogeneous ligand-free Pd-systems.

Investigations were conducted into the effects of *para*-substitution of phenol and the electronic parameters (*σ*) of different aromatic substituents of aryl iodides on the reactivity. For phosphine-modified Pd-catalysts, two main pathways-the acyl route (B) and the alkoxycarbonyl route (A)-have been postulated (Scheme 40). With alcoholysis and phenolysis of the resulting acyl complexes acting as rate-determining steps in path V in Scheme 40, and the acyl-route (B) was often determined to be the effective pathway, particularly in the absence of additional bases. Additionally, it has been shown that method A is significantly delayed in methanol under CO-pressure because coordinated CO prevents –OMe from coordinating to produce a PdC(O)OMe (Pd–carbalkoxy) group utilizing Pd–phosphine systems (from added NaOMe). However, the oxidative addition of aryl iodide (step II in Scheme 40) could be thought of as the rate-determining step in the presence of an additional base. Similar to the interaction of an acyl group with an alkoxide nucleophile, it is also known that CO insertion into a phosphine-ligated Pd–alkyl (step IV) or into a phosphine-ligated Pd–OMe (step III) bond is frequently undetectably quick. The enhanced polarization of the *ipso*-C–I bond might have accelerated the pace of the critical oxidative addition step of R–I (step II in Scheme 40) competition.

**Scheme 41:**
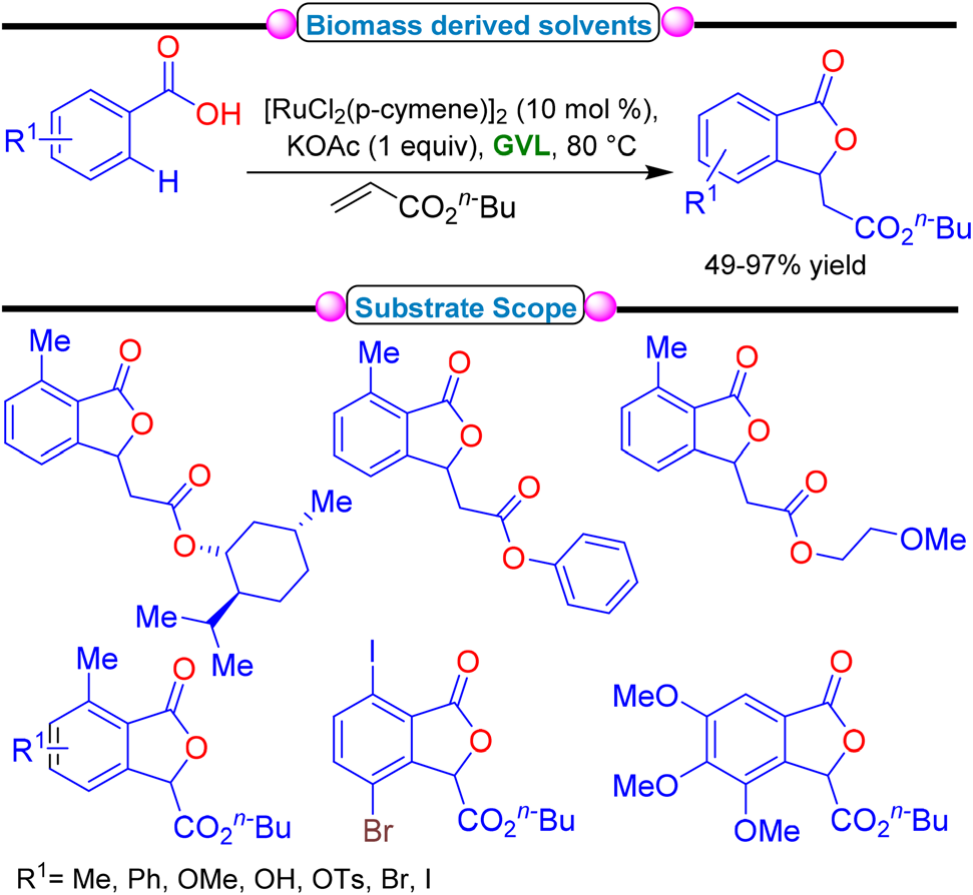
Ruthenium-catalysed arylation of C–H bonds using GVL as a solvent.

Recently Bechtoldt *et al.* demonstrated a sustainable synthesis of dihydroisobenzofuran from 2-benzoic acid 1 and alkene 2 using ruthenium(ii) biscarboxylate-catalysed C–H functionalization using GVL a biomass derived solvent as the reaction media (Scheme 41).^236^ The Ru(ii) catalyst delivered the desired product 3 in 97% yield. The oxidase catalysis was optimized by a wide range of substrates and went smoothly when oxygen was used as the oxidant. The fact that H_2_O is the only byproduct of this C–H activation approach reflects its overall environmental friendliness.

**Scheme 42:**
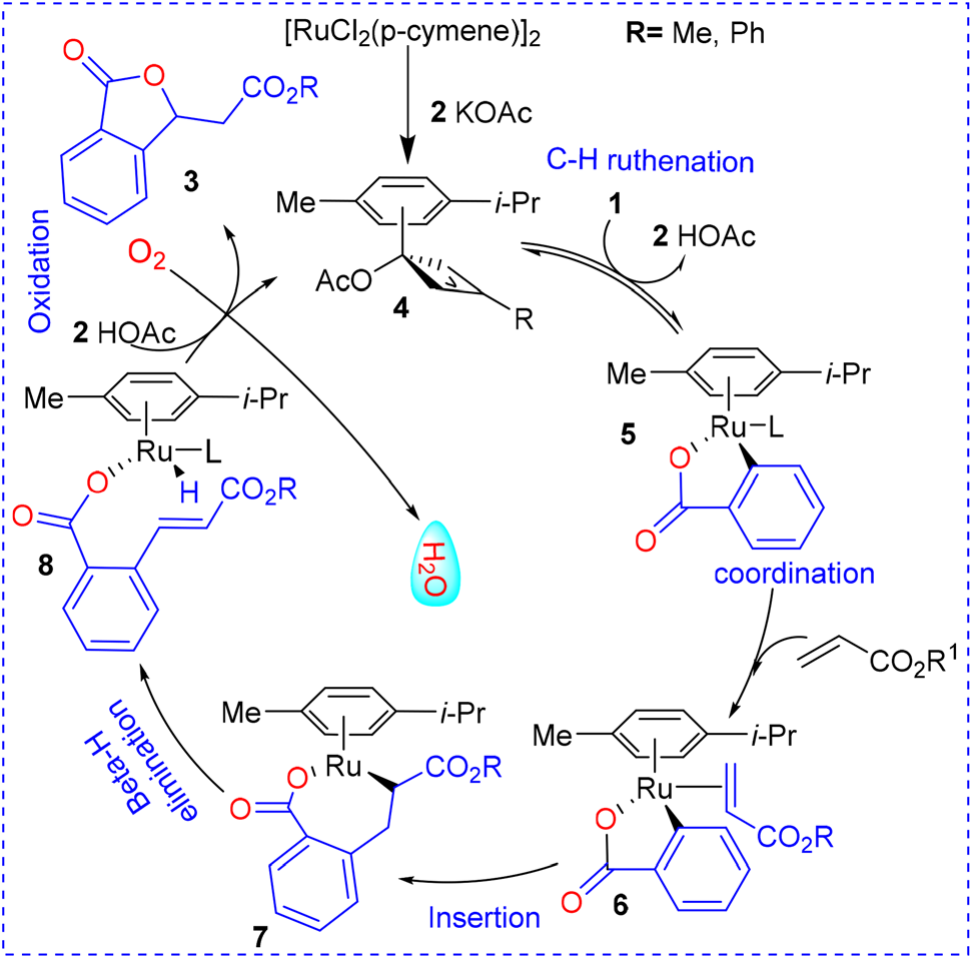
Plausible reaction mechanism for the ruthenium oxidase.

According to the proposed mechanism (Scheme 42), metallacycle 5 is formed by the simple base-assisted internal electrophilic-type substitution (BIES) type C–H ruthenation of benzoic acid 1 by the *in situ* produced ruthenium(ii) biscarboxylate complex 4. Afterwards, complex 7 is produced *via* the coordination of the alkene 2, its insertion, and a subsequent isomerization. Ultimately, β-hydride elimination and intramolecular oxa-Michael addition provide the desired product 3, and a two-electron oxidation step regenerates the catalytically active species 4. It is noteworthy that H_2_O is the sole stoichiometric byproduct of the aerobic C–H alkenylation.

**Scheme 43:**
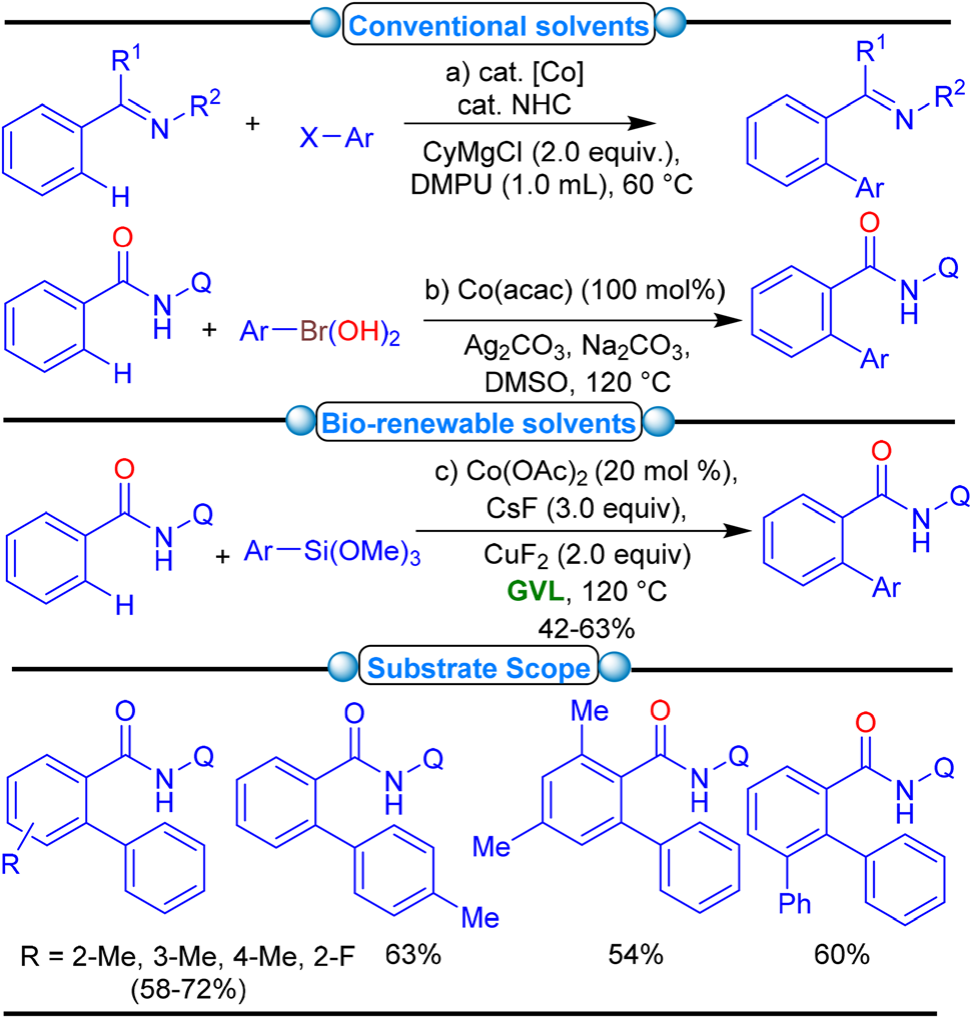
Cobalt-catalysed arylation of C–H bonds using 2-MeTHF as a solvent.

The distinctive reactivity repertoire and low cost of cobalt-catalysed C–H activation becomes a point of discussion for the synthesis of biphenyl moieties by the coupling of organosiloxanes benzene/organoboranes with a selective *ortho*-bond C–H activation of oxime moieties. Despite great advances, the tolerance of synthetically useful functional groups was significantly reduced because stoichiometric concentrations of highly reactive Grignard reagents were needed (Scheme 43a).^237^ Later developments required stoichiometric quantities of cobalt, although they were represented by C–H arylations with nucleophilic boronic acids in DMSO as a reaction medium (Scheme 43b).^238^ Bu *et al.* used organosiloxanes and cobalt catalysis to carry out position-specific Hiyama-type C–H arylations of benzamides. The use of cosolvent obtained from biomass and the ease of C(sp^2^)–H and difficult C(sp^3^)–H arylations could be accomplished (Scheme 43c).^239^ The C–H arylation had a wide range of substrates, including difficult C(sp^2^)–H activation by using GVL as a reaction medium obtained from biomass.

**Scheme 44:**
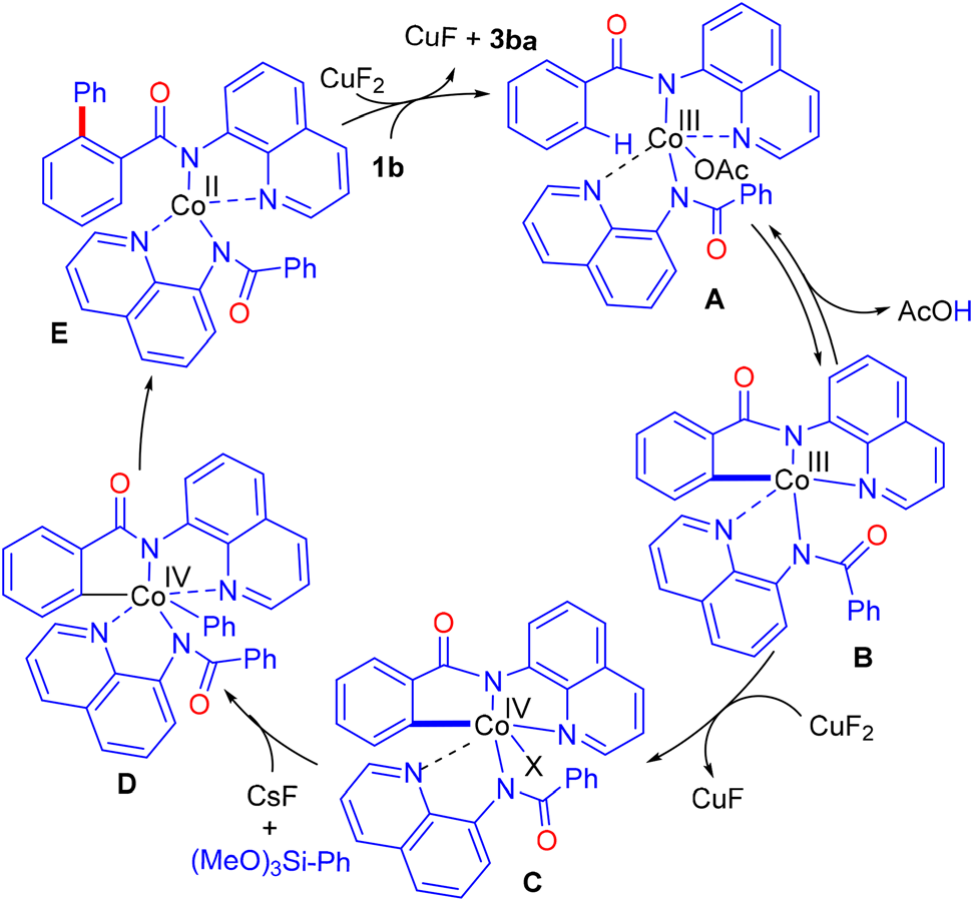
Plausible catalytic pathway of the cobalt-catalysed arylation of C–H bonds.

A feasible catalytic cycle was implemented to be initiated by rapid carboxylate-assisted C–H activation at cobalt(iii) catalyst A (Scheme 44) based on literature surveys. Next, the resulting cobaltacycle C was proposed to undergo a transmetalation, which resulted in the cobalt(iv)–aryl intermediate D. Lastly, it was suggested that oxidation-induced reductive elimination at cobalt(iv) yielded product 3ba, whereas reoxidation produced the catalytically competent cobalt(iii) complex again.

**Scheme 45:**
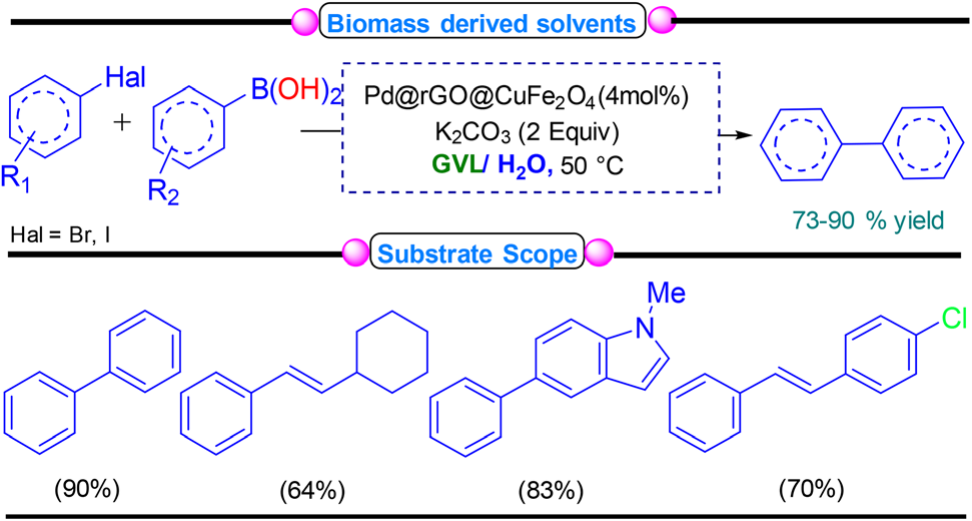
Pd@rGO–CuFe_2_O_4_ catalyst for the synthesis of biphenyls using GVL.

In contrast to the above, our group (Scheme 45) have recently developed a ligand-free heterogeneous catalyst consisting of Pd-decorated reduced graphene oxide–copper ferrite magnetic nanoparticle (Pd@rGO–CuFe_2_O_4_) for the synthesis of biphenyls and other chemical moieties.^240^ This unique catalyst does not require a linker to hold the metal ion, and offers advantages over previous approaches. This catalyst utilizes a very low amount of Pd (0.00047 mole per cent per mole of haloarenes). It demonstrates an efficient performance in Suzuki–Miyaura coupling reaction and the oxidation of nitriles into amides, following a green approach using a renewable GVL; H_2_O. Moreover, the biomass-derived solvents are employed, which further enhances the sustainability of this process. The catalyst can be easily separated through the decantation process from the reaction medium without the need for external forces or assistance. This catalyst produces a yield ranging from 64–98% with a variety of aliphatic, aromatic and heteroaromatic substrates.

**Scheme 46:**
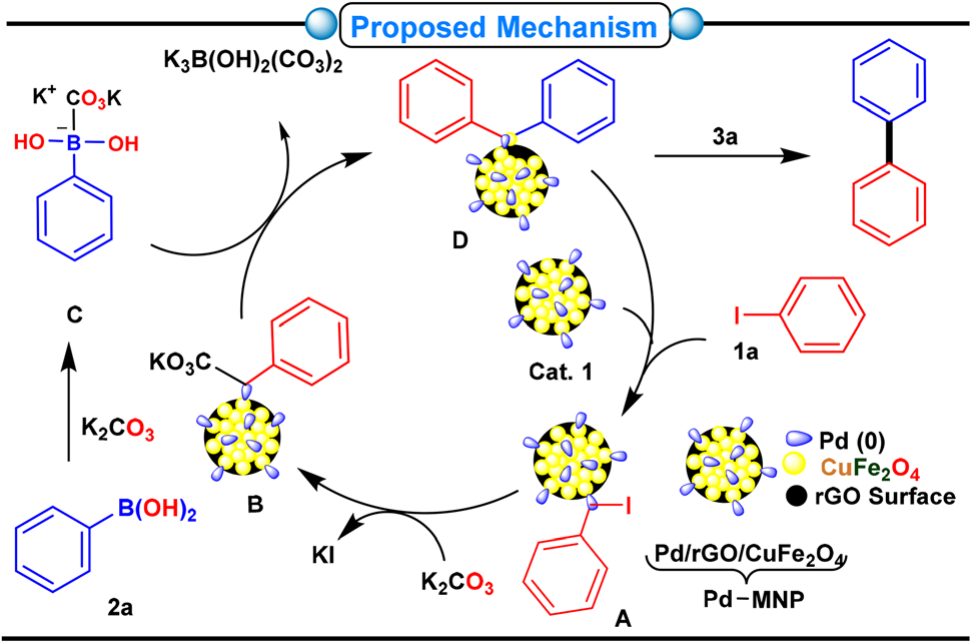
Plausible mechanistic pathway for the synthesis of biphenyls.

The mechanism of the developed coupling reaction using the Pd@rGO–CuFe_2_O_4_ MNPs as a nanocatalyst (Scheme 46). The mechanism initiates with the oxidative addition of aryl halide 1a to Pd(0) MNPs (cat. 1) to form an organo Pd(ii) species A, and is the rate-determining step. The following step involves the utilization of the first equivalent of base (K_2_CO_3_) to form an intermediate B. On the other side, the aryl boronic acid 2a may participate in the reaction with a second equivalent of base (K_2_CO_3_) to generate an ionic intermediate C. The intermediates B and C then undergo a transmetalation process to form the complex D, in which the palladium is in +2 oxidation state. The last step involves the reductive elimination process to release the final biphenyl molecule 3a and regenerate the Pd(0) species, which can be adsorbed back after the completion of the catalytic cycle.

**Scheme 47:**
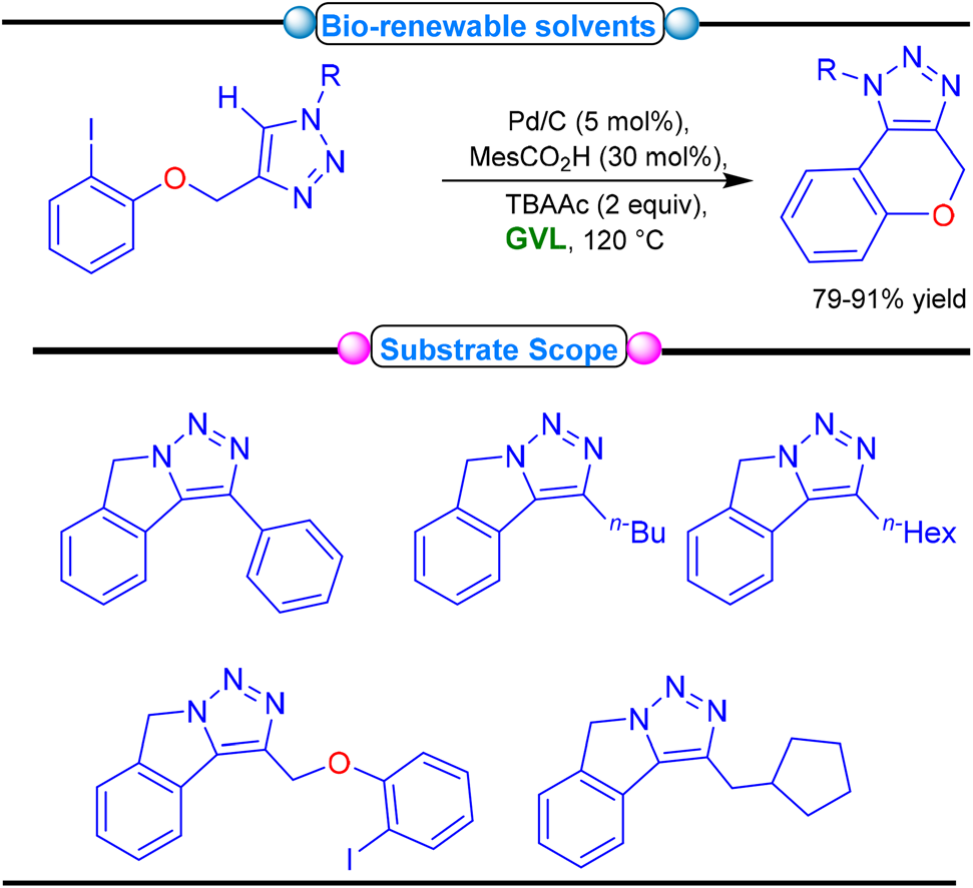
Synthesis of 1,2,3-triazoles using GVL as the reaction medium.

A chemically effective and waste-minimized method for the C–H functionalization of 1,2,3-triazoles in continuous flow regime has been disclosed by Ferlin *et al.* (Scheme 47).^241^ The necessary products could be obtained by using the heterogeneous catalyst (Pd/C) in conjunction with a soluble organic base and GVL as the reaction medium. The optimized reaction condition gave the desired products in excellent yields and hourly productivity. The use of flow chemistry was addressed here in order to enhance the catalyst longevity and recyclability in addition to that of waste minimization. Several brominated and iodinated substrates were employed for this coupling reaction, where higher yield was obtained in iodinated substrates. Catalyst leaching marginally reduced the yield to 83% in the 5th recycle from 87% in the 1st and 2nd recycle.

**Scheme 48:**
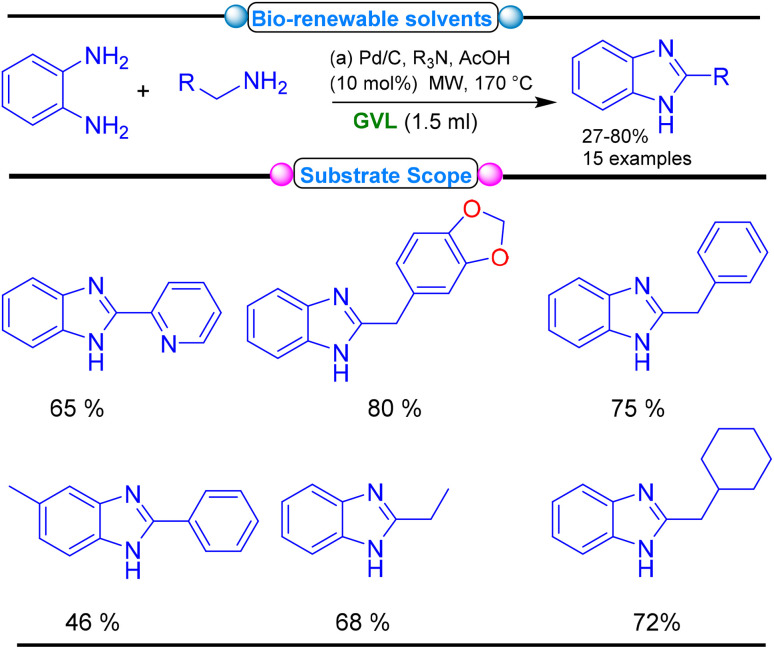
Microwave-assisted synthesis of benzimidazoles.

A regioselective formation of benzimidazoles in GVL was accomplished by Petricci *et al. via* a microwave assisted synthesis (Scheme 48). The transformation was carried out using Pd/C as a reaction accelerator, this one pot synthesis involves the use of bio-renewable solvent GVL, microwave as an external energy and Pd/C as heterogeneous catalysts was characterised for the first time demonstrating its stability and compatibility.^242^ In the hydrogen transfer Pd/C-mediated synthesis of benzimidazoles, the usage of GVL is compatible with aliphatic as well as aromatic amines with a moderate to excellent yield of 27–80%.

**Scheme 49:**
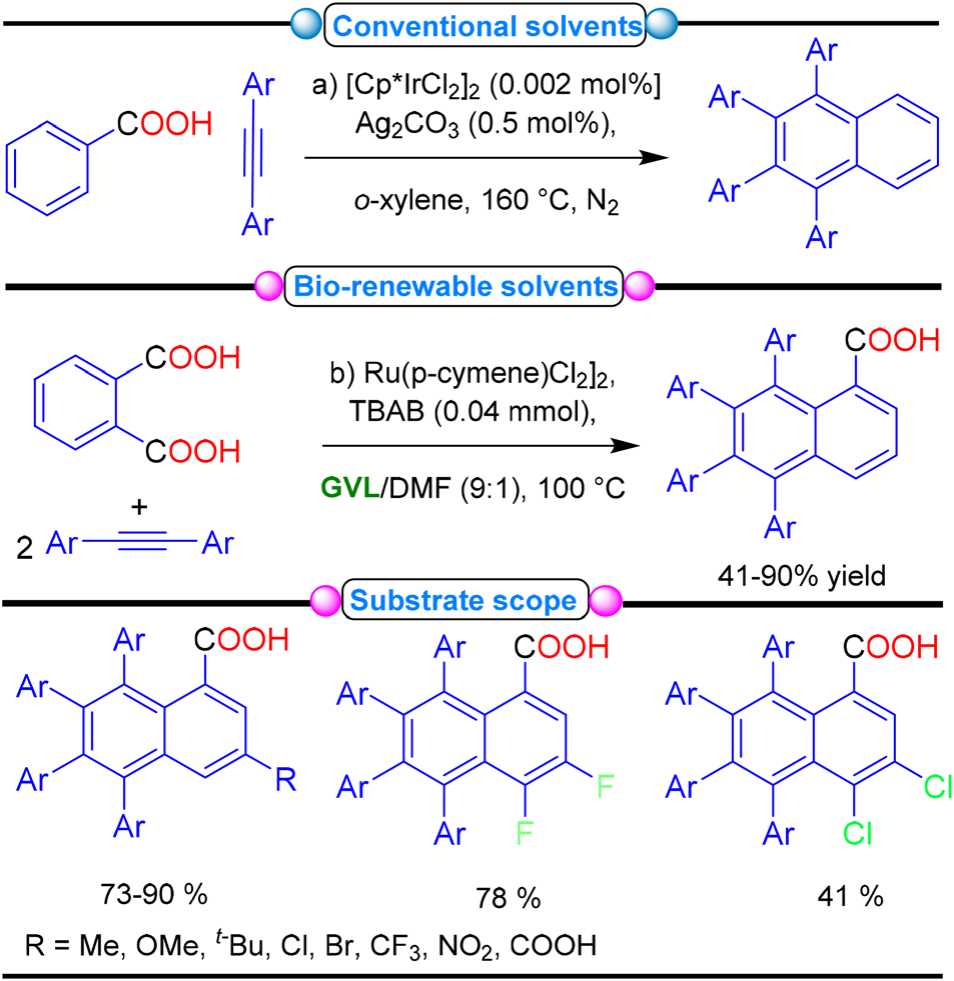
Coupling modes of alkynes and aromatic acids in GVL.

The oxidative coupling of internal alkynes with benzoic acids was carried out by Ueura *et al.* in 2007 using [Cp*RhCl_2_]_2_ as the catalyst and Ag_2_CO_3_ as an oxidant to synthesise naphthalene derivatives in *o*-xylene medium (Scheme 49a).^243^ Later Chen *et al.* in 2019 developed an effective [2 + 2 + 2] benzannulation of multisubstituted 1-naphthoic acids with two equivalents of alkynes *via* Ru-catalysed C–H activation (Scheme 49b).^244^ Using ambient oxygen as the only oxidant with high atom/step economies, and free carboxyl group, the reaction was used to transform the products into a variety of polycyclic compounds. In contrast to the preceding reactions which produced trace byproducts, this reaction allows air to function as the only oxidant and GVL, improving atom economy, sustainability, and functional group tolerance.^245–247^

**Scheme 50:**
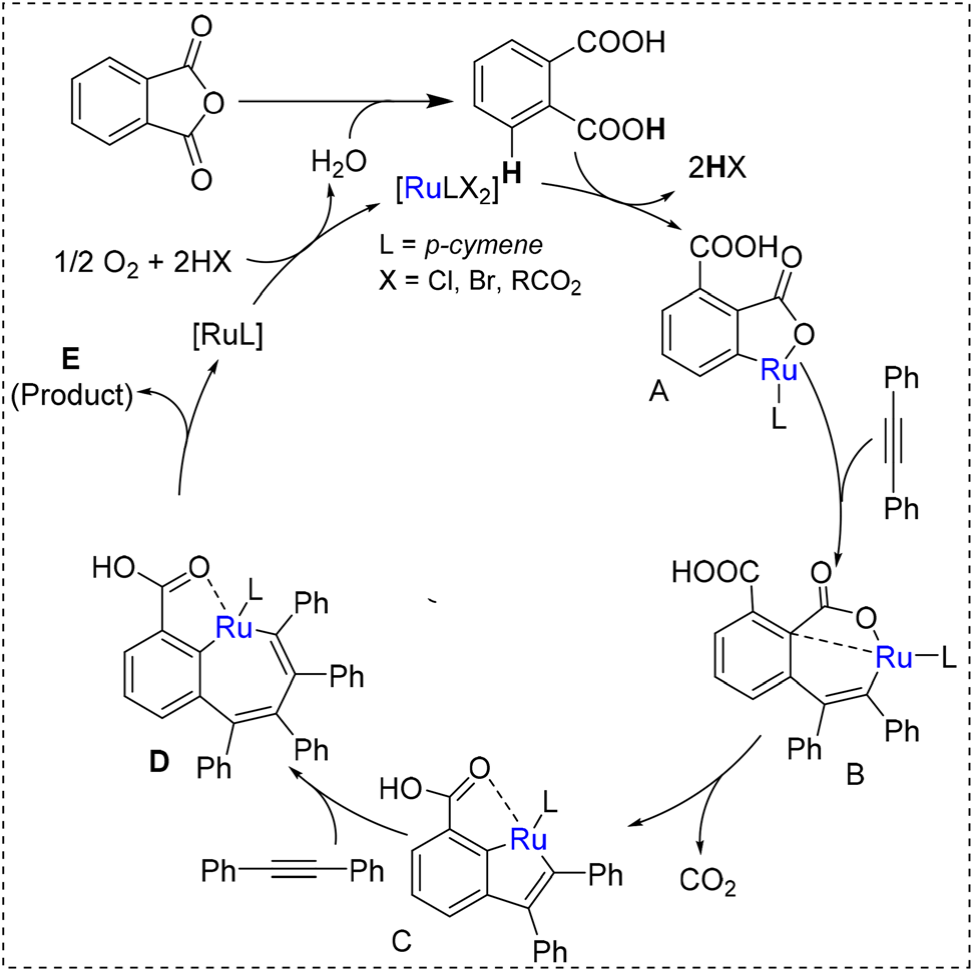
Proposed reaction mechanism of Ru-catalysed coupling between aromatic acids and alkynes.

In the proposed reaction mechanism (Scheme 50), initially, the TBAB and solvent react with [Ru(*p*-cymene) Cl_2_]_2_, forming an active species. The activated species reacts with benzoic acid to generate intermediate A by a ligand exchange and C–H bond cleavage. The next step involves the alkyne insertion to produce intermediate B, which is then undergo decarboxylation to produce intermediate C with a little fragile coordination. The remaining carboxyl group shows secondary interaction with Ru to stabilize the intermediate C. The second molecule of alkyne undergoes insertion on the intermediate C to produce intermediate D, which undergoes reductive elimination to give the product E and leaves Ru(0) species aside. To complete the catalytic cycle the Ru(0) species can be oxidized with atmospheric oxygen to regenerate the active Ru(ii) catalyst.

**Scheme 51:**
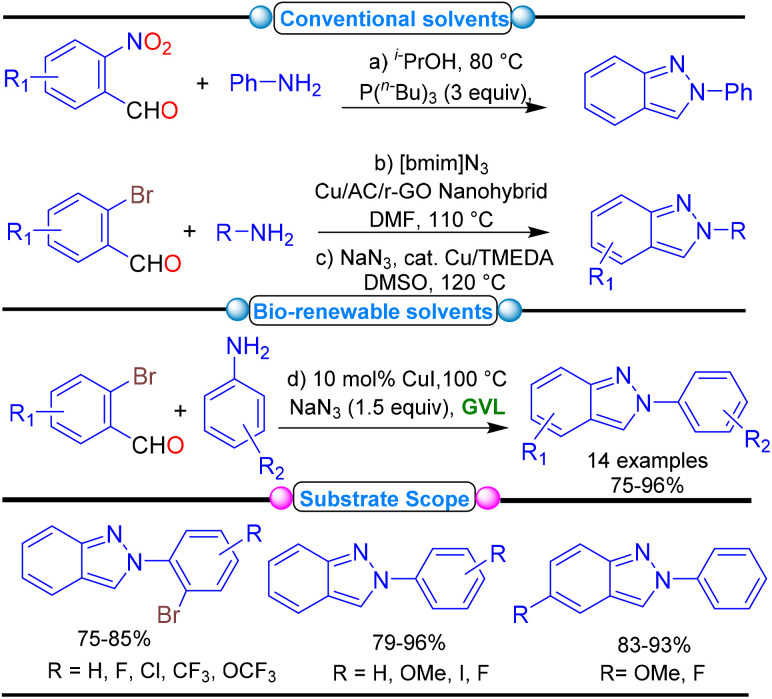
Synthesis of 2-aryl-2*H*-indazoles using various amines in GVL.

Malakar *et al.* developed a ligand and base free Cu(i)-catalysed multicomponent reaction toward the synthesis of 2-aryl-2*H*-indazoles involving 2-bromobenzaldehydes, arylamines, and sodium azide under the influence of GVL as biomass-derived solvent (Scheme 51d).^248^ The discovered method yielded good to outstanding yields of 2-aryl-2*H*-indazole derivatives when a succession of functional groups was implanted on both aldehyde and amine components. Conversely, groups and substituents lacking electrons that were incorporated into aromatic rings in either mono- or disubstituted patterns demonstrated a rather low yield of the related products. This transformation helped to overcome challenges caused by using hazardous solvents like i-PrOH, DMF, DMSO (Scheme 51a–c).^249–251^

**Scheme 52:**
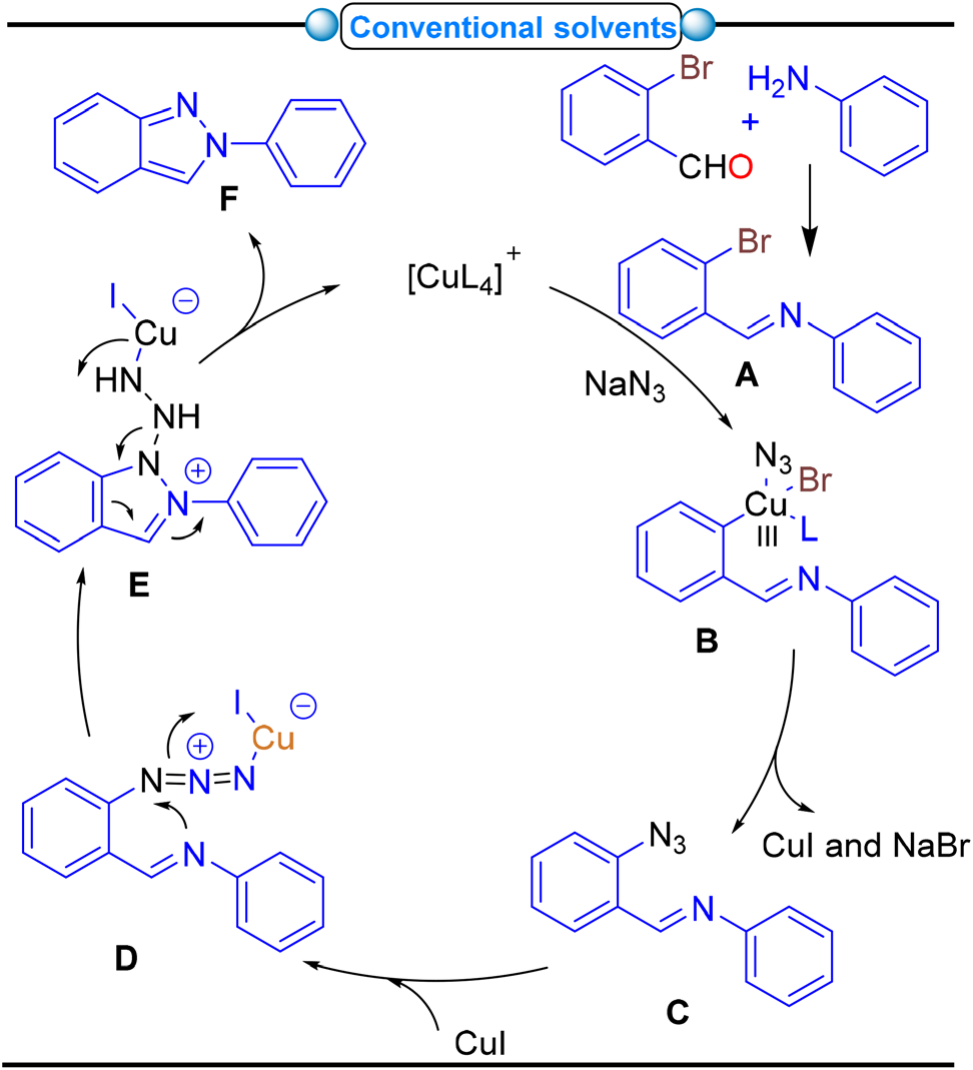
Plausible mechanism for the Cu(i)-catalysed multicomponent reaction in GVL.

The mechanism was proposed by the interaction between substrates produces the imine molecule A (Scheme 52). The aryl azide molecule C is then produced by the [CuL_4_]-catalysed *N*-arylation reaction of moiety A with sodium azide (NaN_3_), and oxidative addition intermediate B is then formed. The complex formation of Cu(i) with GVL to improve the stability of the active catalyst and that promotes the efficacy of the cross-coupling reaction. The intermediate five-membered N-heterocycle E, which is generated by the release of an active Cu(i)-catalyst and the extrusion of molecular nitrogen, may then be the consequence of the cyclization of Cu(i)-azide complex D utilizing N–N bond formation, yielding the anticipated product F.

**Scheme 53:**
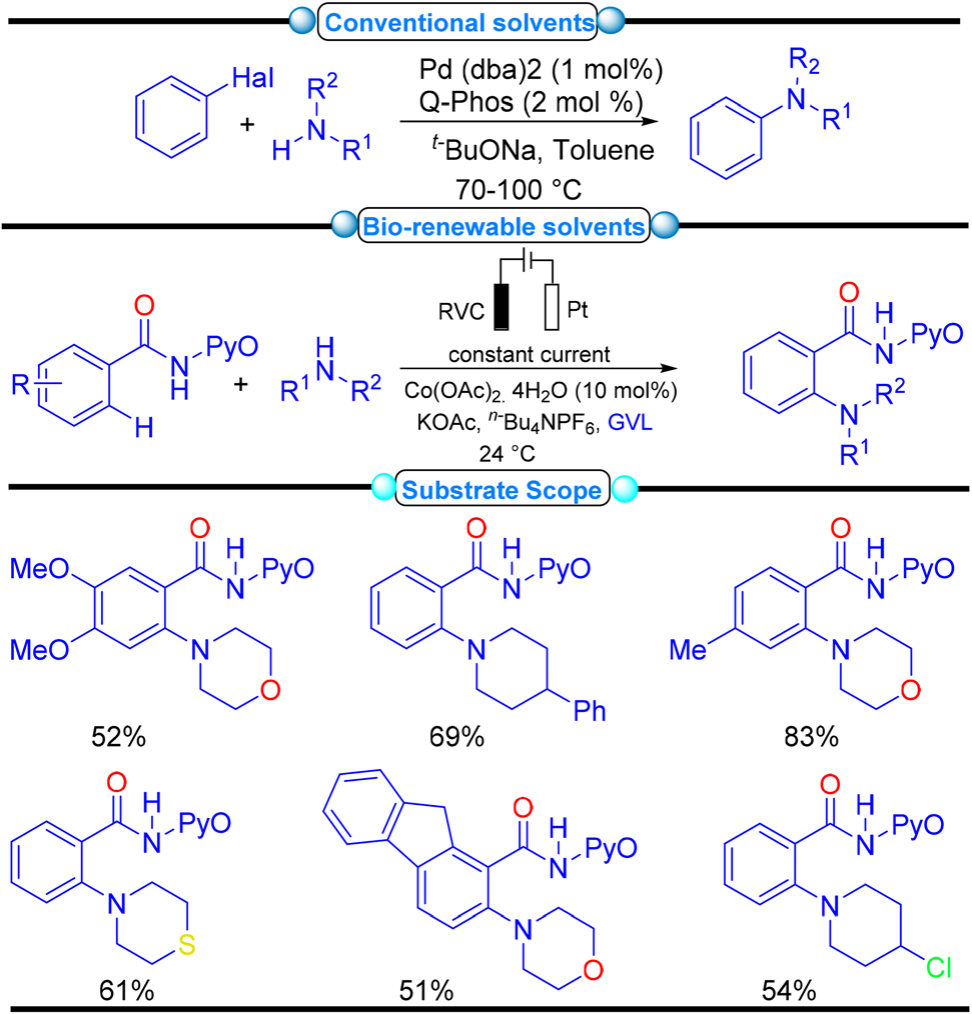
Electrochemical C–H amination of arenes in GVL.

Ackermann *et al.*^252^ (Scheme 53) have designed electrochemical C–H amination of anilines with pre-functionalised aryl-electrophiles that was probed by cyclic voltammetry analysis. This transformation was carried out by using cobalt catalyst in the absence of oxidant and is found to be the first organometallic C–H amination of amides that release H_2_ gas the sole byproduct. The cross-dehydrogenative C–N coupling was solely carried out in the absence of oxidant and by employing electricity as a green oxidant at a temperature of 40 °C. This amination reaction was optimised using a range of synthetic and biobased solvents, and GVL was found to be the best for this transformation. This base-mediated process gives support to the cobalt catalyst by a single electron oxidation with a voltage of (*V*_SCE_ = 1.05), and this transformation produces a moderate to excellent yield of 51–81%. In 2008 the same transformation was carried by Hartwig *et al.* in toluene at 100 °C.^253^

**Scheme 54:**
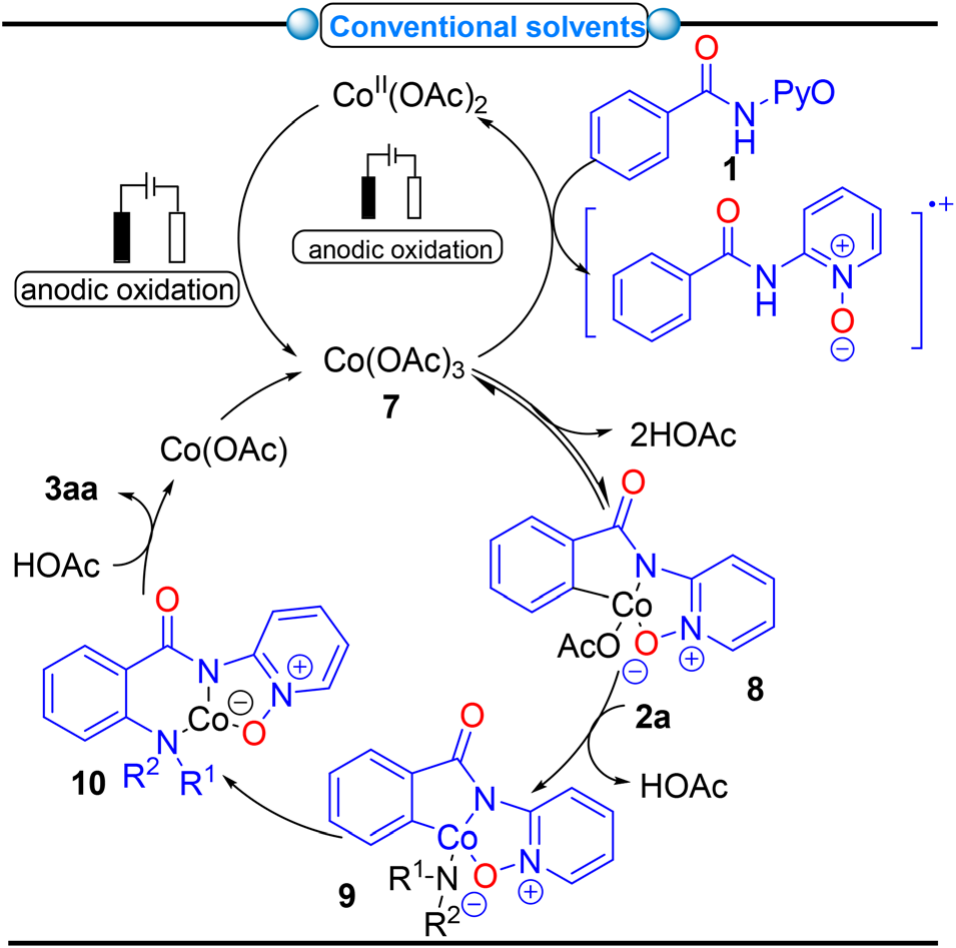
Proposed catalytic cycle.

The reaction mechanism was proposed as the anodic oxidation of Co(ii) to an electrochemically active Co(iii) species (7). In the next step, the C–H activation of carboxylate-assisted species provided intermediate 8, followed by salt metathesis in intermediate 8 and reductive elimination in species 9, releasing the corresponding product and Co(i) species. This Co(i) species underwent anodic oxidation, which again generated the intermediate 7, back to the catalytic cycle (Scheme 54).

**Scheme 55:**
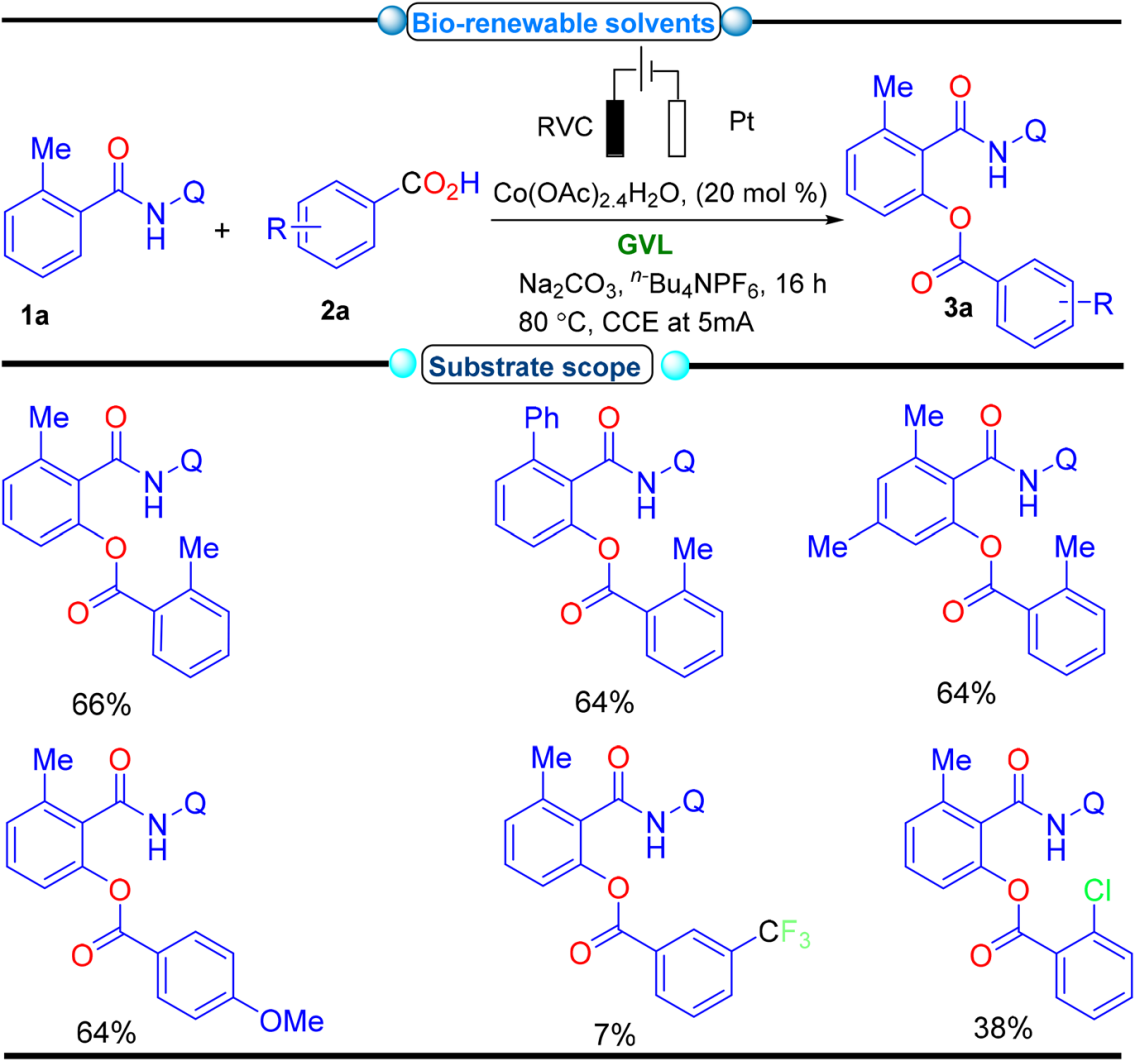
Oxidative electrocatalytic C–H activation.

Very recently, the Ackermann group have also demonstrated that cobalt-catalysed C–H alkylation reaction, which can be achieved under mild reaction conditions at a temperature of 80 °C using Na_2_CO_3_ as a base (Scheme 55).^254^ A chelation-assisted C–H arylation of benzoquinone-amide with carboxylic acid derivatives under electrochemical cobalt catalysis by employing biomass-derived solvent GVL as a reaction medium, enabled the formation of arylated products in excellent yield, with a broad range of substrate scope. ^*n*^Bu_4_NPF_6_ was added as a conducting salt to overcome the low conductivity.

**Scheme 56:**
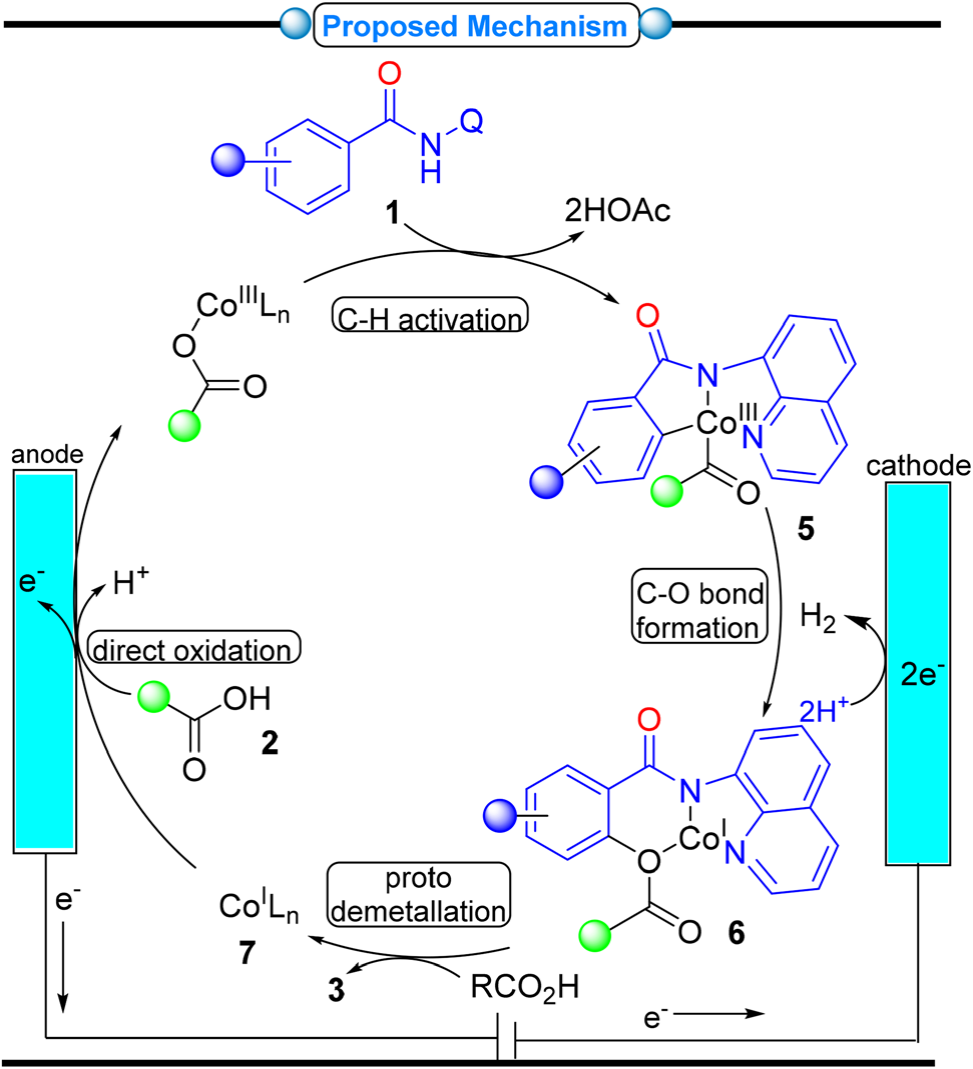
Cobalt-catalysed electrochemical C–H oxygenation in GVL.

Amide substrate 1a gave rise to an irreversible oxidation at 1.46 V *vs.* Fc^+/0^ (blue), while the acid 2a did not show any relevant oxidation in MeOH. A plausible catalytic cycle has been proposed based on previous studies (Scheme 56). The mechanism was initiated by the electrochemical formation of Co(iii) species *via* anodic oxidation. The C–H activation of carboxylated active species produced the cyclometalated species 5. Metathesis with carboxylic acid 2 in the subsequent step led to the Co(iii) intermediate 6.^255^ Finally, the reductive elimination of 6 delivers the product 3a and generates Co(i) species 7. Finally, the anodic oxidation of 7 regenerates the catalytically active Co(iii) complex 4.

### 4.3. Cyclopentyl methyl ether (CPME)

**Scheme 57:**
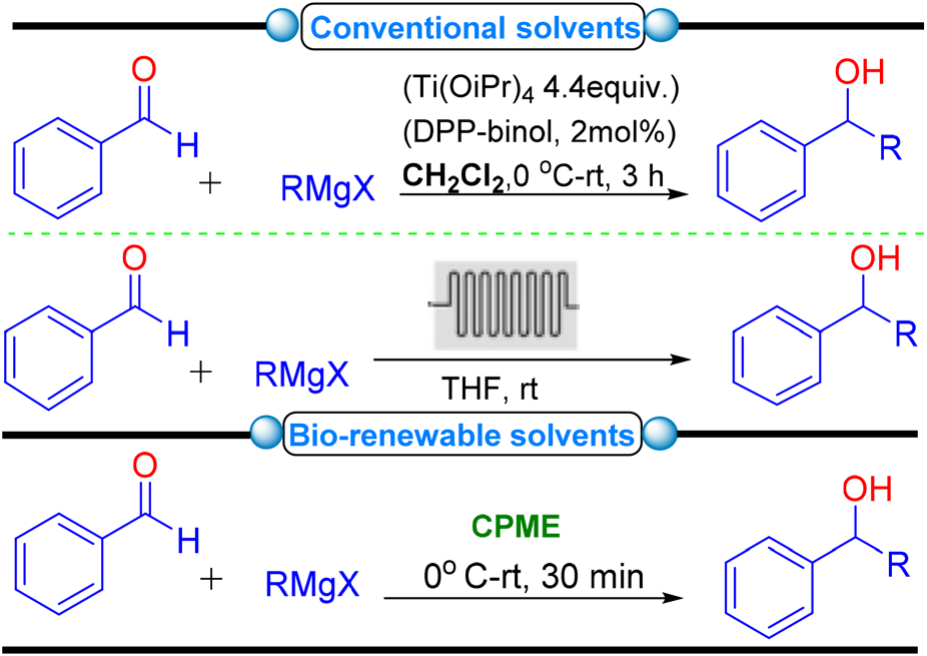
Synthesis of tertiary alcohols by the Grignard reagent.

Harada *et al.* in 2008 demonstrated the binol–titanium(iv) derivative by using excess titanium tetraisopropoxide for the asymmetric alkylation of aldehydes with Grignard reagents in CH_2_Cl_2_ (Scheme 57a).^256^ A similar protocol was reported by A. Rencurosi and group in 2010 using a continuous-flow apparatus in THF solvent (Scheme 57b).^257^ Masuyama *et al.* demonstrated the utility of CPME in magnesium-mediated selective 1,2-additions of Grignard reagents and aldehydes to produce a variety of secondary alcohols (Scheme 57c).^258^ The magnesium metal is activated by diisobutylaluminium hydride, leading to the synthesis of several homogeneous and heterogeneous Grignard reagents in CPME solution. Notably, only 1,2-addition products were detected by ^1^H-NMR, and the reactions were conducted at 0 °C to room temperature, making this an attractively simple method.

The CPME used during the reaction is recycled without affecting the reaction yield and reused in the next run. Analogous reactions conducted in 2-MeTHF (1.02 M) and THF (0.97 M) solvents require a higher concentration, as determined by titration, than in CPME (0.81 M). CPME used during reaction is recycled without affecting the reaction yield and reused for the next run. Analogous reactions conducted in 2-MeTHF (1.02 M) and THF (0.97 M) solvents require a higher concentration detected by titration compared to CPME (0.81 M).

**Table:** 

Solvent	Yield
CPME	87
THF	92
2-MeTHF	91

**Scheme 58:**
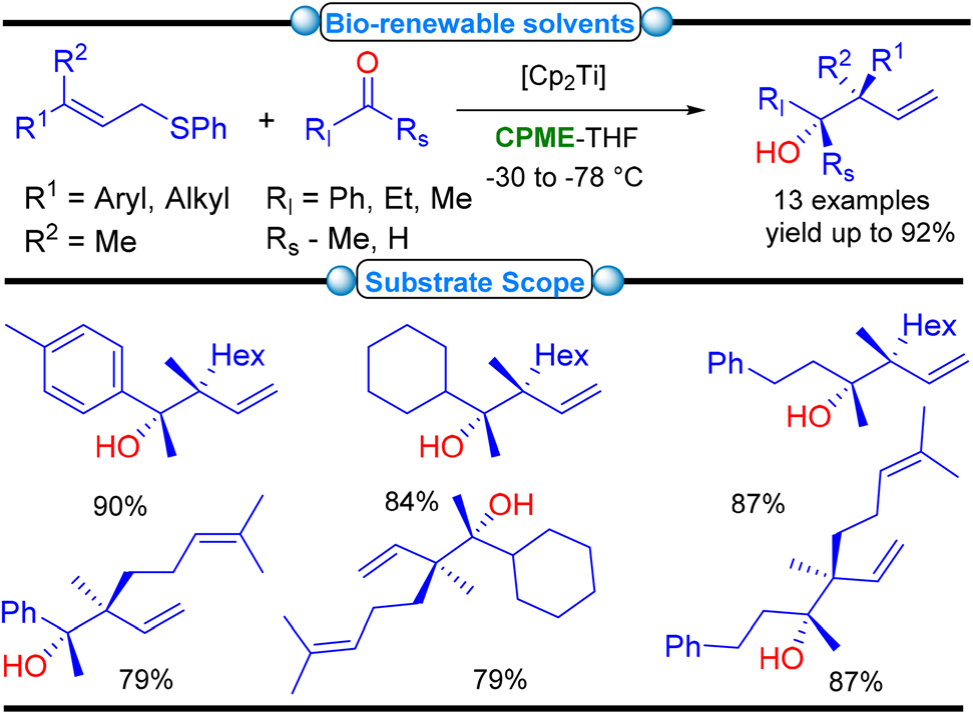
Stereospecific reaction of allyl sulfides with ketones.

The more challenging task of constructing acyclic systems with two adjacent stereogenic centres has been successfully achieved by Knochel *et al.* and Marek *et al.*,^259,260^ Takeda *et al.* in 2012 developed a stereoselective addition of γ,γ-disubstituted allyl titanocenes to ketones to generate a stereogenic centre that is adjacent to each other.^261^ The allyl titanocenes are formed by the desulfurizative titanation of 3-phenyl-2-butenyl phenyl sulfide [both (*E*)- and (*Z*)] with the titanocene(ii)–1-butene (v) complex in CPME–THF solvent at −30 °C with a retention in configuration, which further reacts with the ketone at −110 °C to generate tertiary homoallylic alcohols. To make this process sustainable and greener, CPME, in conjunction with THF, has been used as a reaction carrier (Scheme 58). Under these mild reaction conditions, diverse tertiary alcohols were synthesised in high yields from broad substrates containing both aliphatic and aromatic groups.

**Scheme 59:**
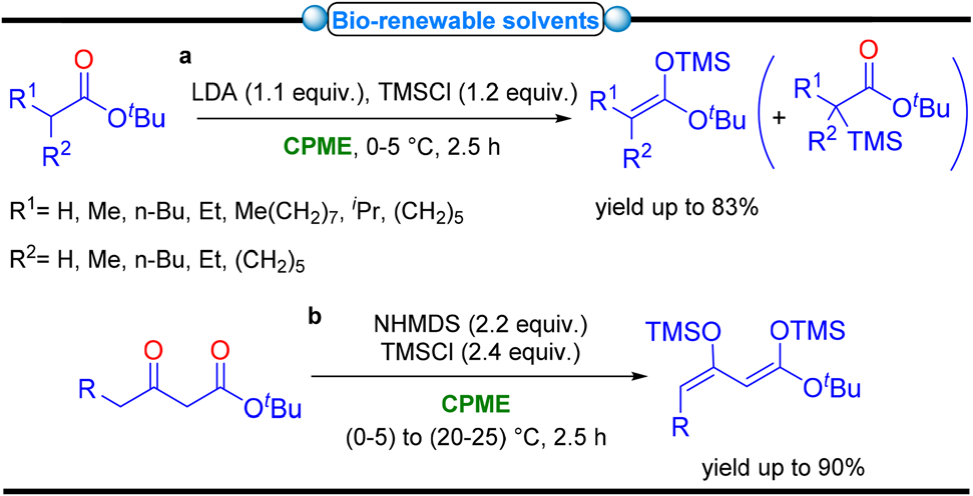
Synthesis of *E*-ketene silyl acetals.

Despite their popularity, ketene silyl acetals (KSAs) have drawbacks, such as the need for very low temperatures and instability in the presence of acids or bases.^262,263^ Inspired by this work, Tanabe *et al.* demonstrated the stereo and regio-selective tosylation of *tert*-butyl esters and *tert*-butyl α-ketoesters to form (*E*)-KSAs, and 1,3-bis(TMS)-KSAs having electron-donating groups by considering TMSCl as a silylating agent (Scheme 59).^264^ Mild reaction conditions and the use of cyclopentyl methyl ether (CPME) as a biomass-derived solvent are promising features of this reported transformation. Using this synthetic protocol, stereocontrolled preparation of highly reactive β-ketoester-derived *tert*-butyl (1*Z*,3*E*)-1,3-bis(TMS) dienol ethers was achieved up to 90% of yield.

**Scheme 60:**
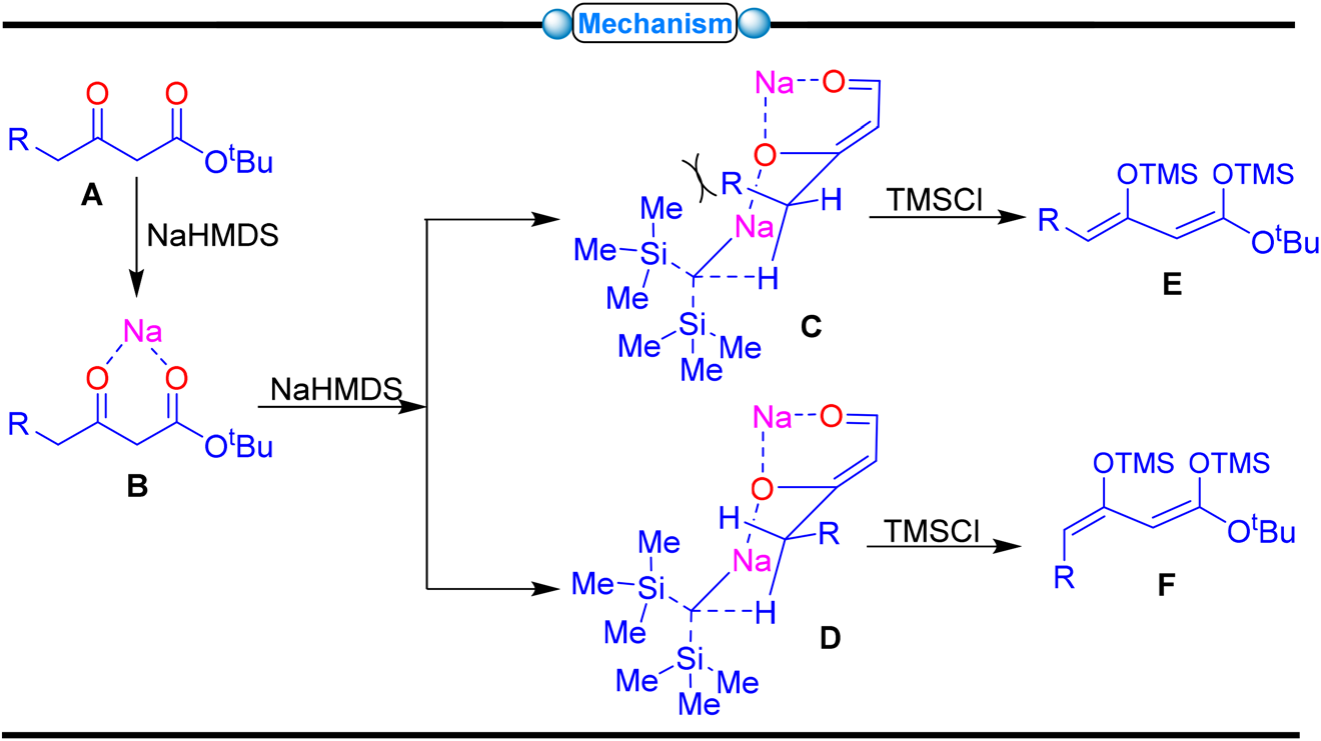
Proposed mechanism of *Z* and *E*-ketene silyl acetals.

The possible catalytic cycle has been discussed by considering *tert*-butyl β-ketoester as a starting material (Scheme 60). Initially, *tert*-butyl β-ketoester is converted to sodium monoenolate which is transformed to disodium dienolate. The E intermediate is formed in order to avoid the steric repulsion between the bulky hexamethyldisilazane group and the R group which is further attacked by TMSCl to form (1*Z*,3*E*)-diastereomer.

**Scheme 61:**
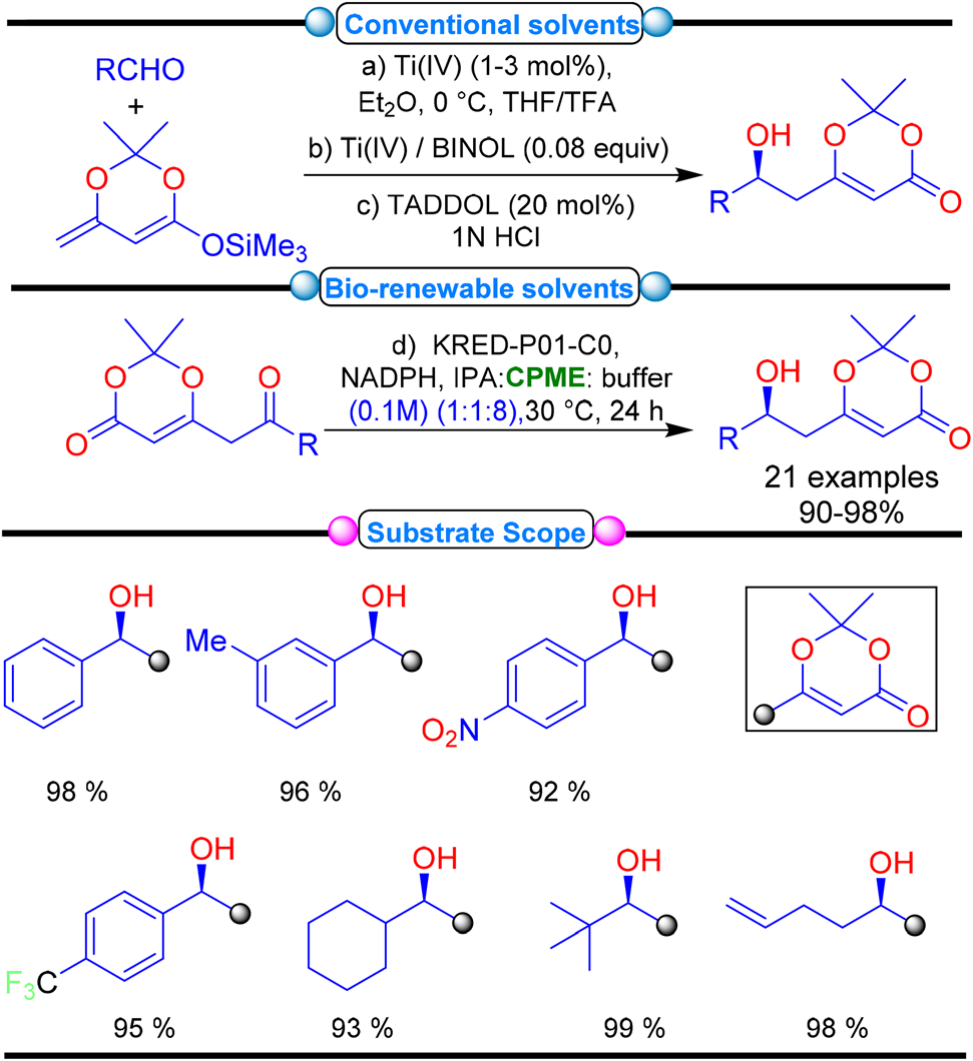
Strategies for the synthesis of β-hydroxy dioxinone.

CPME has also been used as a cosolvent in conjunction with IPA and 0.1 M buffer for the synthesis of some δ-hydroxy-β-keto esters, *syn*- and *anti*-β,δ-diol esters, chiral β-hydroxy dioxinone compounds.^265–268^ For these transformations a number of chiral catalysts, such as Ti(iv)–BINOL complexes (Scheme 61a), Ti(iv)–Schiff base complexes (Scheme 61b), and TADDOL catalysts (Scheme 61c), have shown to be effective.^269–271^ Betori *et al.* have developed a unique biocatalytic method that uses commercially produced keto-reductases for the enantioselective reduction of β-keto dioxinones to sp^3^ carbon of β-hydroxy dioxinones which are adjacent to stereogenic centre (Scheme 61d).^272^ Some of the features that makes this method more prominent than the others includes high enantioselectivity, high yield, and chromatography-free product purification.

**Scheme 62:**
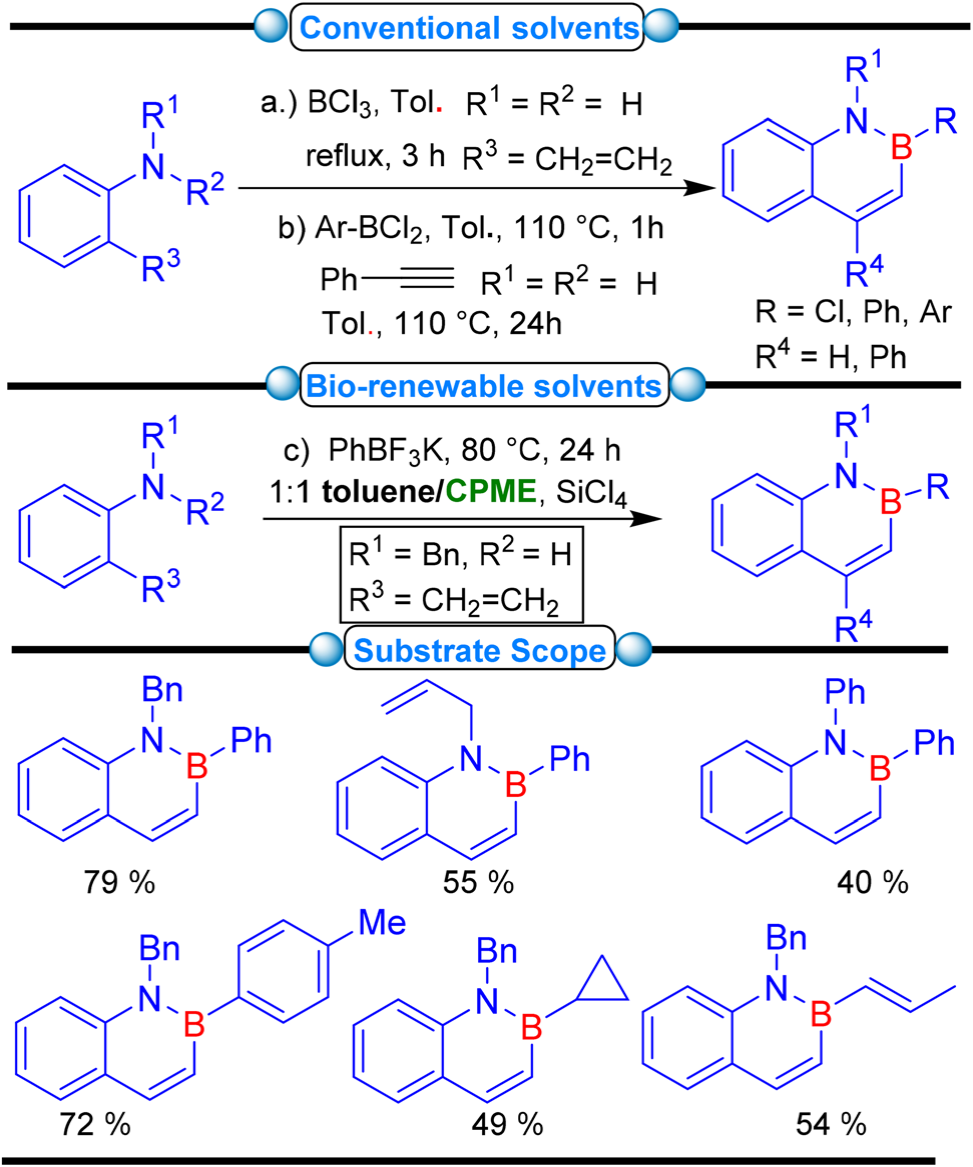
Synthesis and functionalization of 2,1-borazaronaphthalenes.

Dewar and Dietz (1959) reported the first borazine compound, 2-chloro-2,1-borazaronaphthalene, by an annulation reaction of BCl_3_ and 2-aminostyrene under reflux in toluene (Scheme 62a).^273^ Furthermore, a number of articles were reported to overcome this drawbacks which employs synthetic solvents as a reaction medium (Scheme 62b).^274,275^ In 2014, Gary A. Molander reported the combination of CPME and toluene for the synthesis of nitrogen- and boron-substituted borazaronaphthalene. A library of boron- and nitrogen-substituted borazaronaphthalenes were developed by using substituted 2-amino styrenes and a number of heteroaryl-, alkynyl-, aryl-, alkenyl-, and alkyl potassium organotrifluoroborates (Scheme 62c).^276^ This innovative method utilises SiCl_4_ as a catalyst to convert organotrifluoroborates into organodihaloboranes and leads to the formation of functionalized azoborines.

**Scheme 63:**
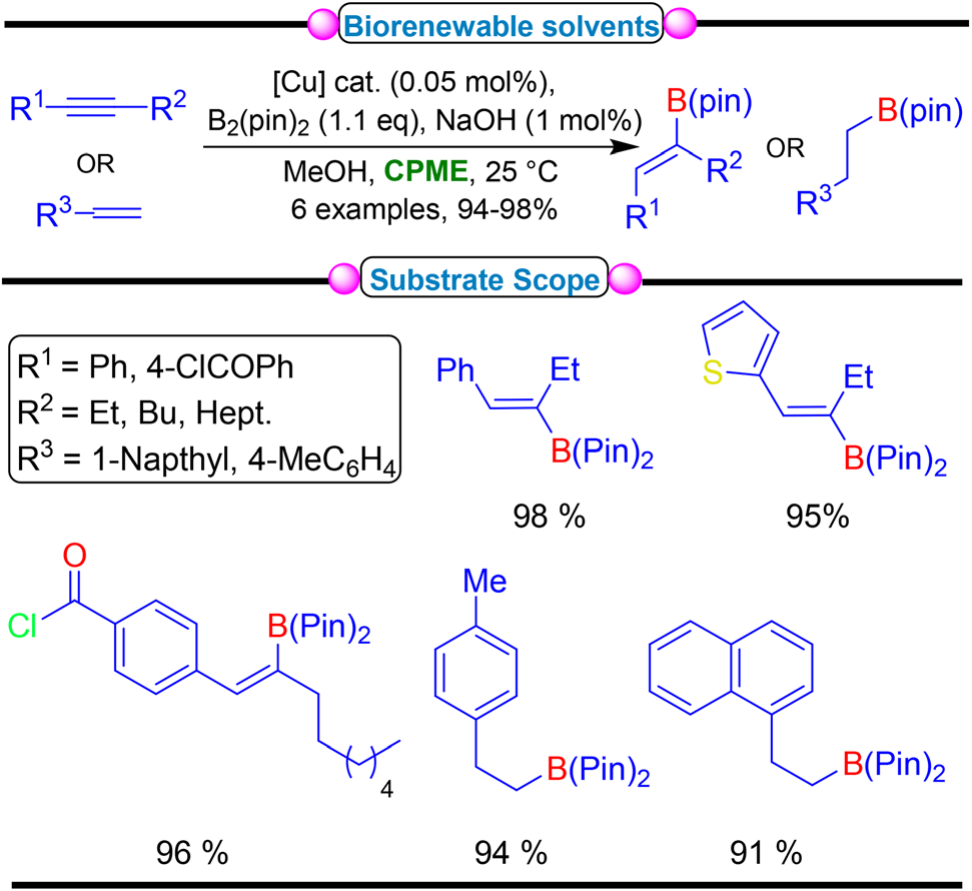
Synthesis of NHC–copper complexes.

N-heterocyclic carbenes have emerged as a robust option for numerous catalytic transformations.^277–279^ These complexes either had a ditopic bisthione ligand or were produced as copper sulphide clusters with a (NHC)Cu_2_(µ-S) unit. Szadkowska *et al.* reported a new series of NHC copper complexes containing sulfoxide and sulfone moieties to perform C–H bond functionalisation (Scheme 63).^280^ The transformation was carried out using terminal and internal alkynes, alkenes, the synthesised catalyst, and a base. The whole process was completed using bispinacolate as the boron source and CPME as the reaction medium. CPME has also been used as a cosolvent (in conjunction with methanol) and employed as a stabiliser for borylation reactions. As illustrated in (Scheme 63), both electron-deficient and electron-rich substrates with six different alkenes and alkynes gave a pinacol boronic esters up to a 98% yield.

**Scheme 64:**
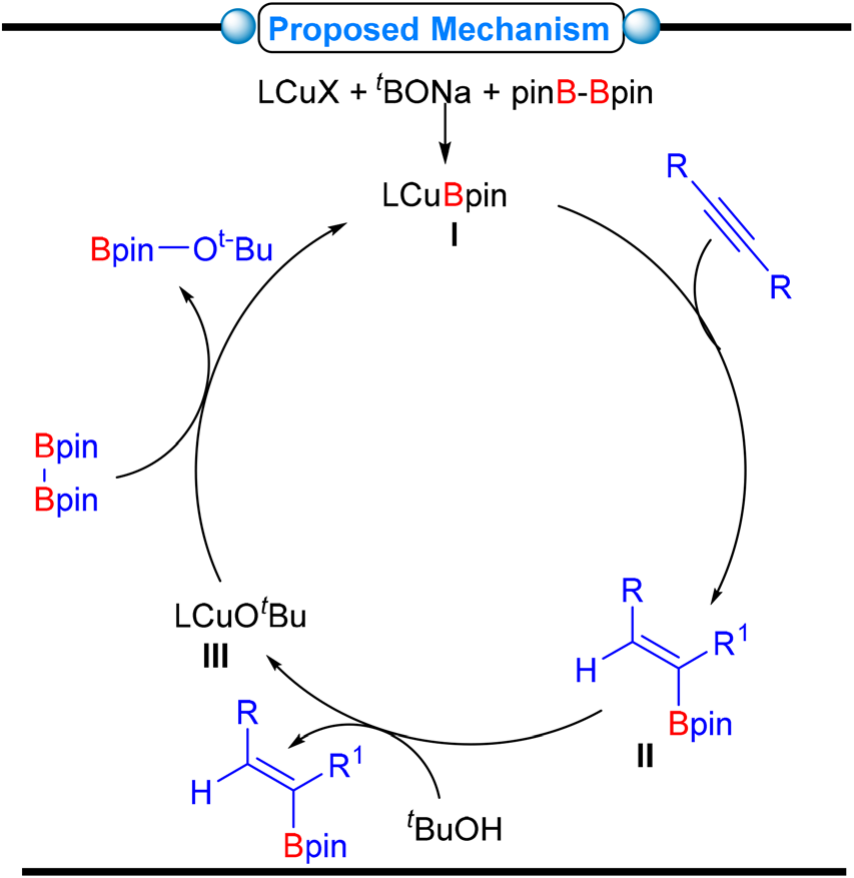
Plausible catalytic cycle for the NHC copper-catalysed reactions in CPME.

A plausible mechanistic pathway (Scheme 64) was drawn for the above-mentioned hydroboration reaction. At first, copper pinacolate I intermediate was generated by the reaction between NHC–Cu complex bispinacolatodiborane. The copper pinacolate intermediate then reacted with alkyne to form the intermediate II, which then formed the intermediate III by the elimination of alkene. In the final step, the intermediate I was regenerated upon reaction with bispinacolatodiborane.

**Scheme 65:**
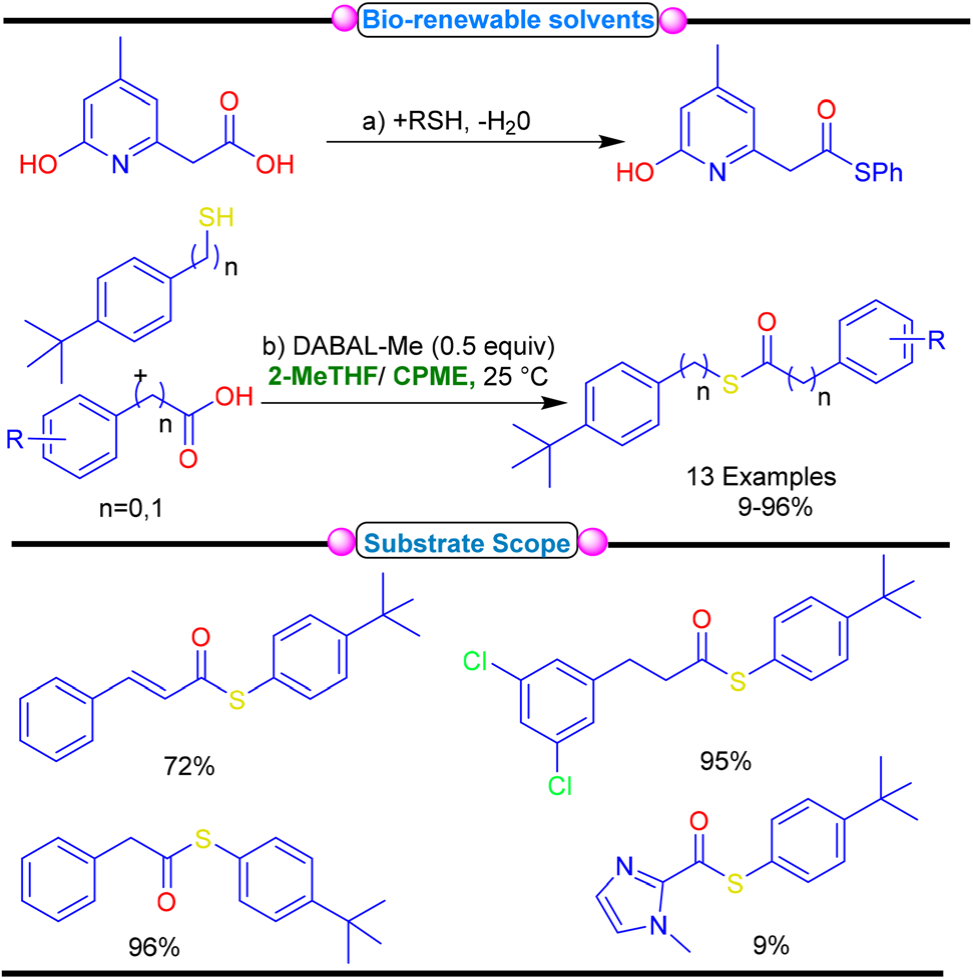
Synthesis of thioesters.

Jordan *et al.* (2019) successfully developed the conditions for a solvent reagent guide for the preparation of thioesters using CPME and 2-MeTHF solvents, which were proven to be more suitable for the preparation of bioactive molecules (Scheme 65b).^281^ When thienyl molecules were utilised directly with carboxylic acids, the alternative reagent DABAL-Me3 proved limited in this work, and none of the heterogeneous reagents evaluated achieved effective conversions to product. Using the solvent-reagent combination, molecules bearing a C–S bond were effectively formed with moderate to excellent yields, demonstrating the system's broad applicability. Prior evaluation of the synthesis of thioesters showed hazardous solvents and unreactive carboxylic acids (Scheme 65a).^282^

### 4.4. Cyrene

**Scheme 66:**
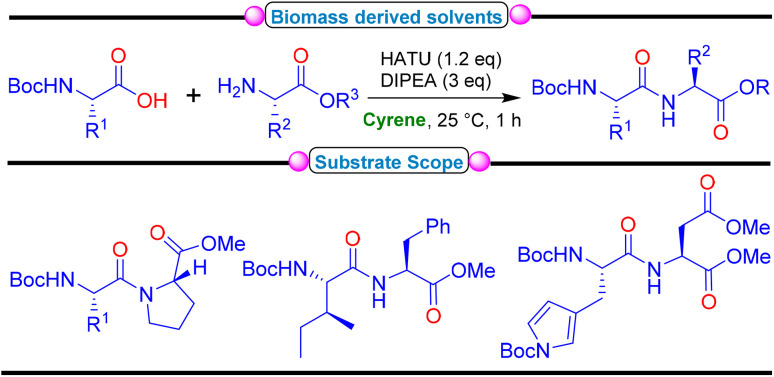
HATU-mediated amide bond formation using Cyrene as a solvent.

The usefulness of Cyrene as a biomass-derived solvent in HATU-mediated amide bond formation has been assessed as a direct substitute for traditional dipolar aprotic solvents (DMF, NMP) by Watson *et al.* in 2018.^283^ HATU was tested as the coupling reagent in the presence of *para*-toluic acid and functionalized anilines as substrates (Scheme 66). Using the developed synthetic approach, versatile peptides and compounds with potential medicinal value (25 examples and yield up to 100%) were produced. The devised technique shows tolerance to various functional groups and great generality, making it suitable for peptide and small molecule production. It is important to note that greater yields with less variation were seen when the stirring rate was increased. It was explained away by stating that because Cyrene is a viscous liquid, slow stirring speeds may result in less-than-ideal mixing. However, a reduced yield dependence on stirring rate was noted upon increasing the stoichiometry of DIPEA. Adjusting the coupling reagent and base did not result in a higher conversion rate.

**Scheme 67:**
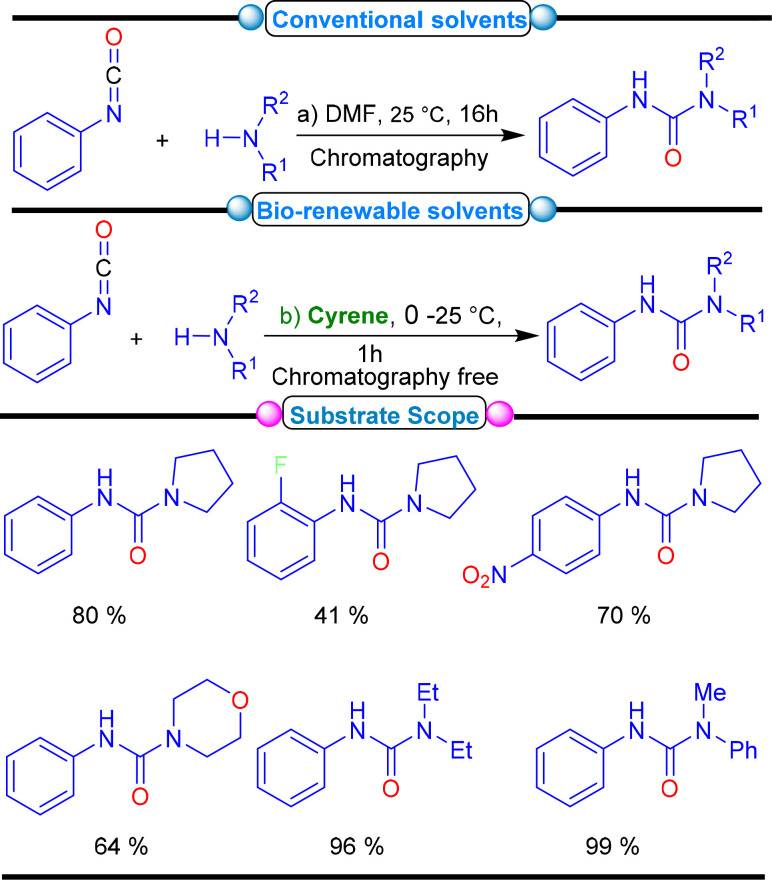
Synthesis of ureas from phenyl isocyanate and amines in Cyrene.

Mistry's group in 2017 reports the first use of Cyrene as the solvent in regioselective catalytic domino-reactions for the synthesis of urea from aryl isocyanates and secondary amines (Scheme 67b).^284^ Investigations were conducted on the amine nucleophiles as well as the impact of substitution on the aryl isocyanate. This technique offers a significant substitute for the industrial usage of halogenated solvents, DMF and non-bioderived organic solvents (Scheme 67a).^285,286^ It was observed that pyrrolidine, cyclic amines piperidine, morpholine, 1,2,3,4-tetrahydroisoquinoline and bis-*N*-allylamine generated the necessary ureas, in high yields. Urea were also produced in good yields by *N*,*N*-dialkyl amines with a chain of 2–8 carbon atoms long. Ultimately, it was discovered that the precipitation of the intended urea occurred when water was added to the Cyrene solution after the completion of the reaction.

**Scheme 68:**
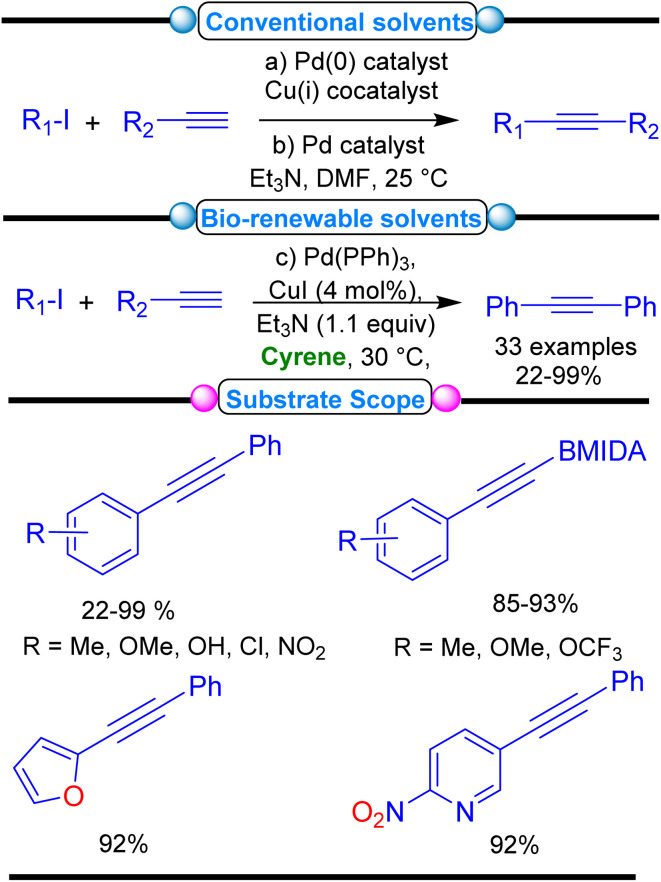
Cyrene-based Sonogashira cross-coupling reaction.

Several researchers have developed the Sonogashira reaction by using a range of dipolar aprotic solvents specifically DMF, which is considered to be harmful to the environment (Scheme 68a and b).^287,288^ Wilson *et al.* created a gentle and reliable technique for the Sonogashira reaction by using Cyrene, a sustainably sourced and bioderived substitute for DMF (Scheme 68c).^289^ A standard catalyst system developed from literature was used (Pd(PPh_3_)_2_Cl_2_ with CuI addition). A broad range of functional groups like methyl (–Me) and methoxy (–OMe) added to aromatic amines at various locations have been shown to be useful in establishing efficient transformation, resulting in the formation of products in excellent yields ranging from 87 to 96%. Conversely, electron-deficient substituents embedded on aromatic rings in mono- or disubstituted patterns, such as bromo (–Br), chloro (–Cl), fluoro (–F), iodo (–I), trifluoromethyl (–CF_3_), and trifluoromethoxy (–OCF_3_) groups, demonstrated relatively low efficiency to yield the corresponding products in high yields.

**Scheme 69:**
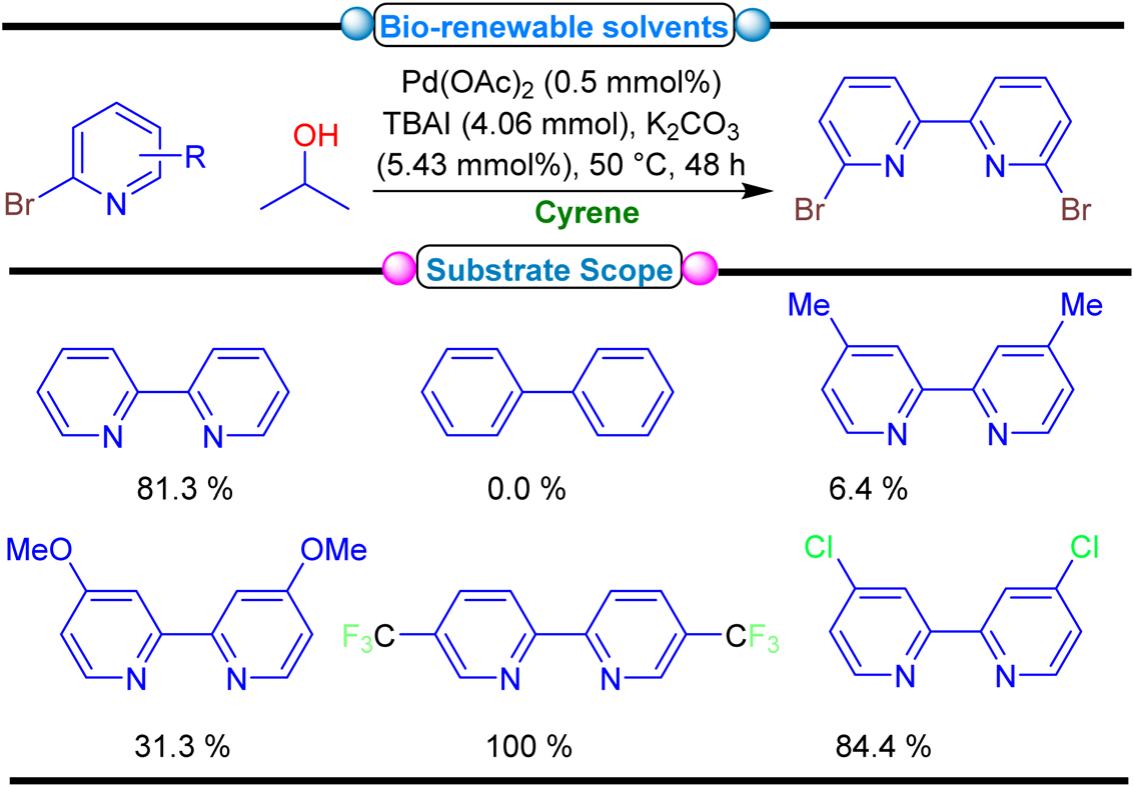
Substrate scope of reductive homocoupling reactions.

In the same year, Webb *et al.* developed a gentle and reliable technique for the C–C bond forming reaction by using Cyrene (dihydrolevoglucosenone), a bio-derived and sustainable substitute for traditional solvents (Scheme 69).^290^ Furthermore, they demonstrated the ability to expand the use of other biomass-derived and traditional solvents to facilitate this reaction (Table 4).

**Table 4:** Reductive homocoupling of 2-bromo-5-(trifluoromethyl)pyridine in different solvent

Solvent	Conversion after 24 h	Solvent	Conversion after 24 h
Cyrene	98	NMP	16
DMF	53	1-Butanal	11
MeCN	59	DME	7
GVL	53	H_2_O	0
2-MeTHF	23		

Possibly of greater significance, D. A. Webb recorded several constraints associated with the application of biomass-derived solvents, including base stabilities of this encouraging environmentally friendly solvent. While the optimization was performed, it was noticed that among the bio-renewable solvents, Cyrene might serve as a direct and functional substitute because it does not currently have any negative side effects and a higher conversion efficiency. When the time rose from 4 h to 24 h, the conversion efficiency was raised from 0.0% to 60.9% in case of 2-bromo-5-(trifluoromethyl)pyridine. The reaction was more sensitive to heteroaromatic substrates, leads to higher conversion whereas conversion rate in the reductive homocoupling of bromobenzene was 0.0% even after 48 h of time.

### 4.5. Polyethylene glycol (PEG)

**Scheme 70:**
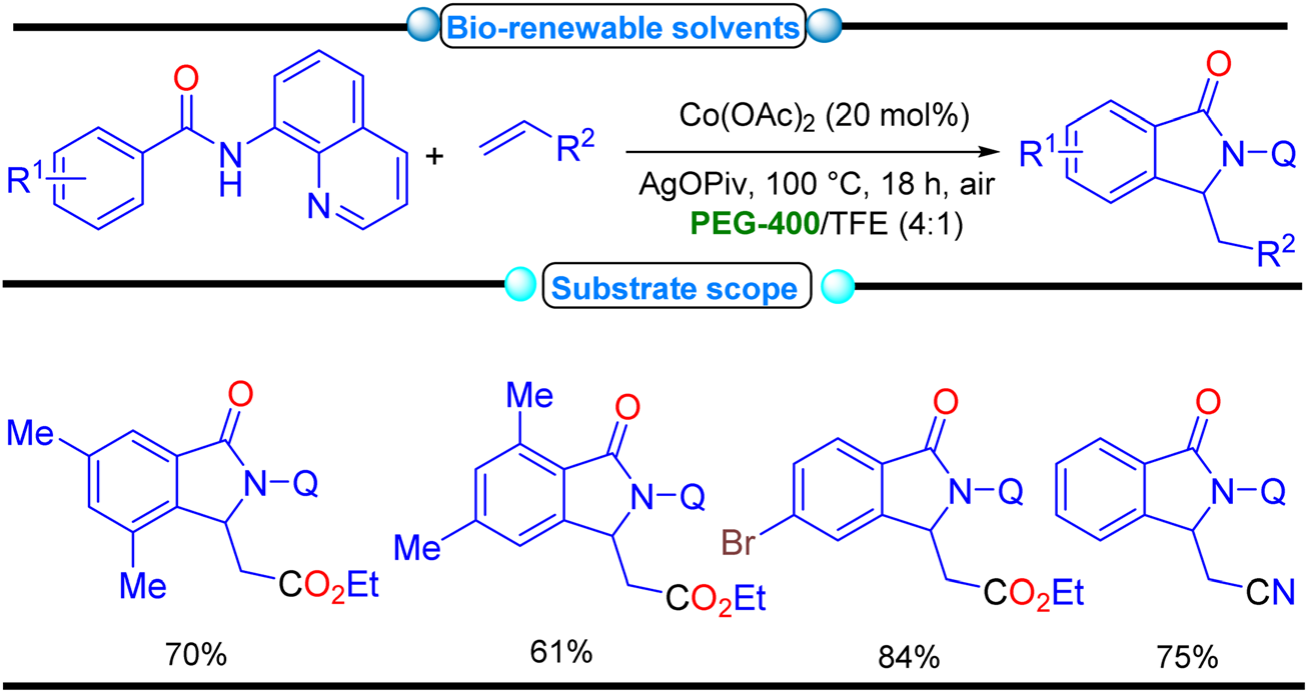
Scope of cobalt-catalysed isoindolinone synthesis in the PEG-400/TFE solvent system.

Ackermann *et al.* in 2015 (ref. 291) discussed C–H/N–H functionalization by cobalt catalysts to provide isoindolinones in an atom- and step-efficient manner (Scheme 70). A carboxylate-assisted C–H cobaltation that was relevant in terms of kinetics was supported by a high-valent cobalt catalyst that was produced in a solvent combination that included PEG. Furthermore, Ag(i) was used as an oxidant in the catalytic system to manufacture isoindolinone derivatives. Effective oxidative annulation of benzamides was observed with various electron-deficient alkenes. The outstanding chemoselectivity of the high-valent cobalt catalyst was demonstrated by effective C–H/N–H functionalisation of substrates bearing a range of reactive electrophilic functional groups, including chloro, bromo, iodo, cyano, and nitro substituents. The substrate scope of the devised protocols was also explored with vinyl esters, ketones, and nitriles that were substituted differently.

**Scheme 71:**
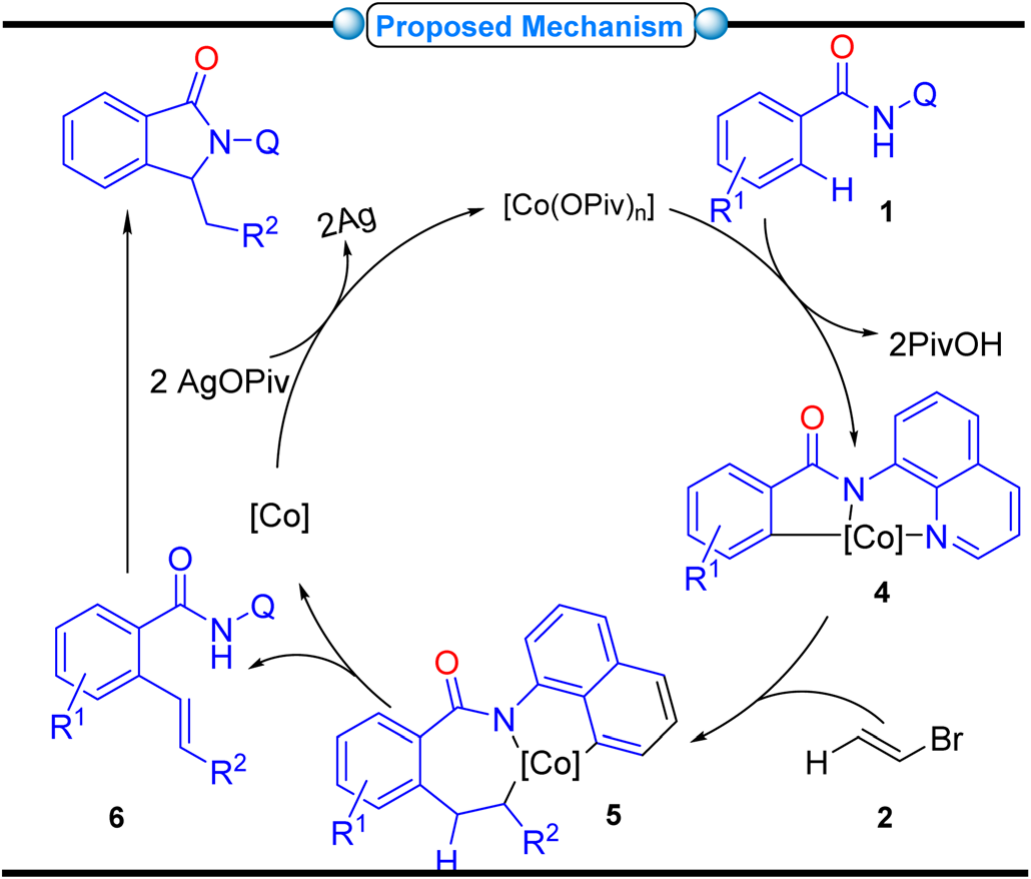
Proposed catalytic cycle of the isoindolinone synthesis.

Based on kinetic isotope study, it was suggested that a kinetically significant carboxylate-assisted C–H cobaltation carried out the oxidative alkene annulation (Scheme 71). This also explained why electron-deficient arenes 1 should be preferentially functionalized in the intermolecular competition tests. The olefin 2 migrated later and was eliminated by β-hydride, resulting in the alkenylated benzamide 6. This alkene hydroamidation occurred intramolecularly to produce the required isoindolinones 3.

**Scheme 72:**
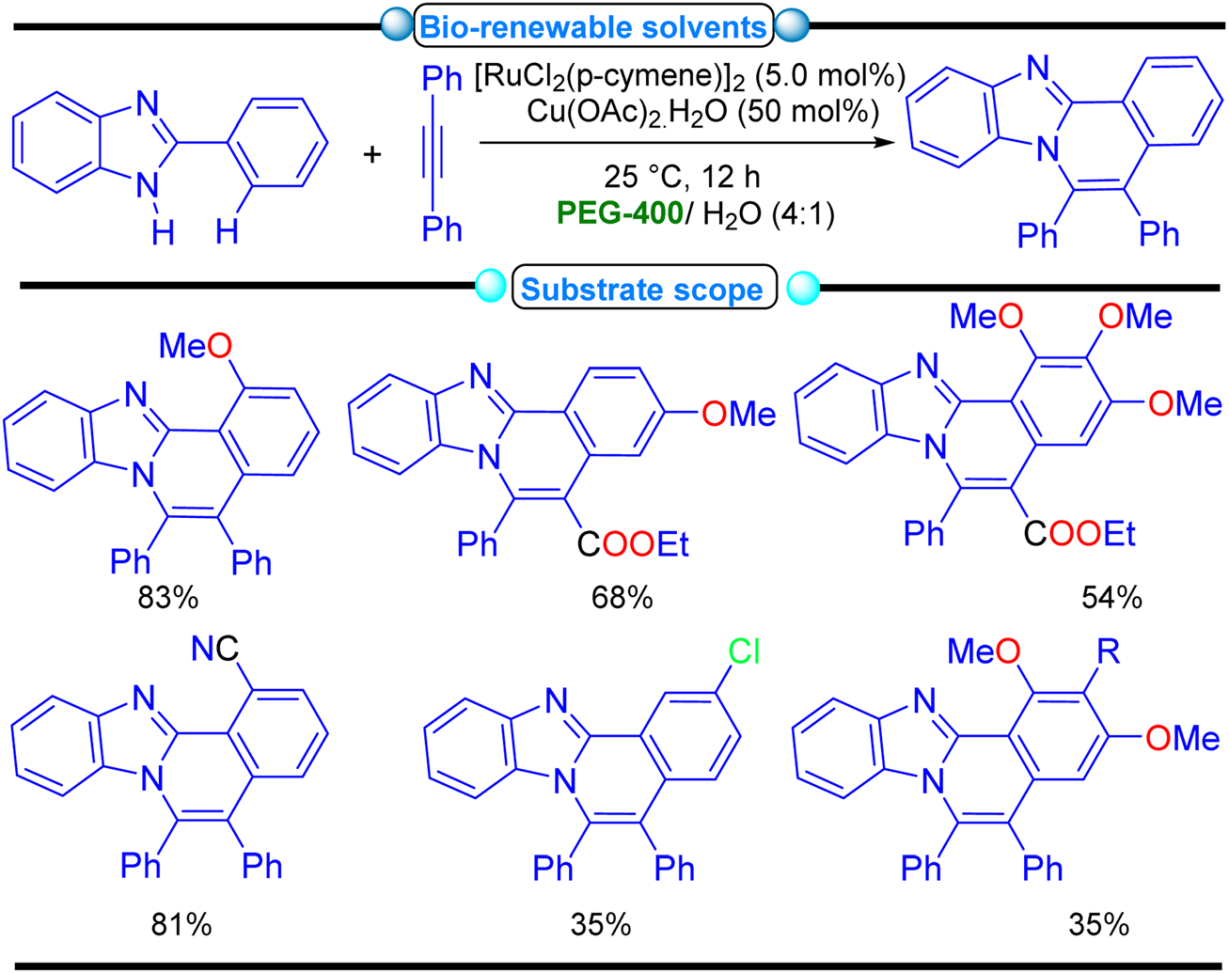
Synthesis of benzimidazoisoquinoline in PEG-400.

The Ru-catalysed reaction of alkynes with 2-aryl benzimidazoles in the presence of Cu salts has led to the synthesis of benzimidazoisoquinoline in an efficient way which is reported in 2013 by Kavitha *et al.* (Scheme 72).^292^ It was also effectively accomplished to react benzimidazoles with unsymmetrical acetylene in a highly regioselective manner. It was suggested that the product outcome was unaffected by the kind of substitution on the 2-aryl group at *para*-position (Br, Cl, F, NEt_2_, Me, OMe, CN, and CF_3_). Moderate yields of the required benzimidazoisoquinolines were obtained using the 2-aryl group on benzimidazole with *ortho*-substitution. Additionally, all reactions were carried out in PEG-400 as a solvent medium resulting in the synthesis of desirable products in similar yields only at ambient temperature. Furthermore, it was also reported to effectively recycle the catalyst and PEG-400 a few times with little to no activity loss.

**Scheme 73:**
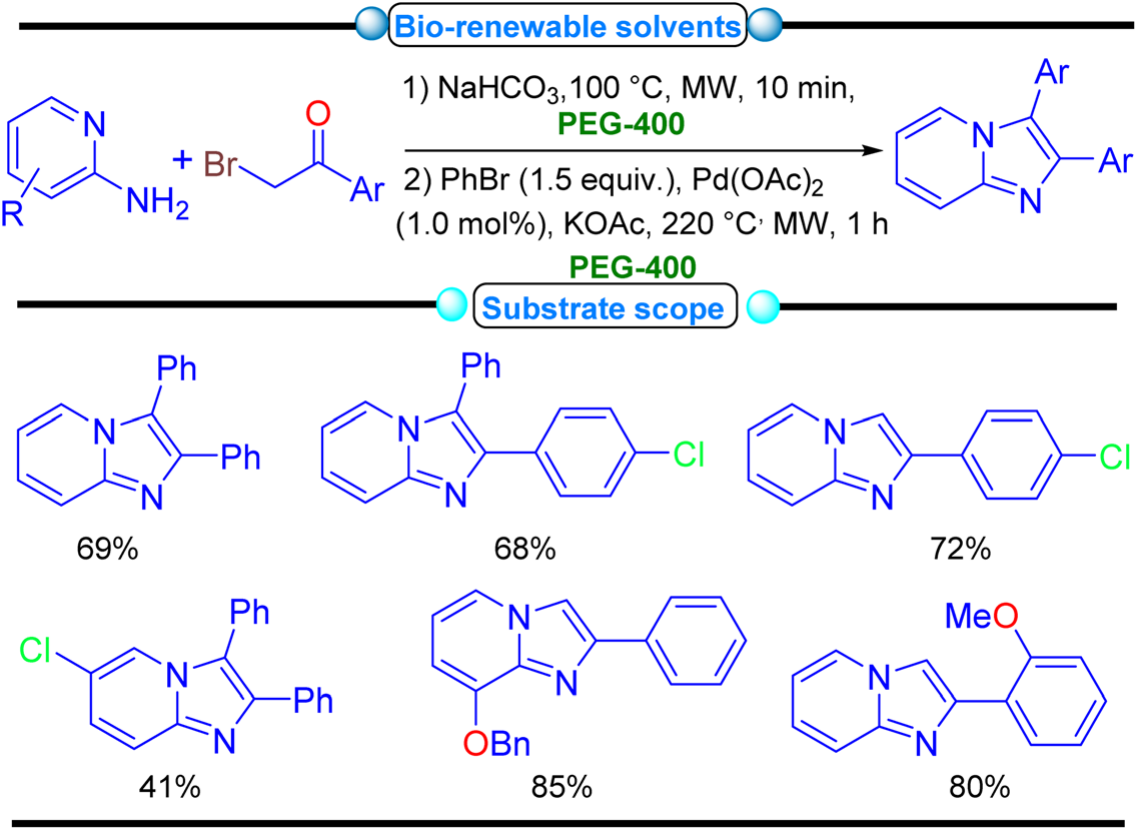
Synthesis of 2,3-diarylimidazo[1,2-*a*]pyridines in PEG-400 medium.

The same was reported by Hiebel *et al.* in 2014 (ref. 293) using PEG-400 as the solvent; studies showed that different 2-aminopyridines could be condensed with α-bromoketones to quickly access 2-arylimidazo[1,2-*a*]pyridines using microwave irradiation in moderate to good yields (Scheme 73). The production of 2,3-diarylimidazo[1,2-*a*]pyridines was enabled by a practical one-pot method that employed a low loading of palladium-catalyst, ligand-free, C–H arylation step in the same environmentally benign reaction medium. The process has been proposed to start with the condensation of various 2-aminopyridines and α-bromoketones to generate the 2-arylimidazo[1,2-*a*]pyridine core. This is then immediately followed by C–H activation at C3.

**Scheme 74:**
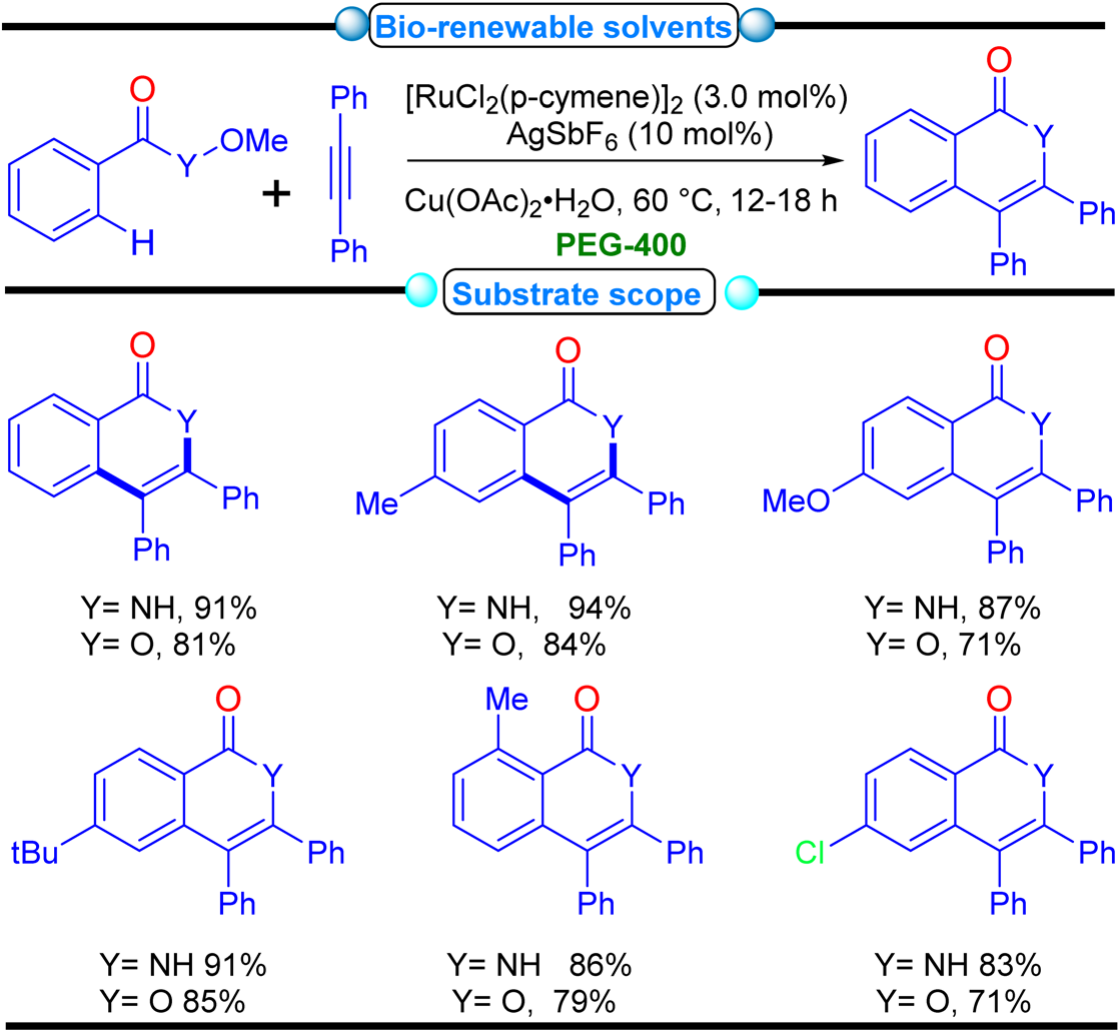
Synthesis of isoquinolinones and isocoumarins using the Ru(ii) catalyst in the PEG-400 medium.

Bhanage *et al.* in 2016 (ref. 294) detailed the utilization of green and recyclable Ru(ii)/PEG-400 medium to activate the C–H bond during the synthesis of isoquinolinones, isocoumarins, *N*-methyl isoquinolinones and olefination of Weinreb amides (W.A). This established approach is capable of the regioselective and stereoselective production of new C–C, C–O, and C–N bonds by the one step cleavage of C–H, N–H, O–H and N–O bonds (Scheme 74). Due to its unique features, this method is environmentally benign. These features include high atom economy, reuse of a costly homogeneous ruthenium-based catalytic system, mild reaction conditions with an easy extraction process, and, notably, all systems derived from this protocol are scalable to the gram level. Additionally, the devised methodology is beneficial for the stereoselective production of *trans-ortho*-olefinated compounds.

**Scheme 75:**
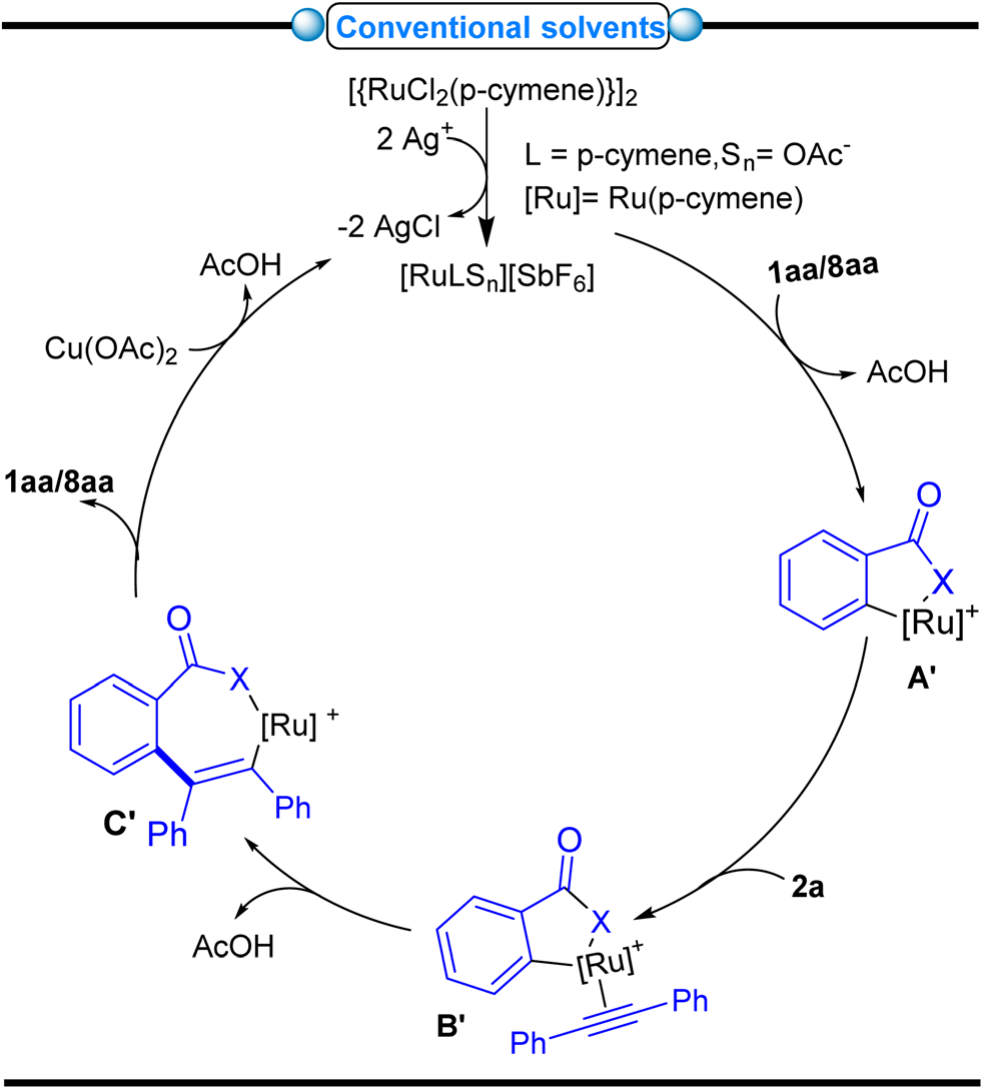
Plausible reaction mechanism of the annulations of *N*-methoxybenzamide and benzoic acid with alkyne.

The proposed mechanism (Scheme 75) involves olefination and annulation of *N*-methoxybenzamide 1a. Ag salt may absorb catalyst's –Cl to create AgCl as the initial stage of the reaction. At first, Ru(ii) combines with hexafluroantimonate to produce a complex, which is then activated by the release of protons from the *ortho*-C–H bond and coordinated with the NH/OH of 1a/8a to form complex A. The coordination of the metal to the alkene/alkyne B′ comes next, and this is followed by carbometallation, which produces intermediates C′. The product 1aa′ is obtained by the reductive elimination of C′ give the annulated product 8aa/1aa′. In the final step of the mechanism Ru^0^ is generated which is oxidised by Cu(ii) to regenerate Ru(ii) for the next catalytic cycle. It is noteworthy to mention that the corresponding gram scale synthesis of the devised protocol has been achieved.

**Scheme 76:**
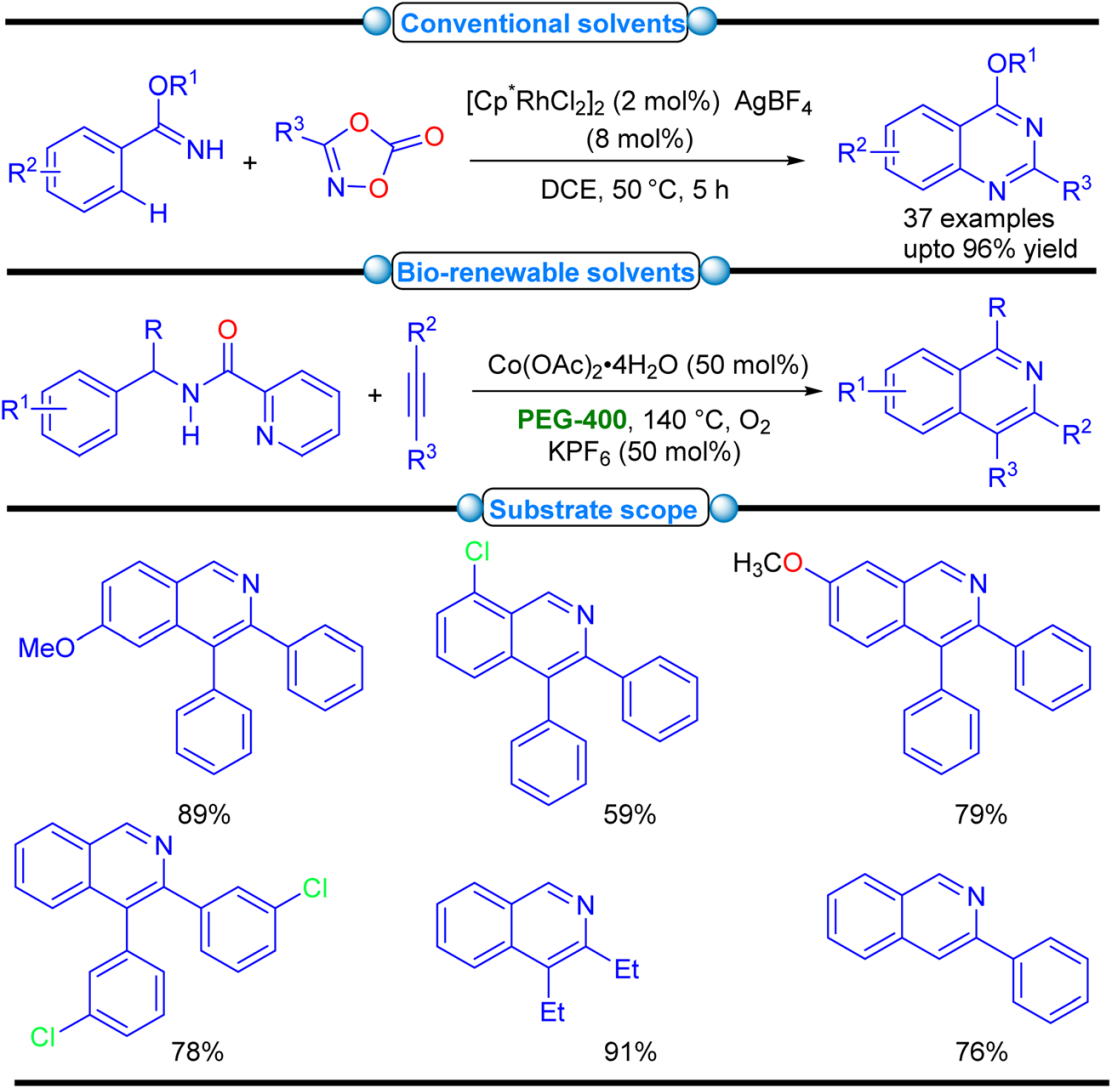
Strategies for the synthesis of isoquinolines.

Cui *et al.* in 2017 first demonstrated a cobalt-catalysed oxidative annulation using picolinamide as a traceless guiding group in the presence of benzylamides with alkynes, which activated C–H/N–H bonds to produce isoquinolines (Scheme 76).^295^ Subsequently, the synthesis of isoquinone was also reported by other authors using conventional solvents.^296,297^ The broad substrate scope for this protocol effectively used with both internal and terminal aryl/aliphatic alkynes, which exhibited strong regioselectivity and good tolerance to functional groups. Moreover, oxygen was employed as the innocuous terminal oxidant rather than a metal-based stoichiometric oxidant.

**Scheme 77:**
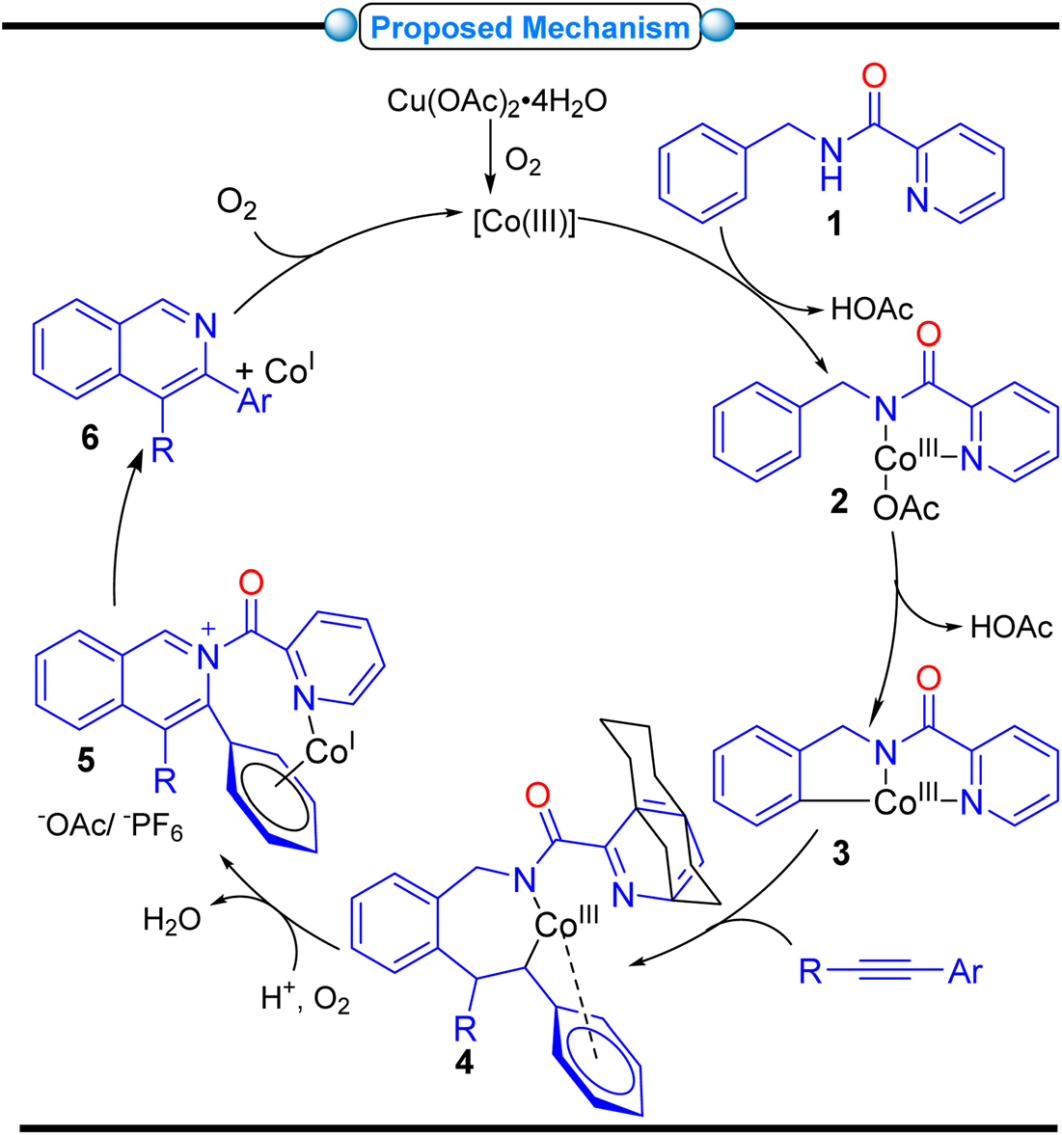
Plausible mechanistic cycle for the synthesis of isoquinolines.

Lower yields were obtained with benzoylamides bearing electron-withdrawing groups than with those bearing electron-donating groups. Alkynes replaced with dialkyl likewise had a smooth reaction, yielding the appropriate product in an 87–91% yield. From deuterium labelling and radical trapping experiments, a plausible mechanistic pathway has been proposed (Scheme 77). The process begins with Co(iii) species generated *in situ* from Co(ii) species by oxygen. Subsequently, intermediate 2 is produced by the coordination of the benzylamide 1 with the cobalt centre, ligand exchange, and simultaneous formation of HOAc. The intermediate 3 is then produced by reversible cyclometallation, most likely by a concerted metalation–deprotonation (CMD) process. After that, intermediate 3 is coordinated and inserted with alkyne to form a seven-membered intermediate 4. This intermediate then undergoes dehydrogenation and reductive elimination with oxygen to produce the isoquinolinium salt 6. Subsequently, the breakage of the C–N bond yields the isoquinoline product and the Co(i) species. This latter was then converted to the active Co(iii) species for the subsequent catalytic cycle by oxygenation.

**Scheme 78:**
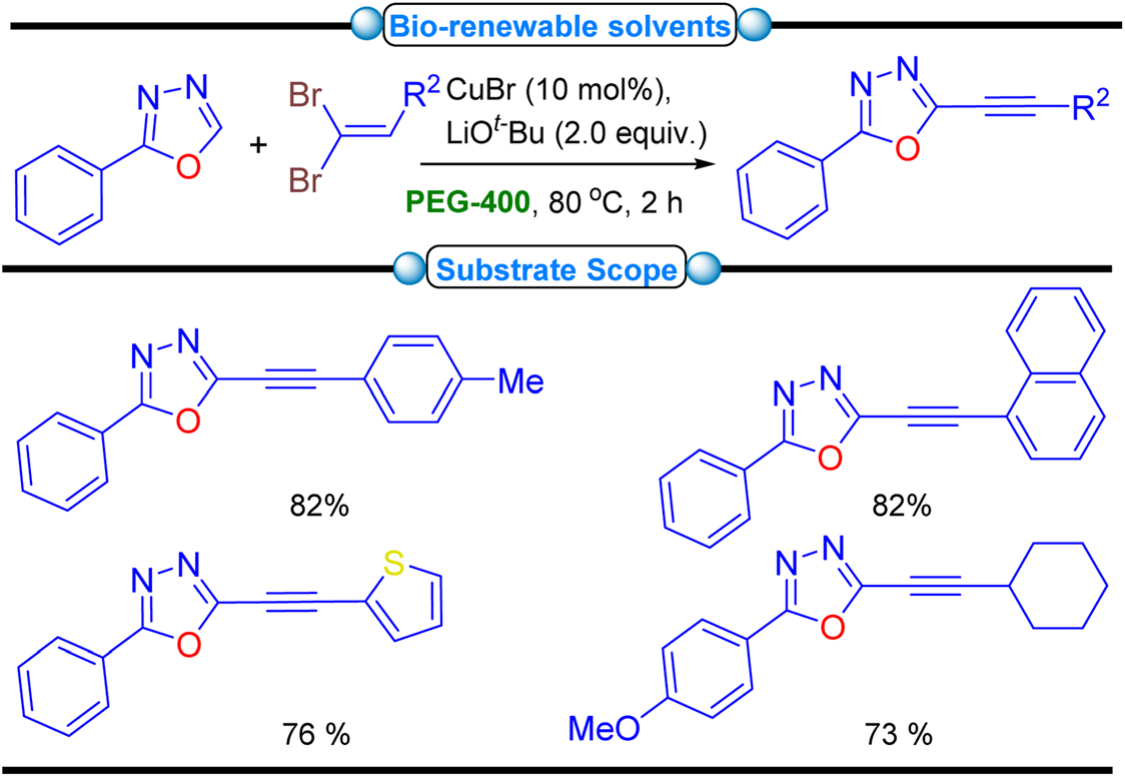
Cu(i)-mediated cross-coupling of 2-phenyl-1,3,4-oxadiazole with 1,1-dibromo-1-alkenes using PEG-400 as a reaction medium.

Das *et al.* in 2012 (ref. 298) have achieved a direct C–H alkynylation of 1,3,4-oxadiazoles with 1,1-dibromo-1-alkenes was achieved at 80 °C by utilizing a mixture of CuBr/LiO^*t*^Bu in green renewable PEG-400 as a solvent in about two hours, the products were generated in high yields (73–86%) (Scheme 78). This method is fascinated with some noteworthy features such as moderate reaction conditions, ease of operation, inhibits the use of ligands and volatile solvents. The conversion was environmentally benign and didn't require the use of ligand or hazardous solvent, working under moderate conditions and affording high yield. Aliphatic, heteroaromatic, and aromatic alkenes converted smoothly into the products with a yield of 73–86%.

**Scheme 79:**
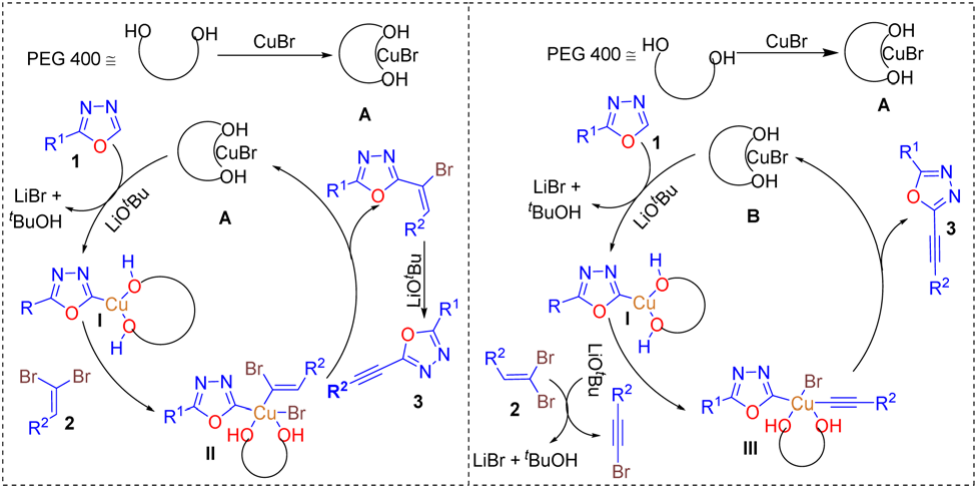
Proposed mechanistic pathway for direct C–H alkynylation.

A plausible mechanistic pathway was put forward by employing polyethylene glycol as the reaction medium and is also presented to function as a ligand to create species A. Next, Cu(iii) complex II and (heteroaryl) CuI intermediate I are involved in the process to create alkynylated product 3 (Scheme 79, A). In a different method involves complex III being formed by the interaction of the alkynyl bromides obtained from the 1,1-dibromo-1-alkenes with I, leading to the production of alkynylated derivative 3 (Scheme 79). On the other hand, a high yield of the alkynylated product was obtained when a 1,3,4-oxadiazole was reacted with a bromoacetylene under comparable reaction conditions. Therefore, it was suggested that the second method (Scheme 79, B) is more suited for the reported conversion.

**Scheme 80:**
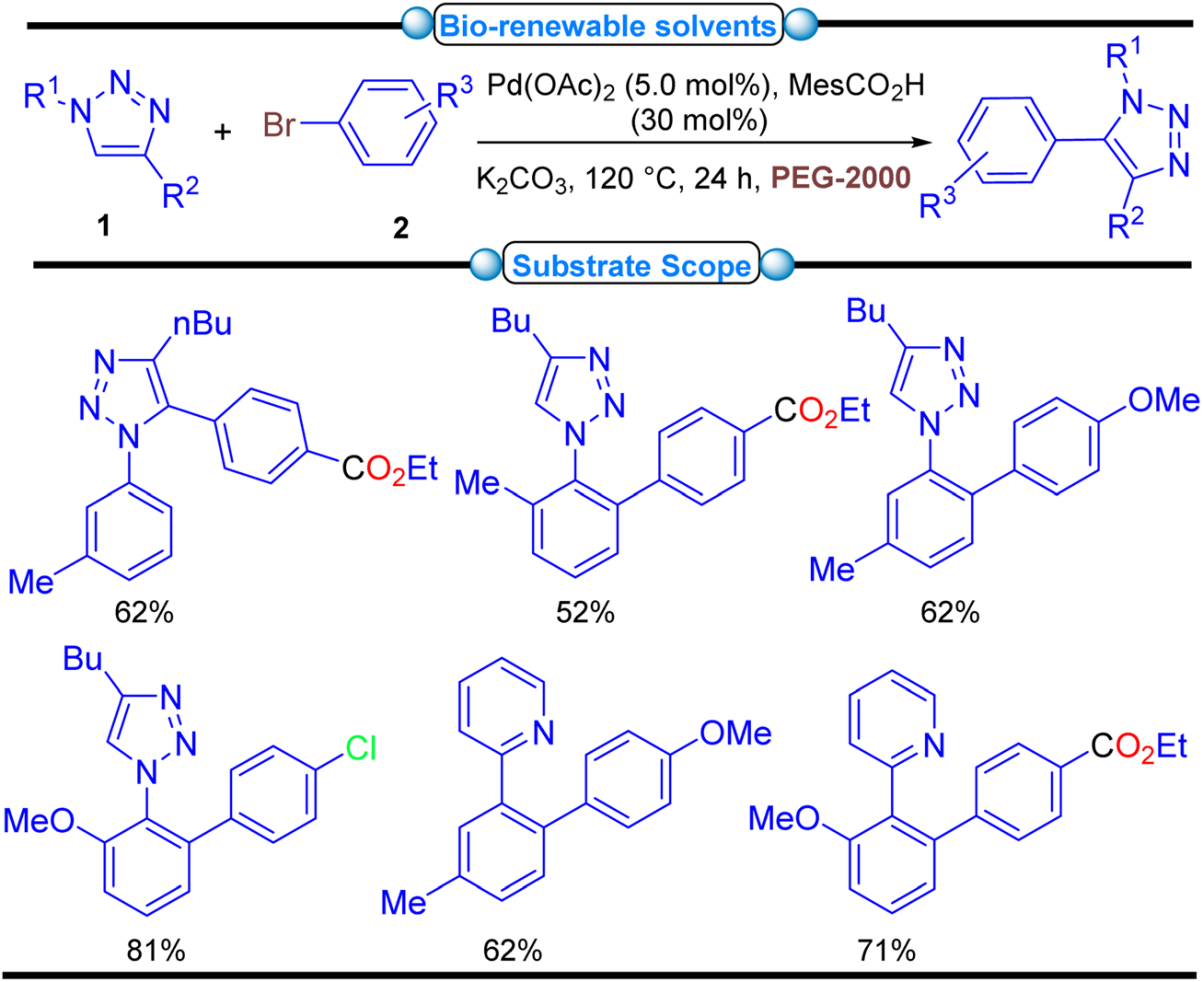
Palladium-catalysed direct C–C bind formation in PEG-2000.

Ackermann *et al.* in 2009 reported the first transition metal catalysed direct arylations in nontoxic PEGs as benign reaction media (Scheme 80).^299^ Significantly, this made it possible for hitherto unheard-of C–H bond arylations to be catalysed by Pd(OAc)_2_ in an airy environment using a convenient, recyclable catalytic system that lacked a phosphine ligand. It is noteworthy to mention that using a palladium catalyst modified with carboxylic acid MesCO_2_H in PEG-20000, which is phosphine ligand-free, resulted in the best reaction conditions. The catalytic system was able to tolerate valuable functional groups, which allowed to produce *N*-aryl triazoles with various substitutions. It was also possible to generate *N*-benzylated triazoles when air was present. Furthermore, the required compounds with good C-5 regioselectivities were obtained by mono-*N*-substituted triazoles. Interestingly, using PEG-20000 as the reaction medium made the Pd(OAc)_2_ catalyst from carboxylic acid easily recyclable.

**Scheme 81:**
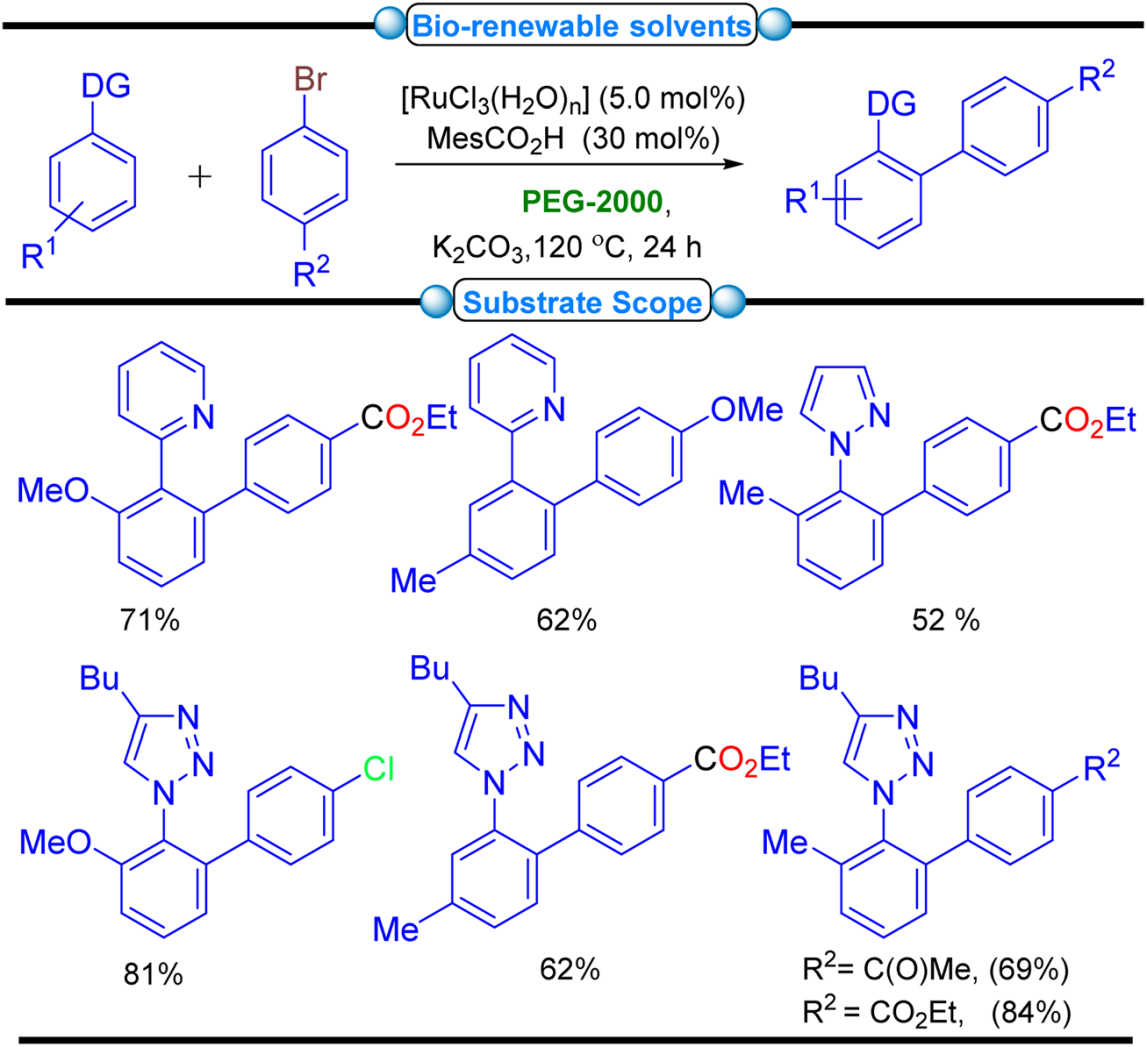
Ruthenium-catalysed direct arylations in PEG-2000.

In the same article Ackermann also reported the synthesis of biphenyl by the activation of C–H bond of aryl bearing electron donating group and aryl halide by using [RuCl_3_(H_2_O)_*n*_] as a catalyst with a co-catalytic amount of MesCO_2_H (2,4,6-trimethylbenzoic acid) to furnish biaryls in desirable yields. The said reaction was carried out by using a renewable solvent as a reaction medium at a temperature of 120 °C (Scheme 81).

**Scheme 82:**
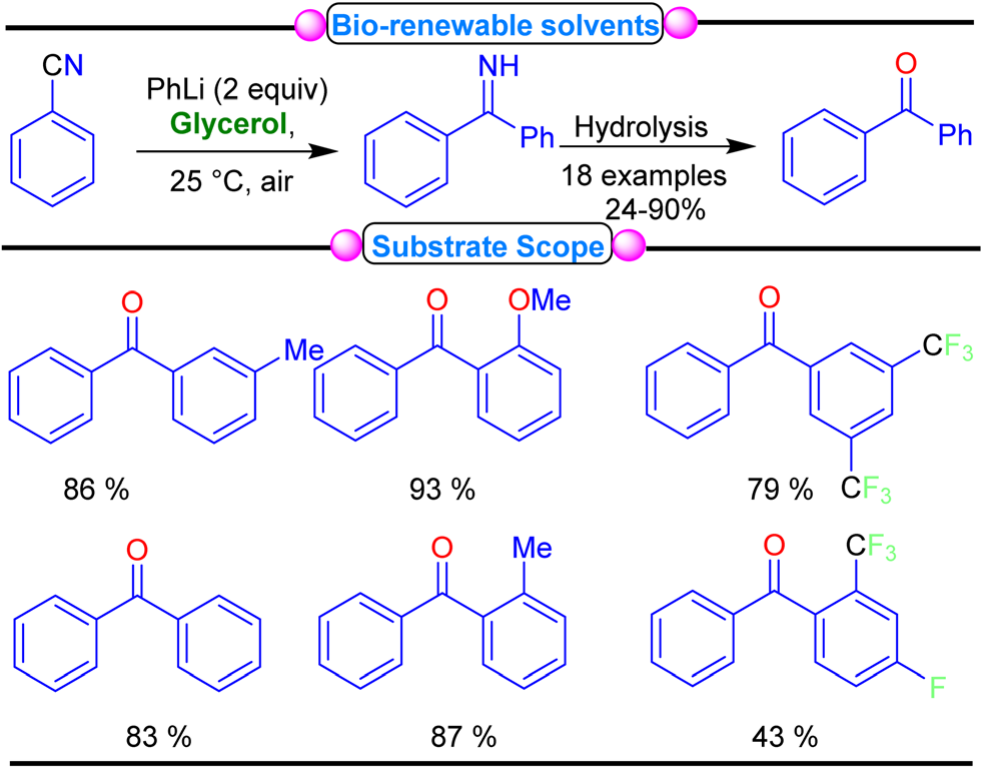
Addition of RMgX to various nitrile in glycerol solvent.

One of the greatest hurdles for synthetic chemists is working in organolithium chemistry under aerobic and/or hydrous conditions without the need for rigorously dry, hazardous organic solvents. The chemo-selective and ultrafast addition of a variety of aryl lithium reagents to nitriles was reported by Alvarez *et al.* in 2018 using glycerol as a solvent. The reaction was performed at ambient temperature in air, thereby creating a novel sustainable pathway to aromatic ketones by following air- and moisture-resistant polar organometallic chemistry (Scheme 82).^300^ In the case of higher ketones with solid nitriles, water afforded higher conversion of ketone compared to glycerol. Excellent yields (76–86%) were reported for both reaction media with liquid aromatic nitriles. The procedure was expanded to include other aryl lithium reagents bearing either electron-donating or electron-withdrawing substituents. Nucleophilic addition of the organolithium reagent to benzonitrile occurred immediately, yielding the non-symmetric diaryl ketones in excellent yields (43–95%) and notable chemoselectivity.

**Scheme 83:**
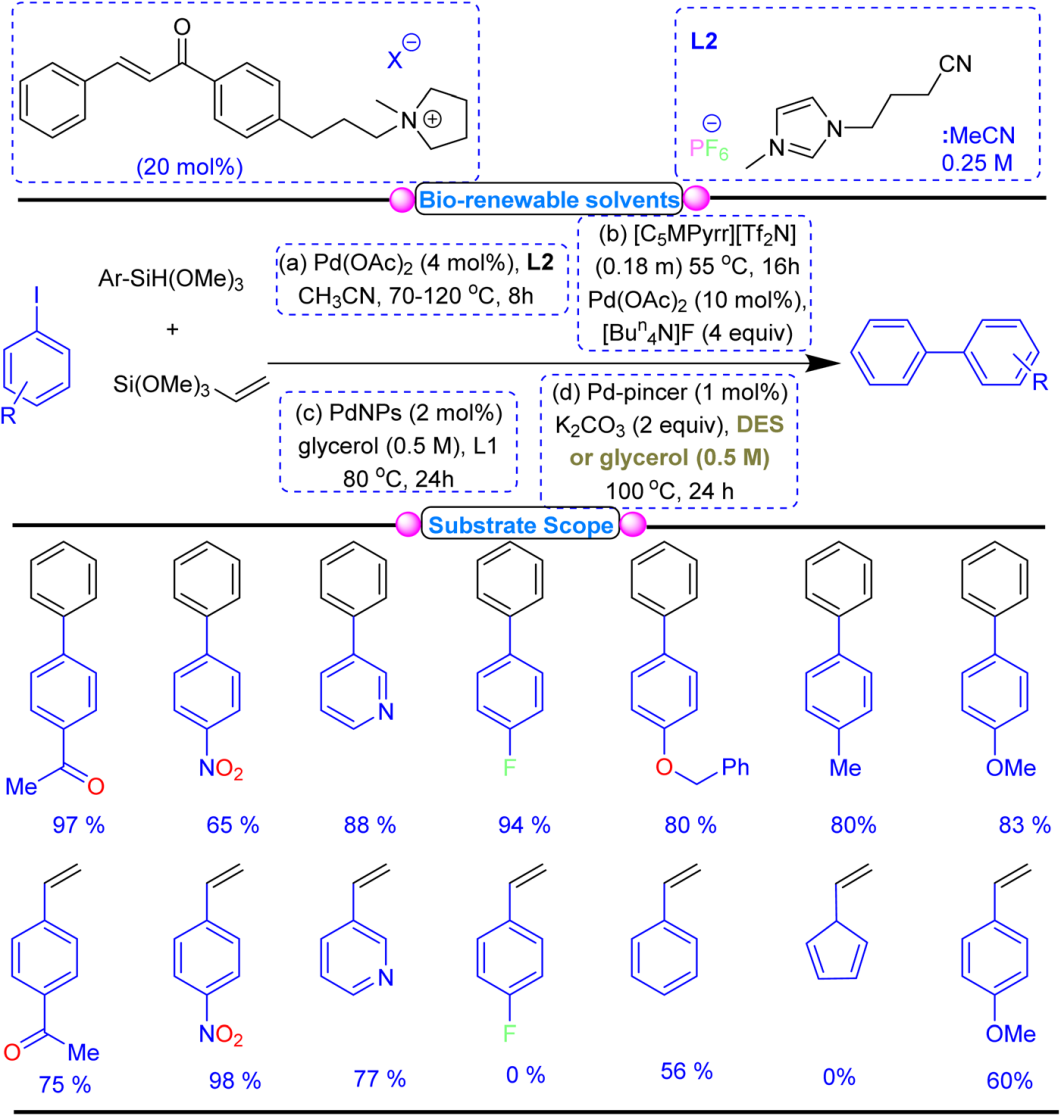
Hiyama-type reaction using neoteric solvents.

Cross-coupling reactions have become tools for C–C bond formation in both minimally functionalized and functionally dense starting materials, which led to the award of Nobel Prizes.^301–303^ In contrast to using an expensive ionic liquid containing fluoride anions, a Hiyama-type process in ionic liquids used 4 mol% Pd without any additions (Scheme 83a).^304^ Bauerlein *et al.* presented another study in this type of solvent using 10 mol% of a Pd precursor, 20 mol% of a complex ligand, equivalents of a fluoride salt (Scheme 83b).^305^ In a different instance, the catalyst for the reaction was 2 mol% of Pd(0) nanoparticles made from Pd(OAc)_2_ and a phosphine ligand (Scheme 83c).^306^ Marset *et al.* (2018) reported a NCN-pincer-Pd complex and used in Hiyama-type cross-coupling reactions between aryl halides and various organosilanes in neoteric solvents.^307^ With respect to cross-coupling reactions, deep eutectic solvents (DES) made of glycerol and choline chloride in a molar ratio of 1 : 2 was used as a biodegradable, non-toxic, non-flammable, and bio-renewable solvent system (Scheme 83d).

### 4.7. Resin and micelle-based reactions

**Scheme 84:**
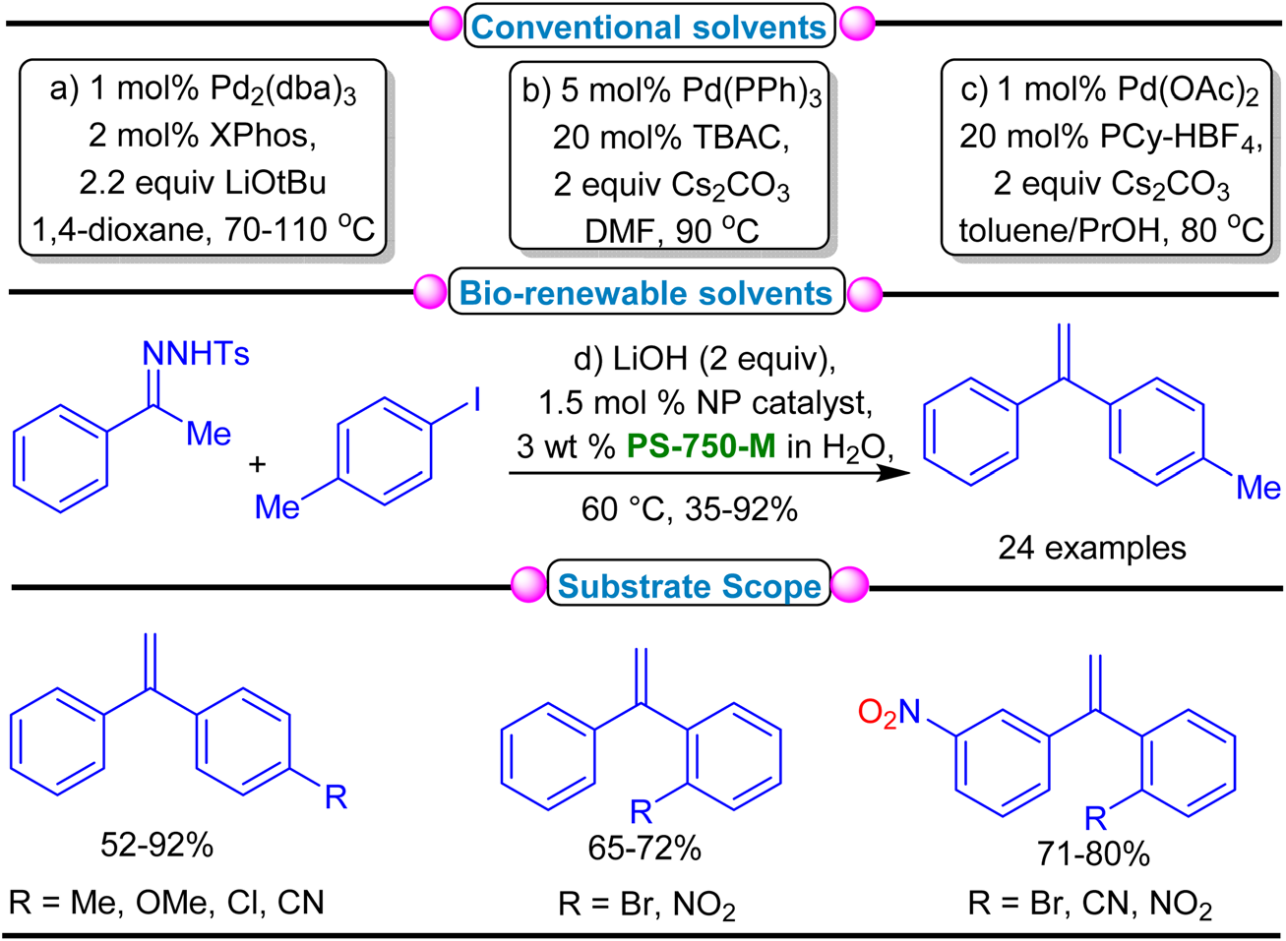
Cross-couplings involving palladium–carbene involving PS-750-M in water.

To investigate the reactivity of carbenes in aqueous nano-micelles, the use palladium nanoparticles ligated with affordable triphenylphosphine and amphiphile PS-750-M were explored by Duong *et al.* The ecstatic selectivity of this synthesised nano-catalyst for metal–carbene migratory insertion, and the micelle of PS-750-M shields the *in situ*-generated carbene to preclude dimerization (Scheme 84d).^308^ The synthesised nano-catalyst were employed to design terminal olefins from *N*-tosylhydrazone and aryl halide in aqueous micelles of PS-750-M. However, this method favours more sustainability and environmental benign to the previous methods by employing recyclable catalyst and a renewable solvent. However, in previous methods, expensive catalysts, and hazardous solvents like 1,4-dioxane (Scheme 84a), toluene (Scheme 84c), and DMF (Scheme 84b) were used under extreme circumstances.^309–311^ Regardless of the steric bulk and electrical properties, the reaction is relatively generic with different combinations of *N*-tosylhydrazone and aryl halides (bromide and iodide).

**Scheme 85:**
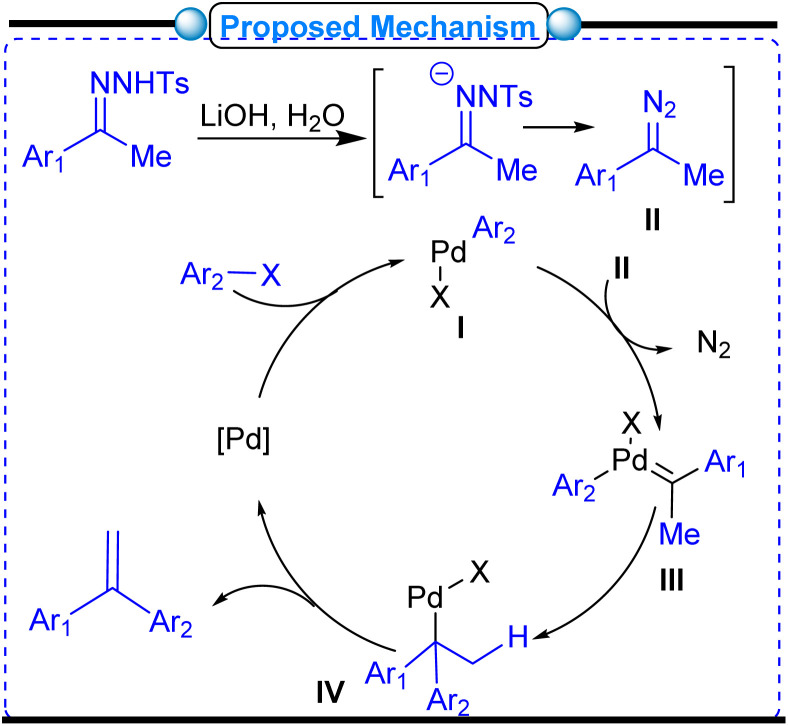
Plausible catalytic pathway.

The mechanism of this reaction has been proposed based on experimental evidence (Scheme 85), the active catalytic centre *i.e.*, Pd lies inside the micelle, so oxidative addition of aryl halide to the active metal to generate intermediate I. Next, the diazo species II are produced inside the micelle by the deprotonation of *N*-tosylhydrazide using OH^−^ at the micellar interface forming *N*-tosylhydrazinide. The inner core of micelle contains PS-750-M mimics polar-aprotic solvent which increase the solubility of intermediate II. Carbene is transferred to intermediate I to form the Pd–carbene intermediate III with the evolution of N_2_ gas, followed by insertion to form an intermediate IV. The end product is released and the active catalyst is regenerated back in the subsequent catalytic cycle through β-hydrogen elimination in IV.

**Scheme 86:**
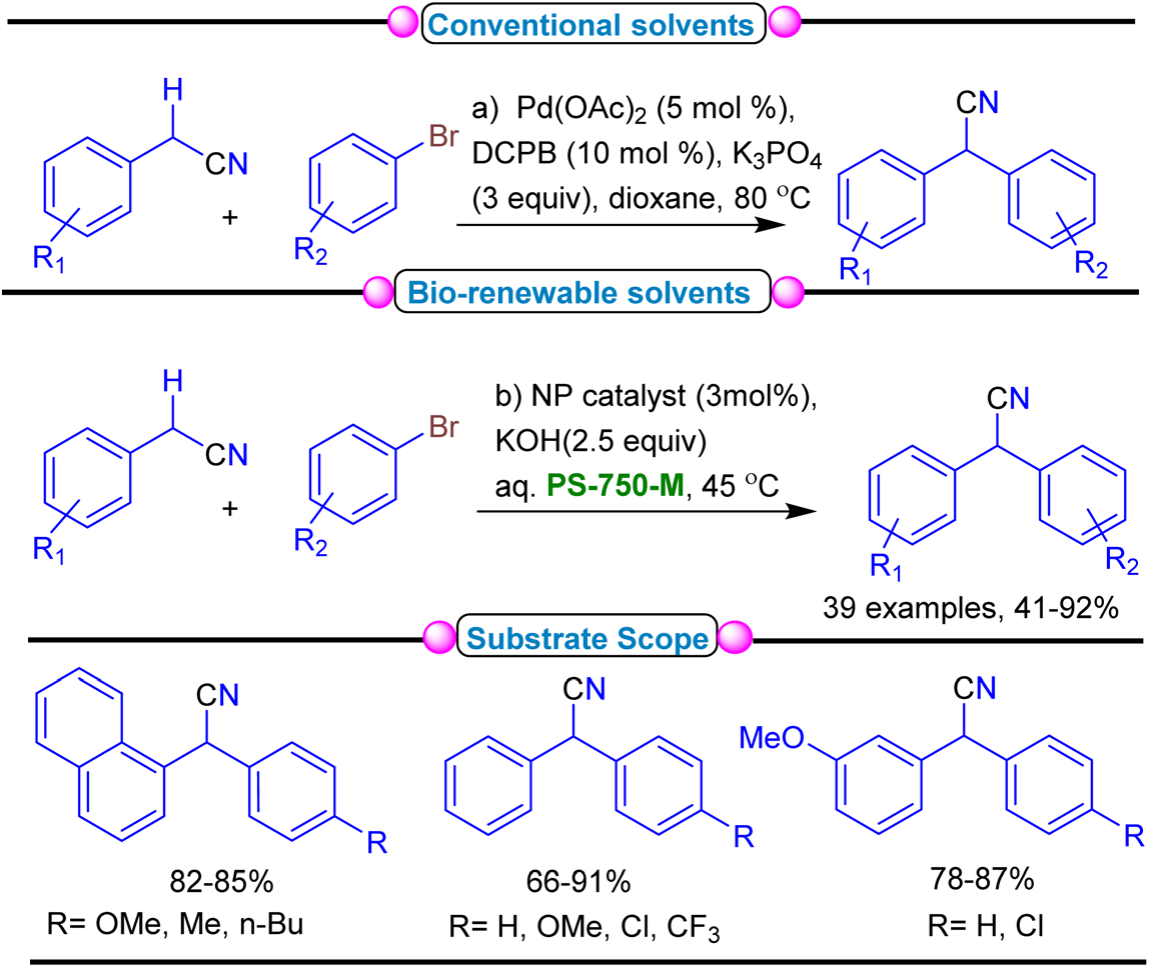
Arylation of nitriles in aqueous micelles using ultra small Pd nanoparticles.

Bihani *et al.*^312^ devised an ecologically acceptable α-arylation process of nitriles in aqueous medium, in which the surfactant PS-750-M plays three crucial functions (Scheme 86b). It enables the quick reductive elimination process that produces and stabilizes ultrasmall Pd NPs. It employs water for larger-scale reactions, eliminating the need for problematic chemical solvents like 1,4-dioxane (Scheme 86a), and stabilizes the carbanion/ketenimine intermediates inside its hydrophobic core, preventing protonation by water.^313^ This reaction exhibits a wide spectrum of substrate tolerance, including steric, electronic, and functional groups. Heterocyclic coupling partners and heterocyclic moieties, such as thiazole, triazole, indole, and pyridyl, were well tolerated and produced α-arylated products.

**Scheme 87:**
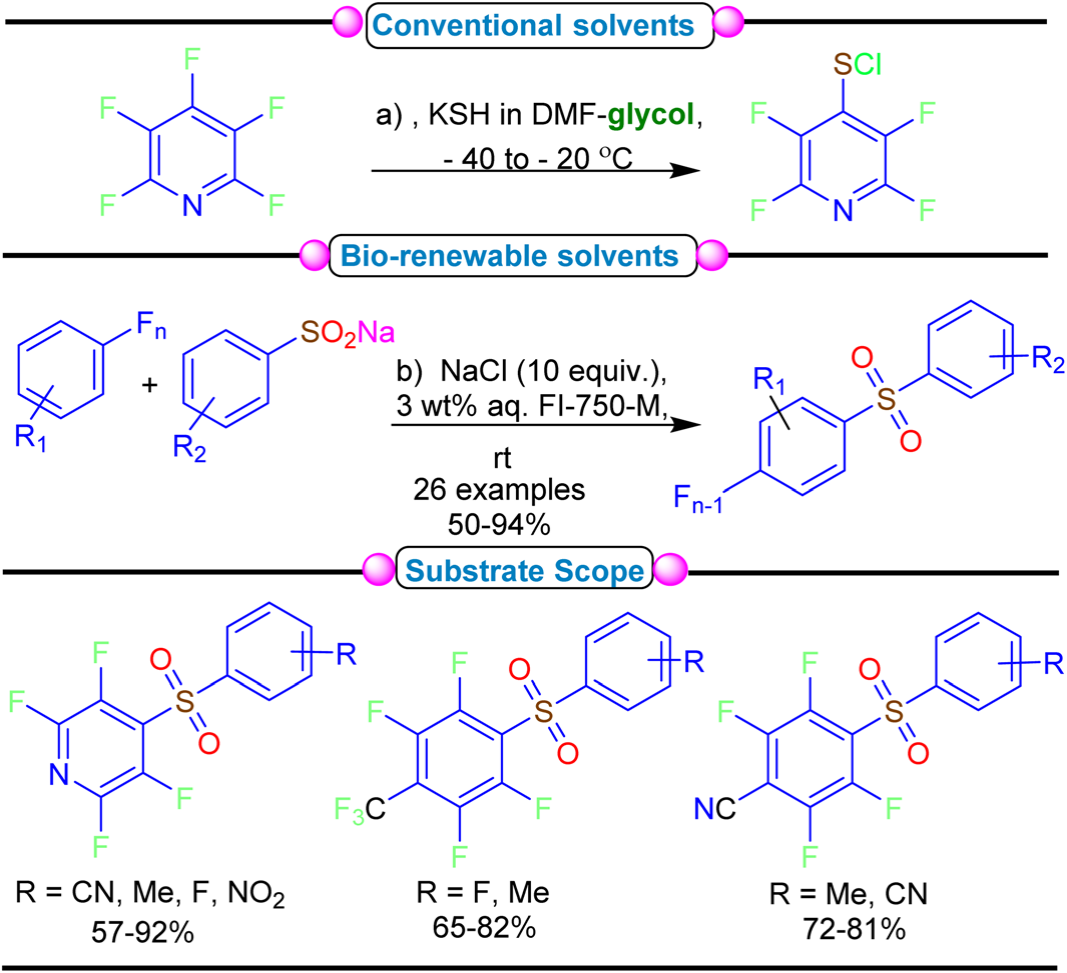
Sulfonylation of polyfluoraromatics using proline-based surfactant FI-750-M.

Smith *et al.* demonstrated that the proline-based surfactant FI-750-M can facilitate the clean, selective sulfonylation of a polyfluoroarene in water under moderate conditions (Scheme 87b).^314^ By adding more polarity to the micellar core, FI-750-M's design aimed to simulate polar-aprotic solvents, which can replace hazardous solvents such as DMF (Scheme 87a).^315^ The effectiveness of this surfactant was compared with that of other surfactants *via* theoretical and experimental studies. Specifically, the FI-750-M linker area was shown to be optimal for the mutual solubility of the polyfluoroarene and the sulfinate anionic nucleophile, according to COSMO-RS simulations. Regarding the sulfinate salt's nature, remarkable universality was observed accommodating systems with significant steric bulk, aryl and heteroaryl combinations, and both electrically rich and deficient systems. Notable steric congestion was seen on substrates with good reactivity, and no polymerised side products were formed.

### 4.8. PS-750-M

**Scheme 88:**
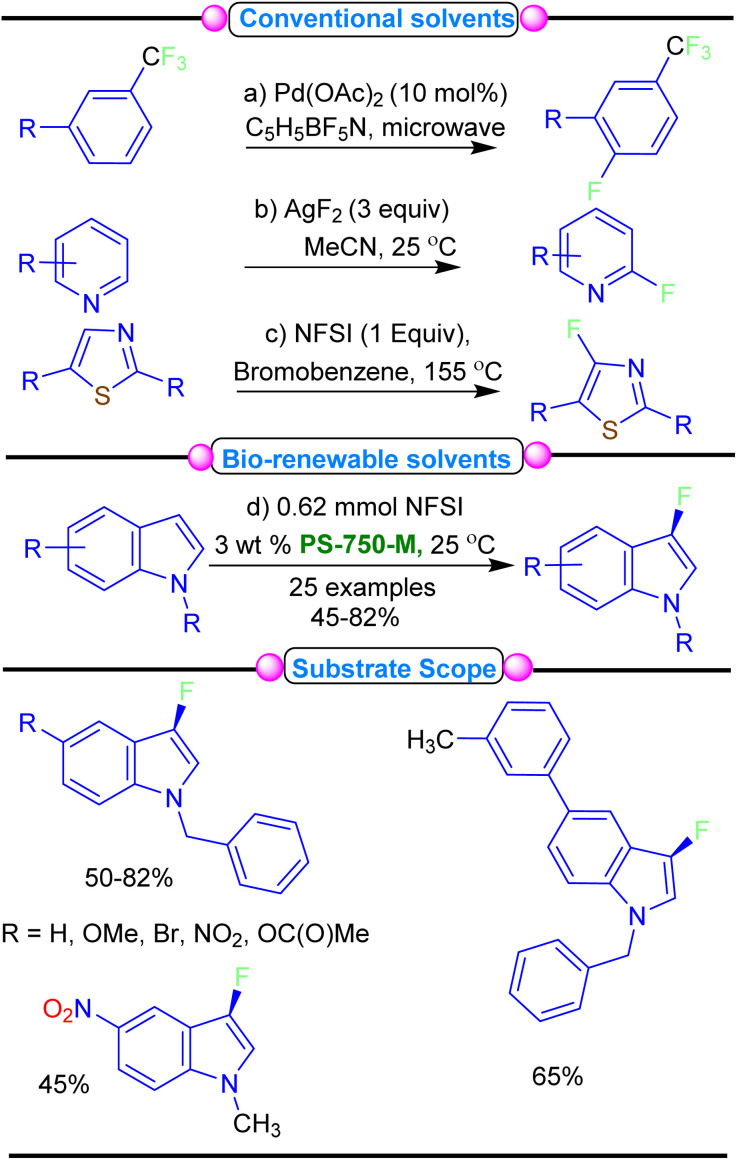
Monofluorination of indoles and arenes *via* C–H activation.

Bora *et al.* (2019) devised a technique for the direct monofluorination of arenes and indoles (Scheme 88d).^316^ The suggested approach was accomplished by employing an amphiphile that produces micelles with somewhat non-polar inner cores, dissolves substrate in the inner core, does not react with any reaction component on its own, and offers very little oxygen solubility inside the core. Remarkably, the reaction was tolerant of numerous functional groups that are susceptible to either radical or electrophilic mechanisms, and it was repeatable at different scales. Interestingly, only the heteroaryl ring of indole underwent fluorination under ideal circumstances, leaving the other aromatic residues untouched. The other aromatic and hetero-reactive moieties were both well tolerated. This method was highly favourable in comparison to microwave (Scheme 88a), acetonitrile (Scheme 87b) and bromobenzene (Scheme 87c), as seen in prior instances.^317–319^

**Scheme 89:**
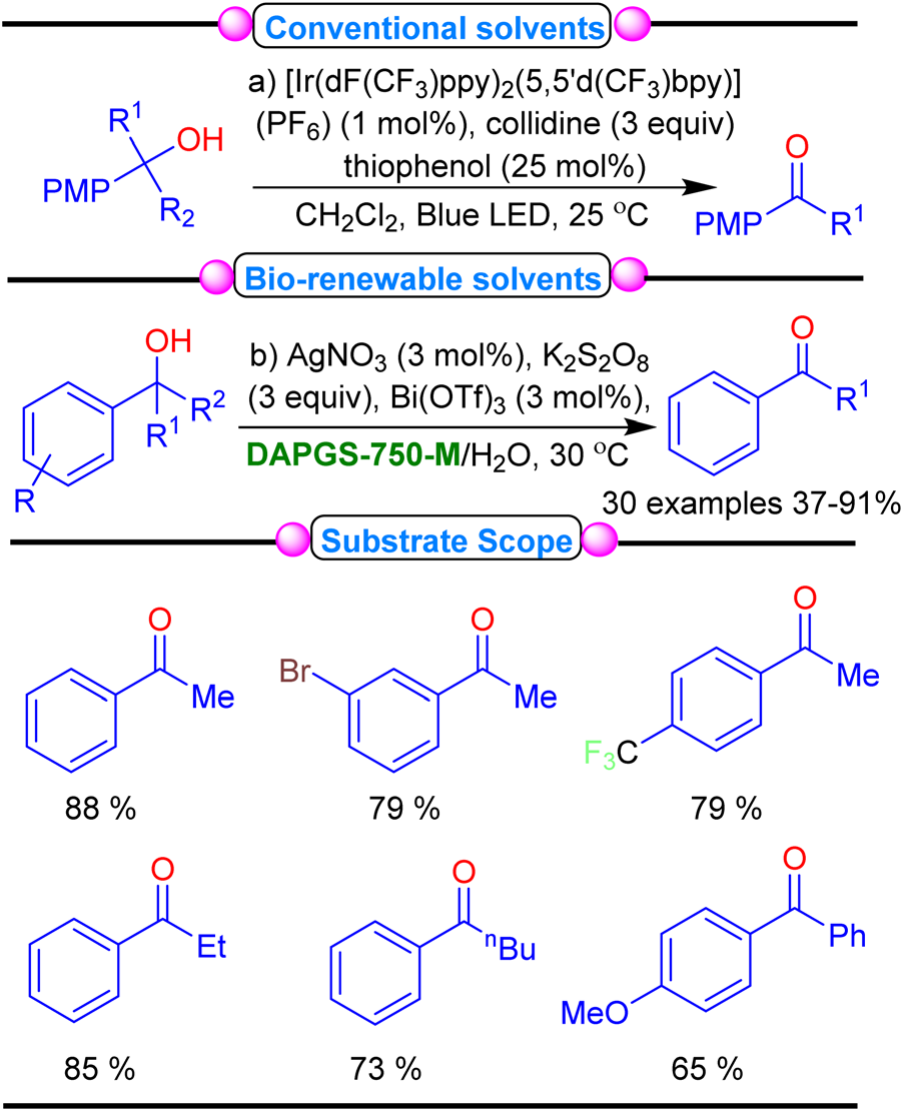
Oxidation of tertiary alcohols to ketones.

One of the most crucial oxidation reactions in organic chemistry is the transformation of alcohols into carbonyl compounds.^320–322^ A unique photocatalytic technique for converting tertiary alcohols to ketones in dichloromethane was recently published by Knowles (Scheme 89a).^323^ Chen *et al.* introduced a novel rosin-based surfactant DAPGS-750-M for the aqueous-phase oxidation of tertiary aryl carbinols into the corresponding ketones in good to excellent yields.^324^ Furthermore, it was claimed that this surfactant would work well in conjunction with other micellar catalysis surfactants, such as the well-known TPGS-750-M. They also proposed that β-scission of alkoxy radicals mediated the oxidation process. The use of an aqueous reaction medium in this oxidation technology is noteworthy, as it facilitates the recycling of the DAPGS surfactant, catalysts, and water within the flask. Both electron-donating and electron-withdrawing substituents were equally effective in facilitating the actions of aromatic tertiary alcohols (Scheme 89b). Furthermore, heterocyclic substrates were observed to undergo smooth oxidation under optimal conditions, and even asymmetric diaryl alcohols demonstrated competence. Remarkably, there was no evidence of C–methyl or C–phenyl bond breaking.

**Scheme 90:**
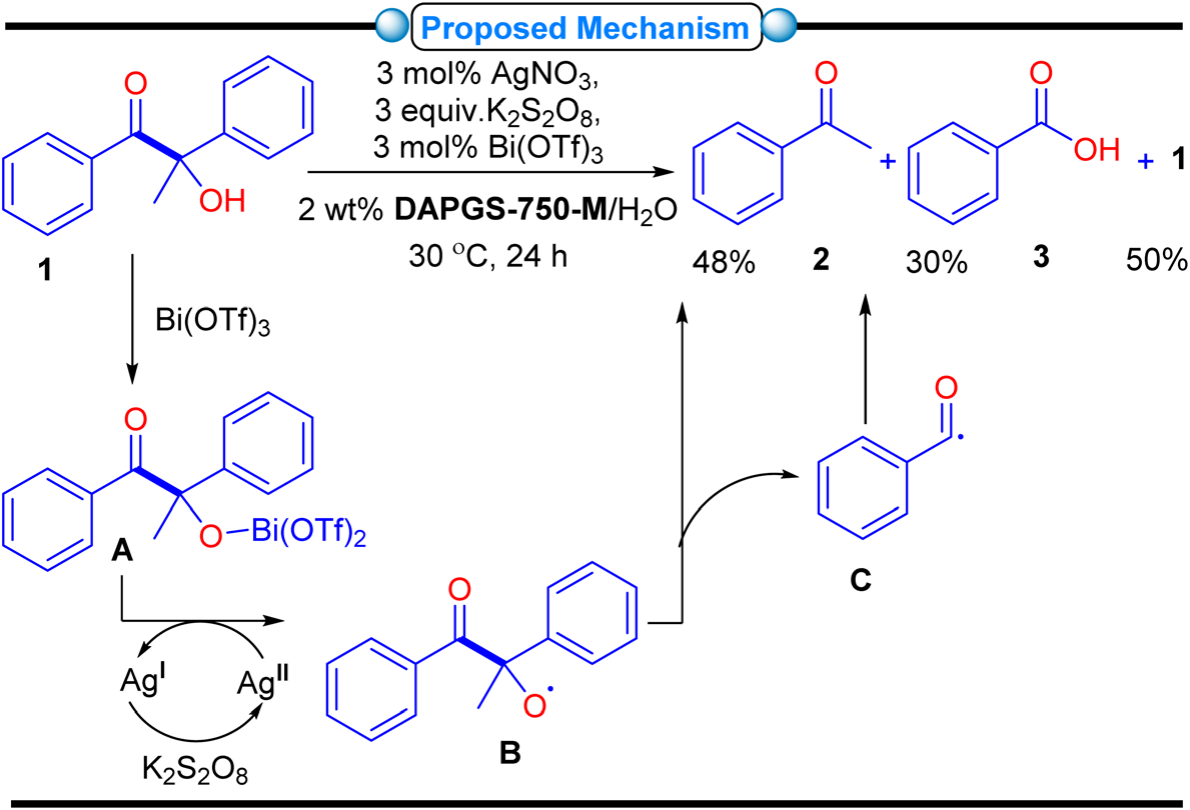
Proposed reaction mechanism of converting tertiary alcohols in ketone.

The reaction mechanism was supported by control experiments using TEMPO and early literature on the bismuth triflate/alcohol complex A formed in the process (Scheme 90). Alkoxyl radical B would then be produced by a simple Bi–O bond homolysis. After β-scission of the alkoxyl radical B, acetophenone 2 and carbonyl radical C were produced. The carbonyl radical C was then further oxidized to produce benzoic acid 3. The intended ketone product was extracted from the aqueous solution using minimal amount of EtOAc. Aryl carbinol and K_2_S_2_O_8_ were then added to the residual aqueous mixture, causing further oxidation. It was pointed out that alcohol complex A and bismuth triflate would have formed first. Alkoxyl radical B would then be produced by a simple Bi–O bond homolysis. After β-scission of the alkoxyl radical B, acetophenone 2 and carbonyl radical C were produced. The carbonyl radical C was then further oxidized to produce benzoic acid 3.

### 4.9. PTS (PEG600)

**Scheme 91:**
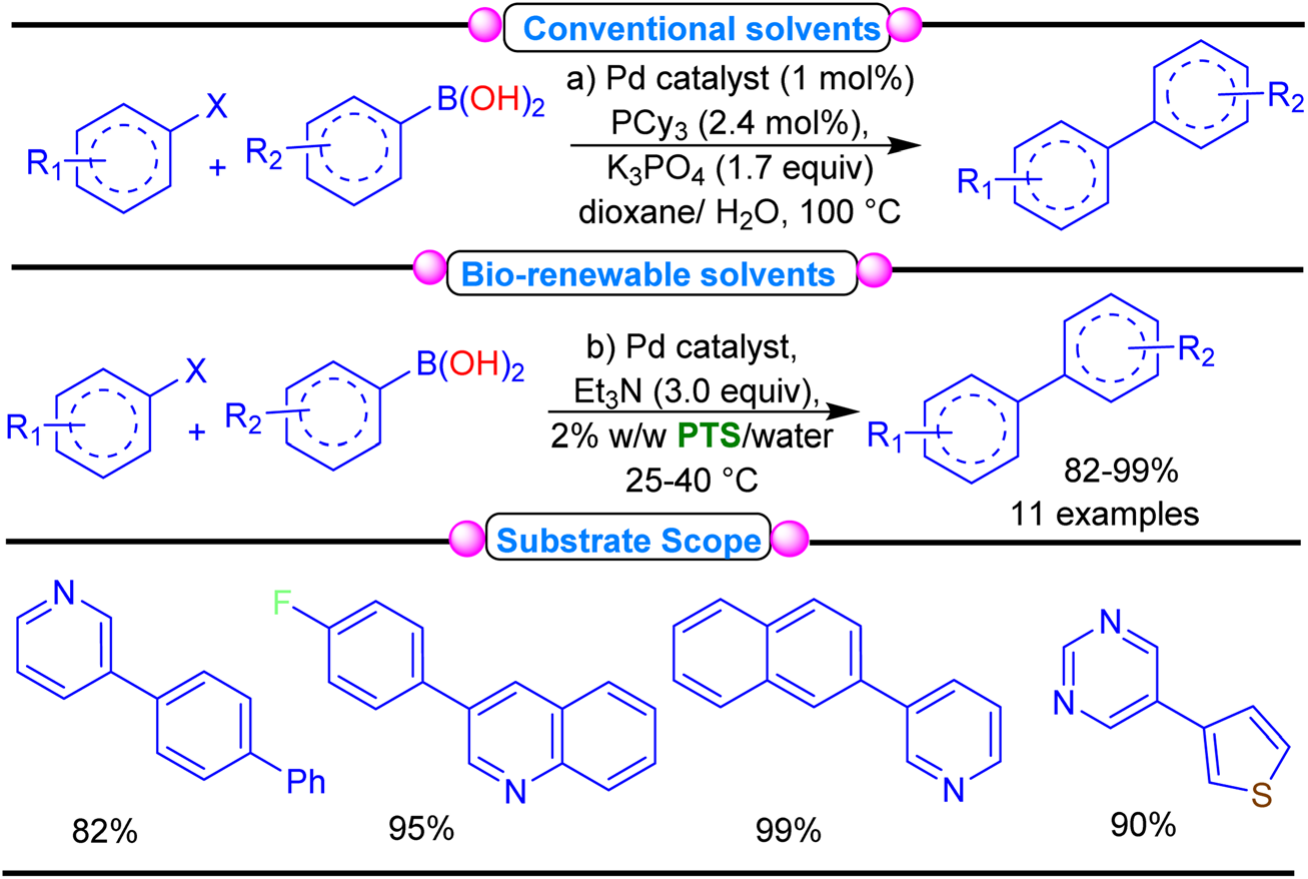
Sonogashira cross-couplings in PTS/water system.

Lipshutz *et al.* found that under mild conditions, a variety of water-insoluble heteroaromatic coupling partners were amenable to Suzuki–Miyaura cross couplings using PTS even at 40 °C (Scheme 91b).^325^ Recently, amphiphile PTS (polyoxyethanyl-*R*-tocopheryl sebacate), a commercially available nanomicelle-forming product, was introduced. At room temperature, both aryl and heteroaryl boronic acids were produced in satisfactory yields within a time period of 4–20 h. Similarly, biaryls were produced under similar conditions by aryl bromides, but a gentle heating to 40 °C was required because of the lower reactivity of these boronic acids. This method provides a significant, environmentally friendly alternative to eliminate harmful organic solvents from organic reactions, such as 1,4-dioxane (Scheme 91a).^326^

**Scheme 92:**
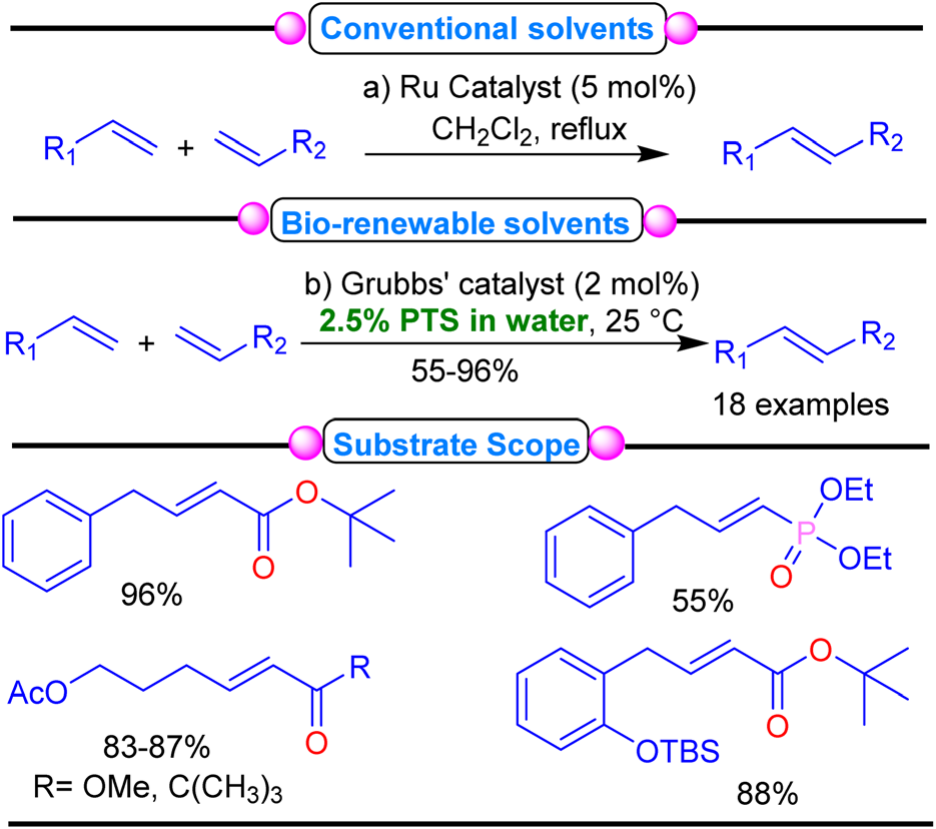
Olefin cross-metathesis reactions.

Lipshutz *et al.* discovered a non-ionic surfactant PTS, which allowed olefin metathesis in water at room temperature (Scheme 92b).^327^ The mixture of 2.5% PTS/water and 2% Grubbs-2 catalyst create a stable, rose-coloured colloidal dispersion. Although most CM reactions are carried out at temperatures above 40 °C, these reactions are carried out at room temperature, open to the air. *E*/*Z* ratios typically resemble those found in organic media and a wide range of functional groups, including allylic silanes, free alcohols, derivatives of amino acids, and epoxides, are tolerated. Combination of surfactant and water has been found to be a better catalyst than CH_2_Cl_2_ (Scheme 92a).^328^

**Scheme 93:**
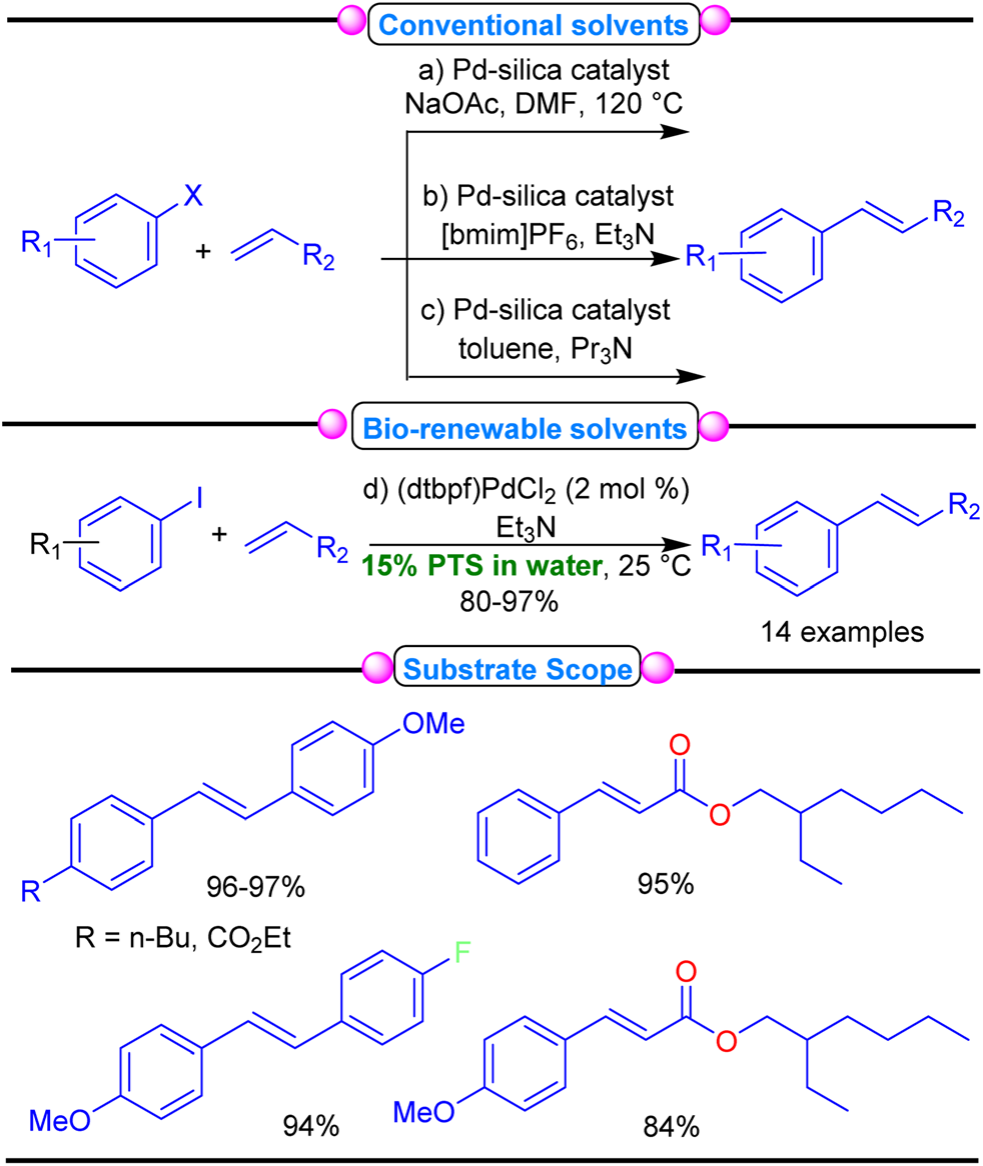
Heck couplings of aryl iodides with alkenes.

Literature about the transformation of unactivated alkenes has been reported by researchers.^329^ Lipshutz *et al.* designed a straightforward operational procedure for performing conventional Heck couplings in neutral water at room temperature. The use of low-cost PTS, a non-ionic amphiphile, enables cross-couplings in conditions that are particularly tolerant of environmental influences. Compared to less lipophilic methyl and benzyl ester analogues, commercially available *tert*-butyl and 2-ethylhexyl acrylate seemed to be more effective. To highlight PTS's water solubility, acrylates and aryl iodides were selected as substrates. Compared to acrylates, styrenes are more reactive coupling partners. Unsymmetrical (*E*)-stilbenes were smoothly afforded by both electron-rich and electron-poor aryl iodides. Substrates that resemble styrene, like water-insoluble vinyltriazole, can be easily combined with tetrahydroisoquinoline that has been highly functionalized. It was simple to carry out this reaction without the need of ionic liquid (Scheme 92b), cosolvents, or other hazardous solvents (Scheme 93a and c).^330–332^

**Scheme 94:**
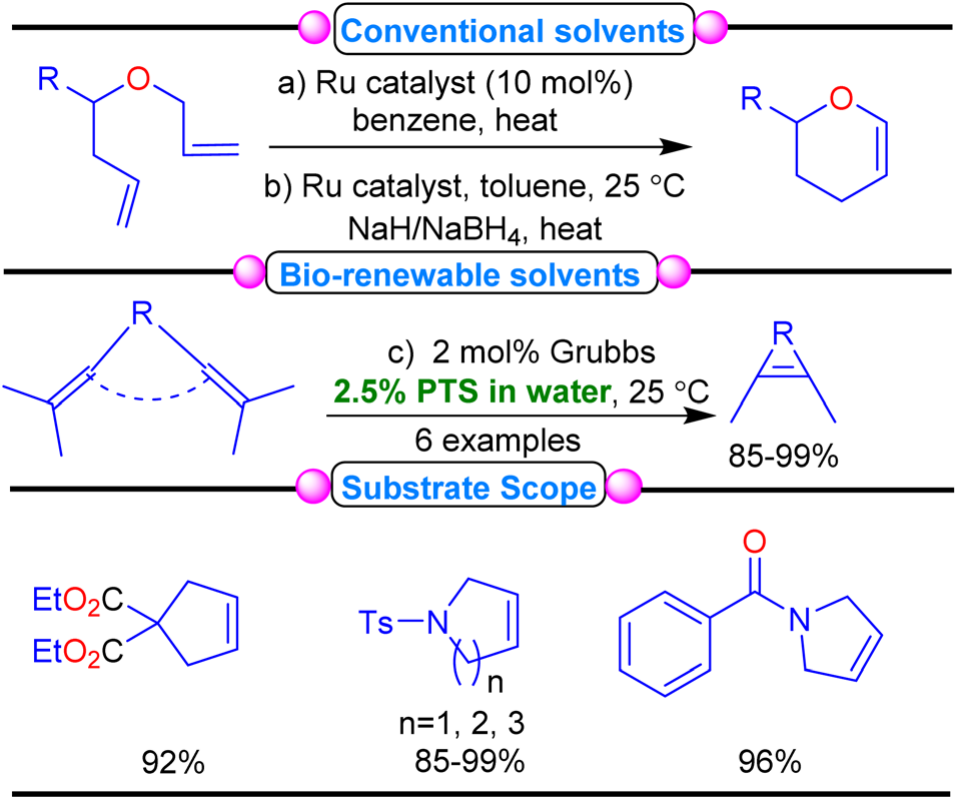
Ring-closing metathesis reactions.

Aguinaldo *et al.* have discovered an exceptionally straightforward protocol that enables room-temperature ring-closing metathesis reactions involving lipophilic substrates and a highly active, water-insoluble ruthenium catalyst in water as the sole medium (Scheme 94c).^333^ Ring sizes with five, six, and seven members are smoothly formed. It is possible to realize rings with and without heteroatoms. *N*-Arylsulfonyl or benzoyl substrates are examples of amine-precursors. It's interesting to note that the reaction rate and efficiency did not change when the total reaction concentration in water was raised from to 0.3 M, bringing the PTS content down to 0.8% (by weight). Unlike earlier research on RCM reactions, hazardous solvents like benzene (Scheme 94a) and toluene (Scheme 94b) were used.^334,335^

### 4.10. Carbonates

**Scheme 95:**
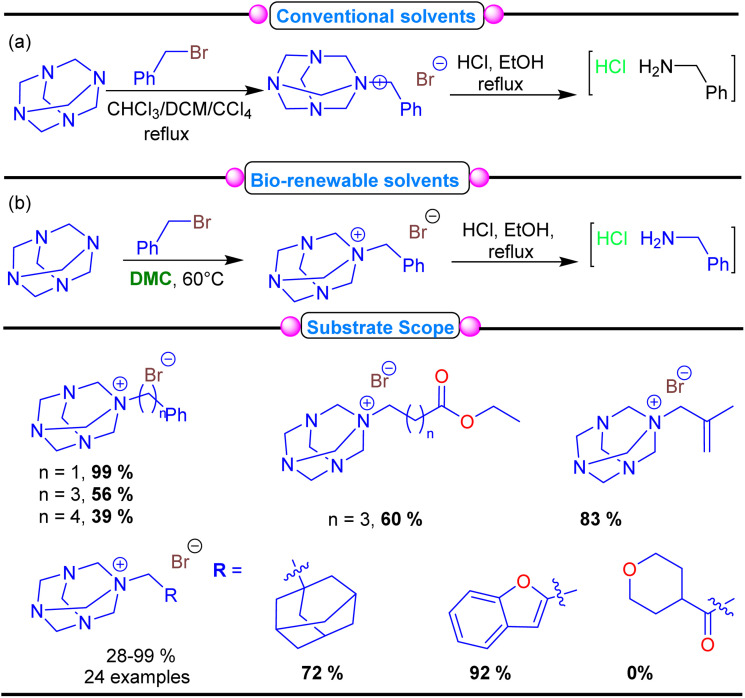
Delepine reaction using DMC as a reaction media.

Regarding the formation of primary amines from activated alkyl or aryl halides, Jordan *et al.*^336^ discussed their efforts to enhance the sustainability of the Delepine reaction (Scheme 95b). Hexamethylenetetramine (HMTA) was used for aminating alkyl or benzyl halide using DMC as a solvent for synthesising several pharmacologically significant building blocks. Delepine reaction is typically performed in CHCl_3_, DCM and CCl_4_ (Scheme 95a), which are concerning based on the GlaxoSmithKline (GSK) parameters.^337–339^ A number of substrates with a broad range of functional groups and moieties to the pharmaceutical industry were studied. Excellent yields were obtained for electrophilic/activated substrates. Alkene, alkyne, methylcyclopentyl, pyridyl, Boc-piperidine, and substrates containing adamantyl were well tolerated. Poor electrophiles, such as unactivated linear alkyl substrates, afforded lower yields.

**Scheme 96:**
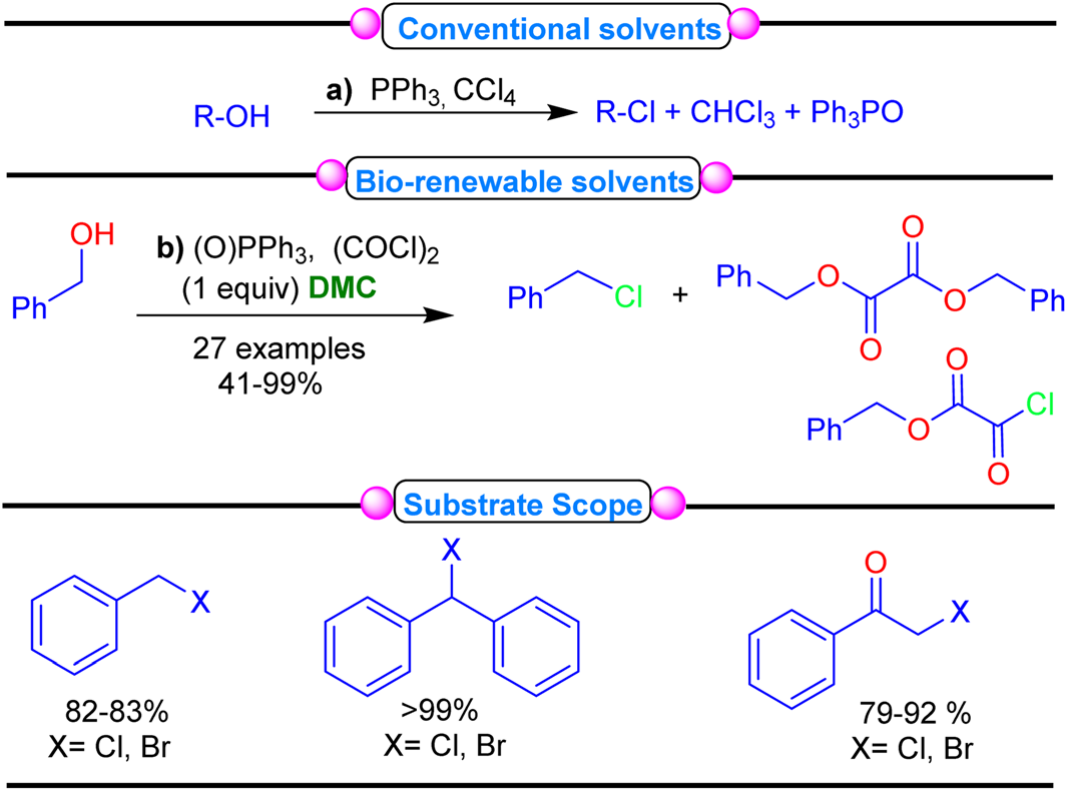
Triarylphosphine oxide-mediated Appel chlorination of benzyl alcohol.

Jordan *et al.* (2020) disclosed a chromatography-free Appel chlorination and bromination with a quantitative recovery of catalyst (Scheme 96b).^340^ Compared to the previous methods,^341–343^ sustainability was achieved by replacing chlorinated solvents with DMC as a green solvent and triphenylphosphine oxide (PPh_3_O) as a recyclable catalyst. After completion of reaction the catalyst were recovered by precipitating from the reaction mixture. The novel reaction conditions showed a general compatibility with alkyl, benzyl, allylic, and propargylic alcohols with near-quantitative yields. Only a 2% loss of catalyst were reported, showing promise for process scalability. A thorough analysis of the catalytic cycle revealed REACT-IR to be an effective technique for *in situ* reaction monitoring.

**Scheme 97:**
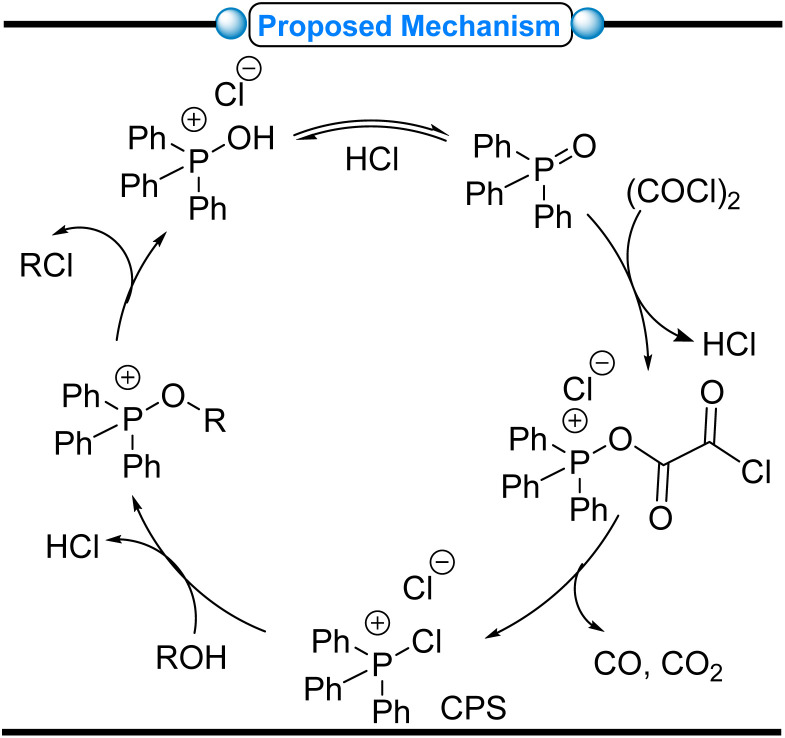
Catalytic scheme for the Ph_3_PO-mediated Appel reaction.

The reaction mechanism was proposed by the following consecutive steps (Scheme 97), initially PPh_3_O was reacted with oxalyl chloride in chloroform to produce its corresponding chlorophosphonium salt (CPS). In the subsequent step, CPS interacted with alcohol to produce alkyl chlorides in good to exceptional yields. The continuous regeneration of the catalytic species CPS through syringe-pumped injection of (COCl)_2_ into the reaction mixture completes the catalytic cycle.

**Scheme 98:**
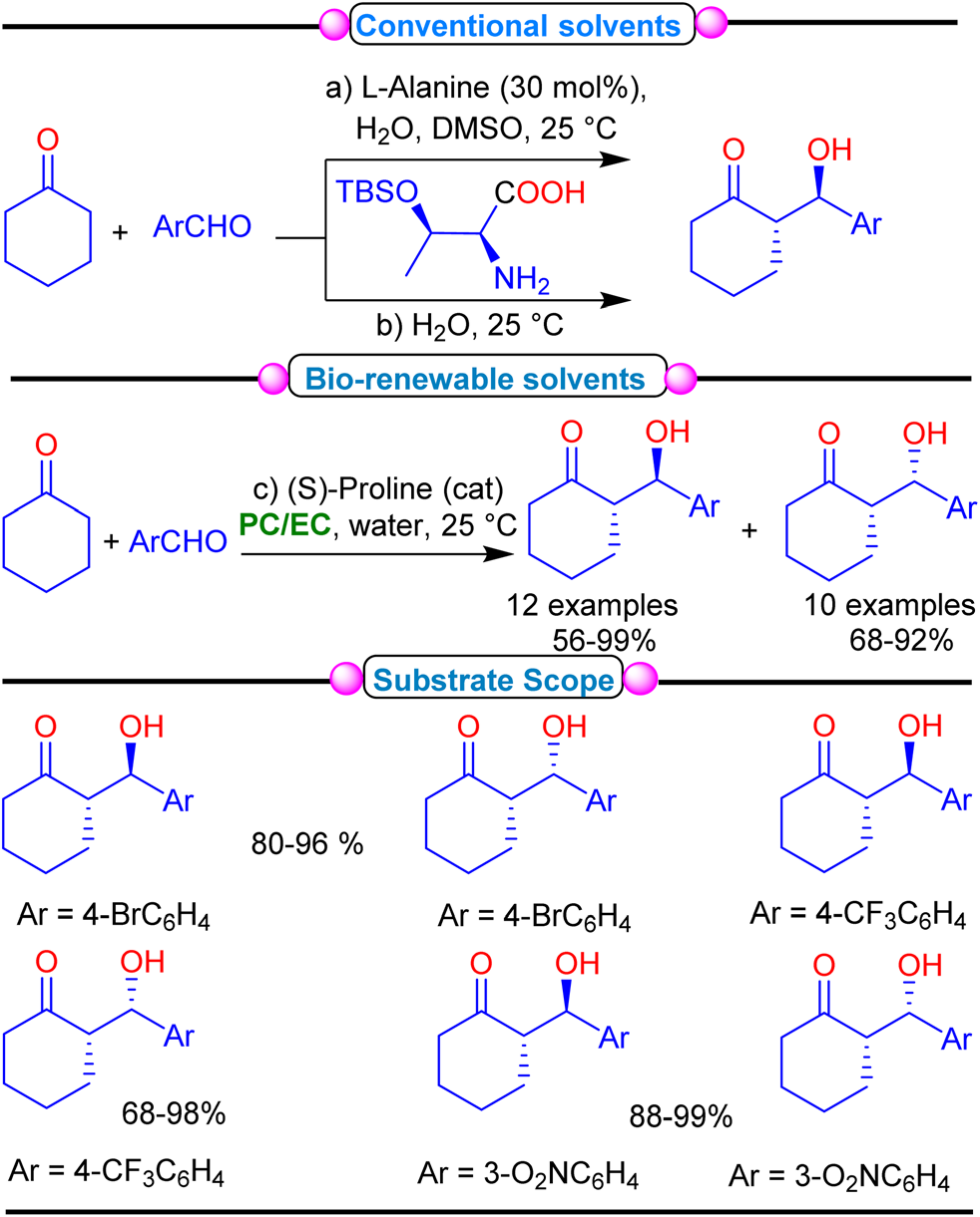
Amino acid-mediated asymmetric aldol condensation reactions.

Cyclic carbonates have been demonstrated by North *et al.* as an effective solvent for an aldol reaction catalysed by proline in an inert environment at room temperature (Scheme 98c).^344^ Cross-aldol reactions, which are organo-catalytic reactions, have also been conducted in some synthetic and water-based solvents (Scheme 98a and b).^345,346^ Nevertheless, there have been concerns raised over the environmental suitability of these solvents. This study focused on an aldol reaction between acetone and aromatic aldehydes to broaden the reaction's scope beyond cyclohexanone as the enamine precursor. Aldehydes bearing an electron-withdrawing group had a noticeably higher chemical yield in propylene carbonate; however, the yield in ethylene carbonate was even higher. Additionally, ethylene carbonate had superior diastereo- and enantioselectivity than propylene carbonate. The chemical yield of 3-nitrobenzaldehyde improved further when the solvent was changed from propylene carbonate to ethylene carbonate.

**Scheme 99:**
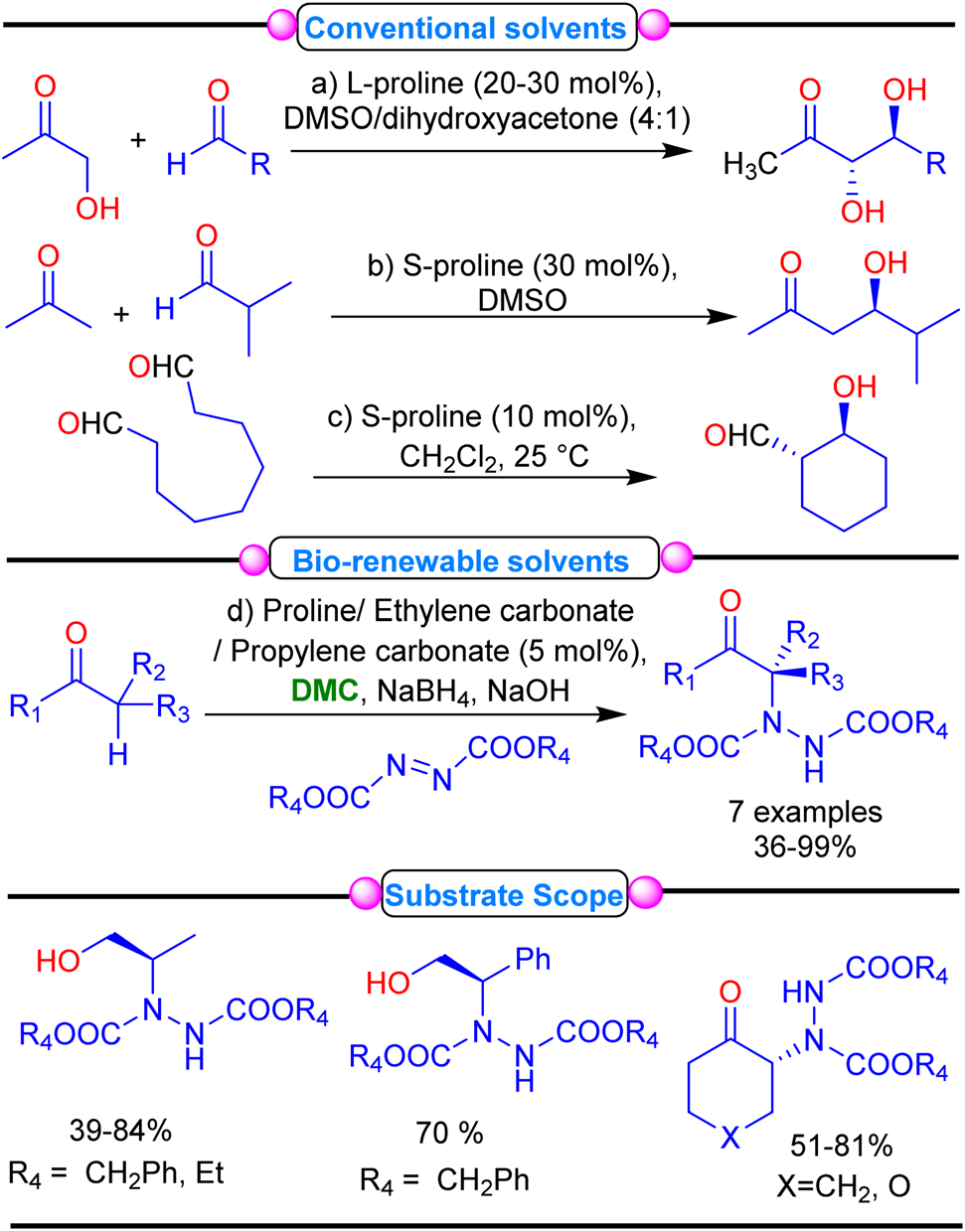
α-Hydrazination of aldehydes and ketones.

Beattie *et al.* in 2011 added proline to a stirred solution of diethyl azodicarboxylate and propanal in propylene carbonate for proline-catalysed α-hydrazination of aldehydes and ketones by diazodicarboxylates utilizing DMC (Scheme 99d).^347^ Nonanal produced alcohol with a considerable yield and good enantioselectivity. On the other hand, phenyl acetaldehyde produced an excellent yield of alcohol but with a low enantiomeric excess since the aldehyde racemized quickly. The preliminary investigations revealed that although the reaction with dibenzyl azodicarboxylate did happen under the typical conditions established for aldehyde substrates. In proline-catalysed α-hydrazinations of aldehydes and ketones, propylene carbonate is a sustainable and eco-friendly substitute compared to DMSO (Scheme 99a and b) and dichloromethane (Scheme 99c).^348–350^

**Scheme 100:**
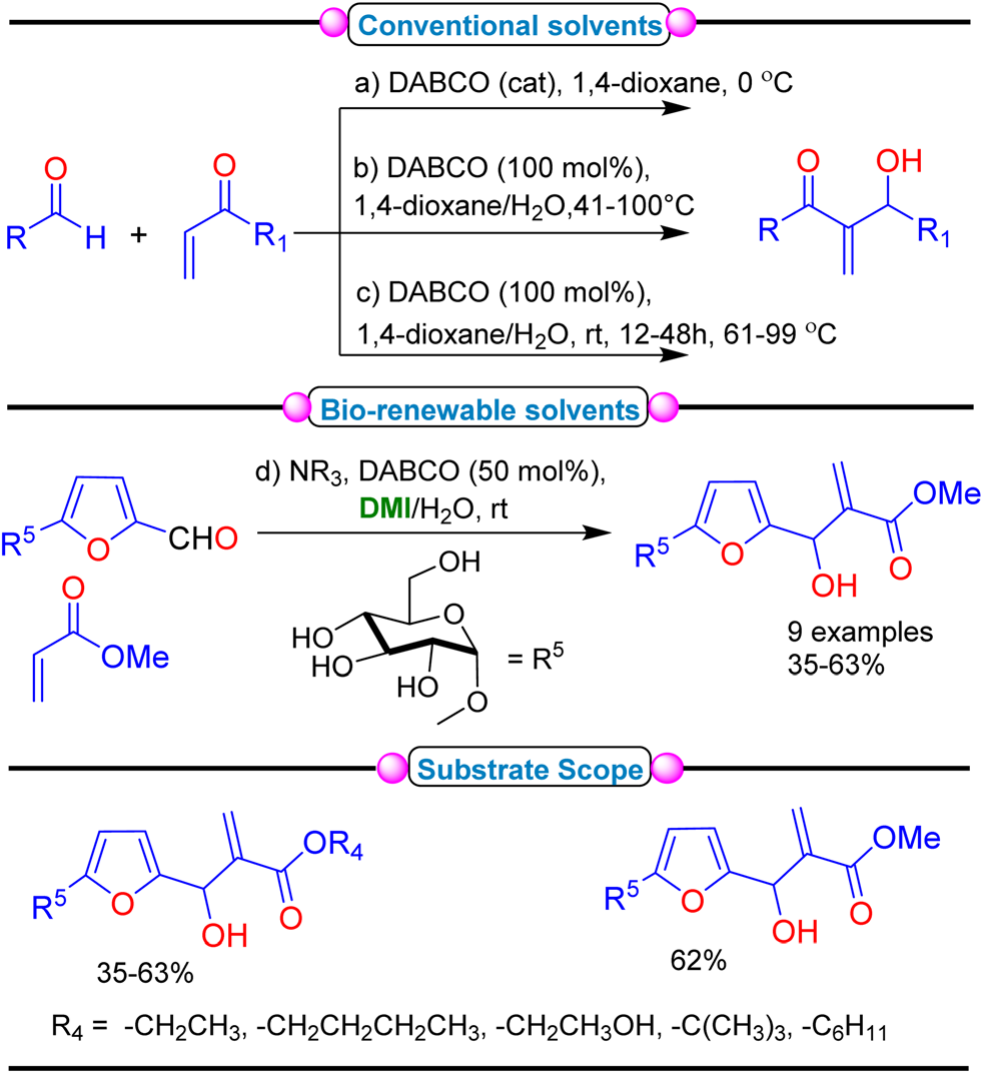
Baylis–Hillman reaction in bio-renewable solvents.

Tan *et al.* investigated the Baylis–Hillman reaction of α-GMF with acrylic building blocks in water (Scheme 100d).^351^ From the commercially available disaccharide isomaltulose, α-GMF is produced in a single-step approach. Compared to earlier techniques, it has been found that combinations of water and DMI, or pure DMI in some cases, can be used to replace 1,4-dioxane (Scheme 100a–c).^352–354^ The yields of the Baylis–Hillman adducts were comparable to those found for α-GMF. Since glycerol and lactic acid are potential precursors for bio-based acrylates.

**Scheme 101:**
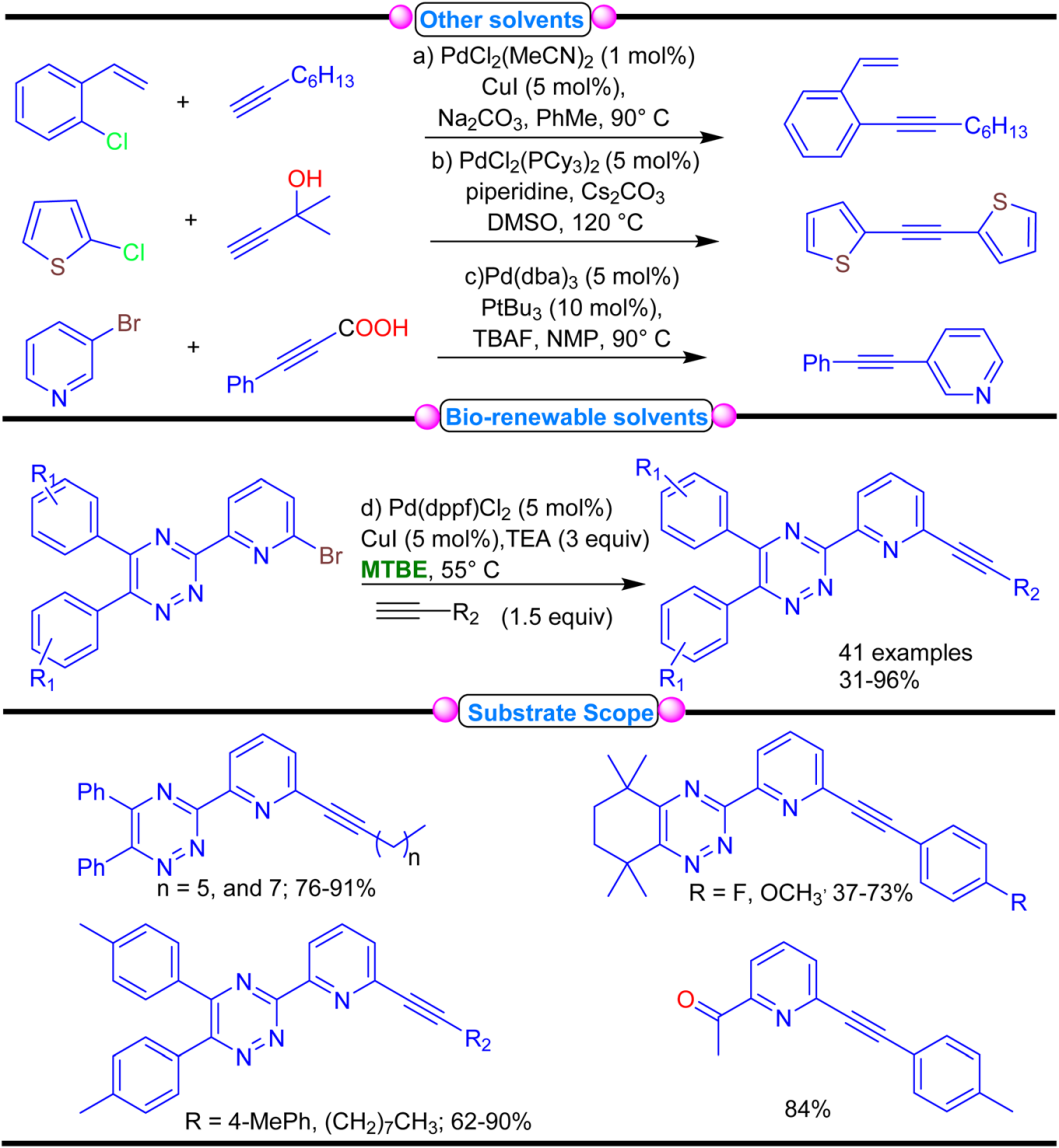
Palladium-mediated Sonogashira cross-coupling reaction.

A synthetic technique for obtaining Sonogashira coupling products from various functionalized 3-(6-bromopyridin-2-yl)-[1,2,4]triazine scaffolds was developed by Chaudhuri *et al.* (Scheme 101).^355^ These experiments also demonstrated the excellent selectivity of oxidative addition at the pyridinyl scaffold's C–Br bond over that of 1-ethynyl-4-bromobenzene. The intended products were produced in good yield from aliphatic alkynes, such as 1-octyne and 1-decyne. Satisfactory yields were also obtained using nonpolar complexant synthons, alternative aromatic scaffolds, and the substrates acetyl, formyl, cyano-, and methyl bromopyridines. There are several applications for the Sonogashira coupling reaction (Scheme 101a–c), where the hazardous solvents have been replaced with MTBE.^356–358^

**Scheme 102:**
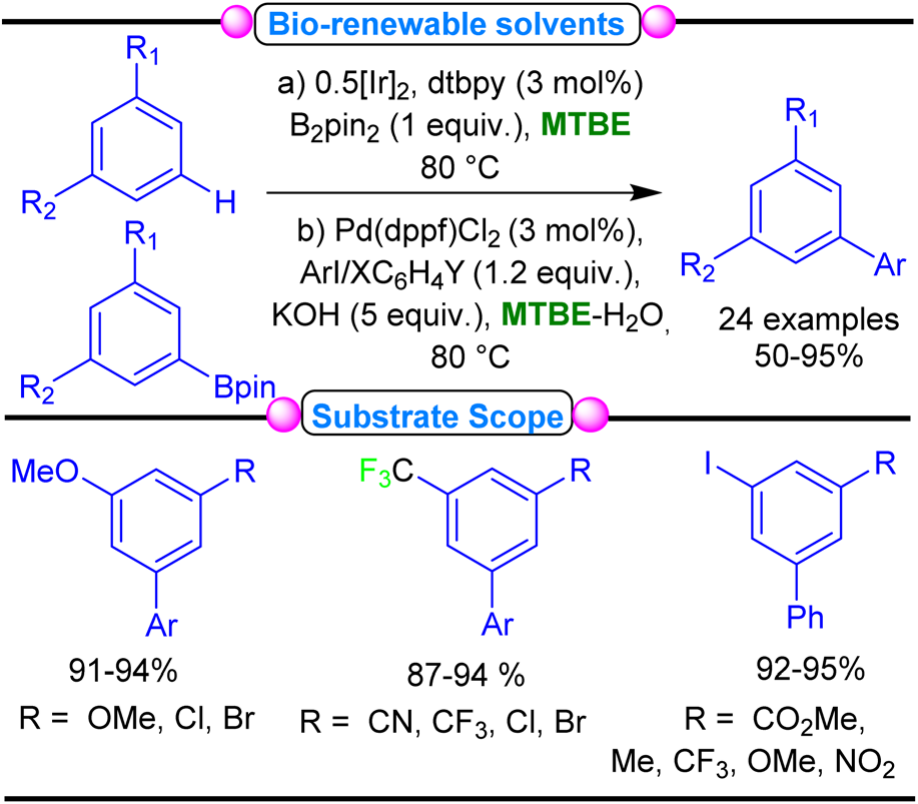
One-pot borylation of *m*-xylene followed by Suzuki–Miyaura coupling in MTBE solvent.

Harrisson *et al.* established a tandem reaction in 2009 to ascertain whether the Suzuki–Miyaura reaction could be undertaken in MTBE (Scheme 102b).^359^ The approach proceeds into two consecutive steps involves the C–H borylation and Suzuki–Miyaura cross coupling reaction. After *m*-xylene was fully borylated at the 5-position to yield its pinacolboronate ester, at a temperature of 80 °C. The method produced excellent yields for a variety of basic 1,3-disubstituted arenes containing both electron-donating and electron-withdrawing groups. Additionally, substituted heterocycles can also be used this tandem reaction sequence. Similarly, this process is not limited to the more reactive iodoarenes even though aryl bromides and chlorides can also function as the cross-coupling partner. Biphenyl are the most often employed systems that result in C–H activation, which had been replaced by MTBE in this instance to reduce waste and cost in tandem-reaction sequences (Scheme 102a).^360–362^

**Scheme 103:**
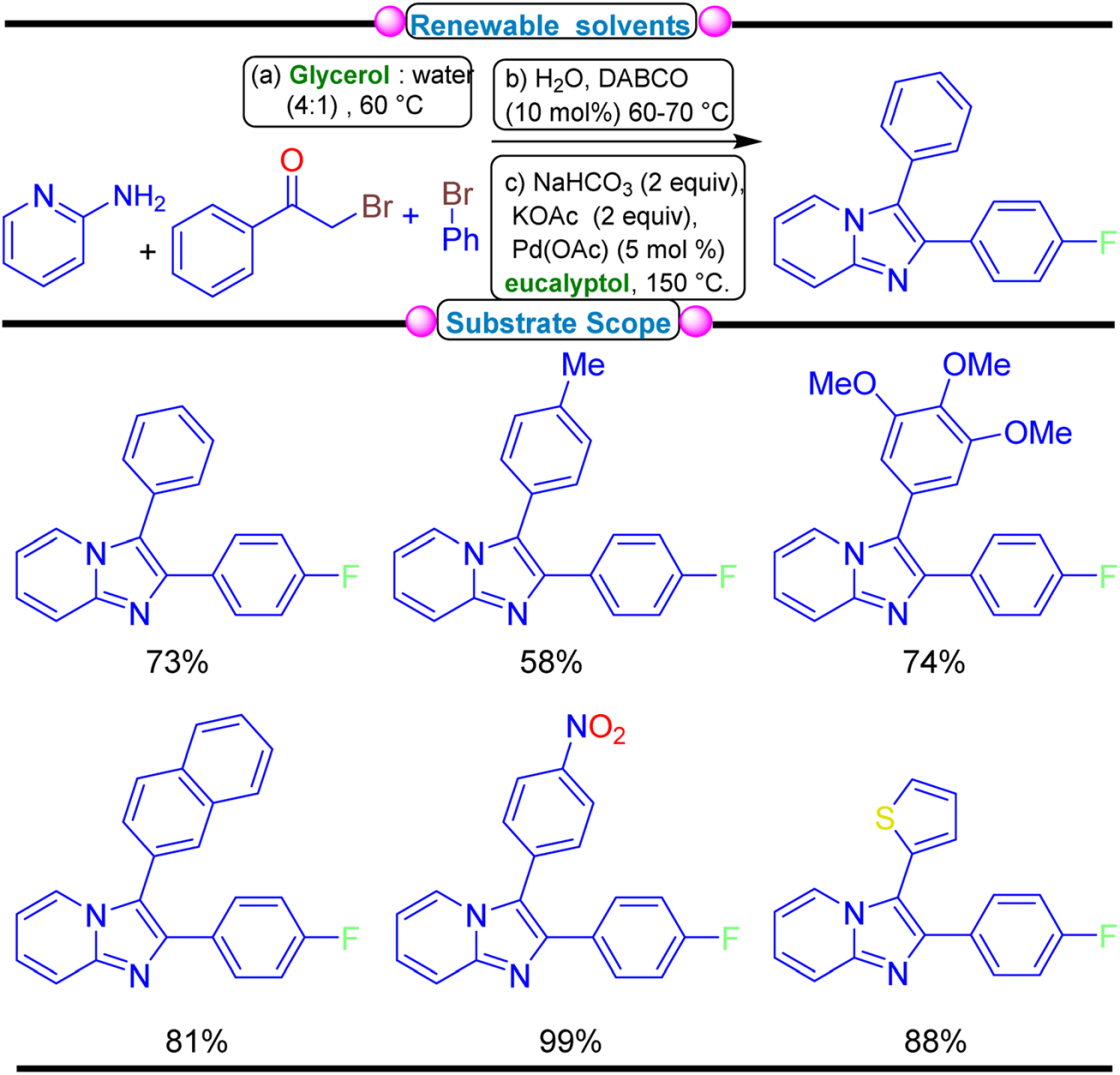
Synthesis of imidazo[1,2-*a*]pyridine in sustainable solvents.

Imidazo[1,2-*a*]pyridine is one of the most promising bicyclo[5.6] heterocyclic rings and is known as a “drug prejudice” scaffold.^363–365^ Numerous studies worked on the synthesis and structural alterations of this scaffold in an effort to find and create new therapeutic molecules employing glycerol (Scheme 103a) and water (Scheme 103b).^366–368^ In 2019, Campos *et al.* showed that bio-renewable eucalyptol could be utilised as an efficient reaction medium for diverse organic synthesis (Scheme 103c).^369^ In this devised protocol, starting materials containing oxygen, sulphur, and nitrogen in their chemical structure are employed for common palladium-catalysed cross-coupling reactions, such as the Suzuki–Miyaura and Sonogashira–Hagihara reactions. In place of common solvents such as anisole, bromobenzene, chlorobenzene, diethyl ether, diethyl benzoate, 1,4-dioxane, DMF, DMA, and DME. Eucalyptol was investigated to be a sustainable substitute. Eucalyptol is an attractive alternative to conventional solvents for the one-pot synthesis of 2,3-diarylimidazol[1,2-*a*]pyridines *via* a condensation between 2-aminopyridine and bromoacetophenones, followed by a C–H activation at C-3 of 2-aminopyridine. Operational simplicity was demonstrated by straightforward distillation of the solvent, which makes eucalyptol attractive from both economic and environmental perspectives.

**Scheme 104:**
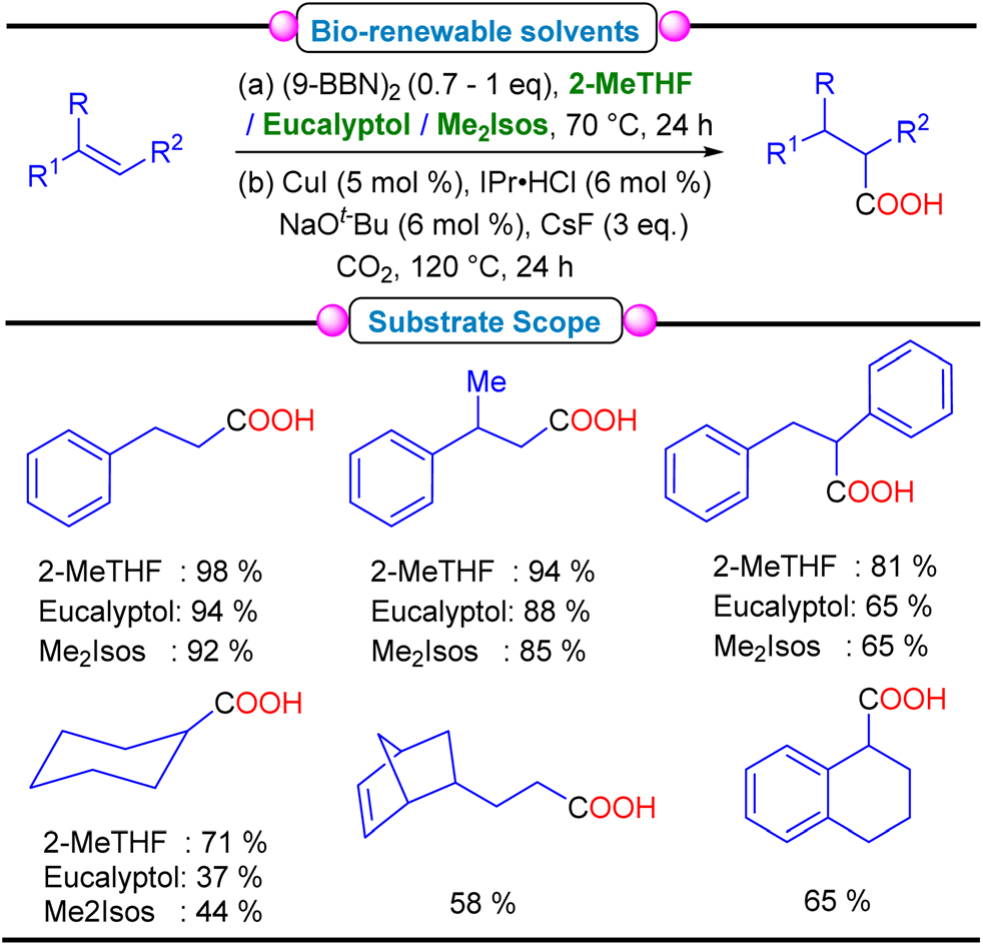
Cu-catalysed hydrocarboxylation of olefins in bio-derived solvents.

Several reactions catalysed by metals such as hydrocarboxylation, borocarboxylation, and copper-catalysed carboxylation of organoboronates and organoboranes are carried out in a bio-renewable solvent. The most effective biomass-derived solvents used in this carboxylation reaction include 2-MeTHF, DMI, eucalyptol and GVL. A. Gevorgyan in 2020 investigated carboxylation of alkenes from a sterically less hindered side, affording a moderate to excellent yields. Further carboxylated product was isolated in quantitative yield by using *in situ*-generated IPrCuI as the catalyst and CsF as the base (120 °C, 24 h) (Scheme 104).^370^ These carboxylation processes are not limited to benzylic alkenes only but also cyclohexenes and phenylacetylenes.

**Scheme 105:**
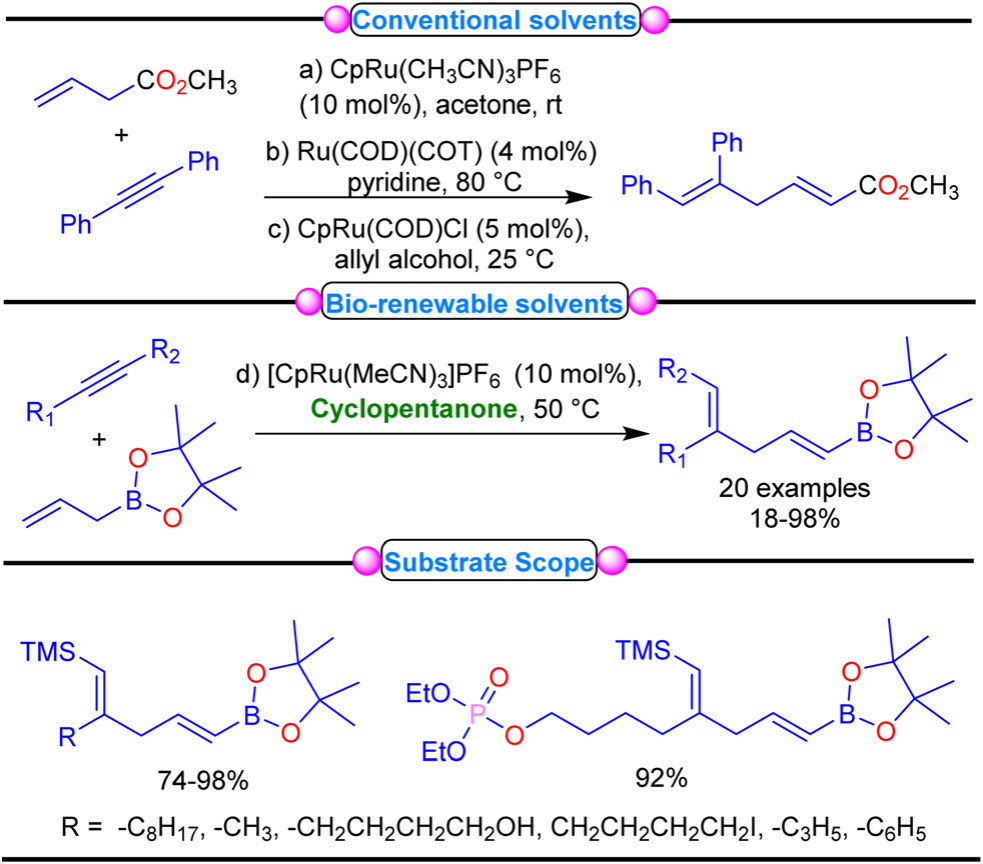
Ru-catalysed synthesis of vinyl boronates.

To produce synthetically useful vinyl boronates, Trost *et al.* devised a highly chemo-, regio-, and stereoselective Ru-catalysed alkene–alkyne coupling of allyl boronates and allyl silanes with different alkynes (Scheme 105d).^371^ When TMS-substituted alkynes were applied, a highly regioselective product was formed. Only the (*E*)-vinyl borane was produced in each of the cases under examination. While this conflict does not occur in the transition state for the branched product, the bulky TMS group sterically hinders the olefin, thereby disfavouring the transition state. In the reaction, branched and linear aliphatic substituents were tolerated. The scope encompasses both aromatic and aliphatic alkynes and provides access to key substrates that can be further modified with functional groups as needed *via* repetitive cross-coupling. Though an alkene–alkyne reaction catalysed by Ru–vinyl boronates had garnered a lot of interest, most of them utilised solvents like acetone (Scheme 105a), pyridine (Scheme 105b), and allyl alcohol (Scheme 105c).^372–374^

## 5. Synthesis of drug molecules using bio-renewable solvents

**Scheme 106:**
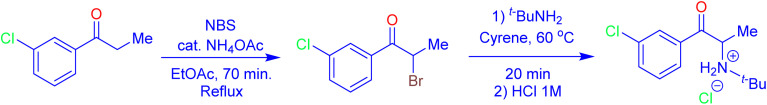
Green synthesis of the antidepressant drug bupropion.

Bupropion is an antidepressant that inhibits the reuptake of dopamine and noradrenaline, potentially giving pharmacological augmentation to more commonly used antidepressants.^375^ Sherwood *et al.* suggested a two-step green synthesis of bupropion, which was synthesised through a nucleophilic substitution process involving *tert*-butylamine and a secondary alkyl halide (Scheme 106). They proceeded by substituting the potentially carcinogenic DCM and reprotoxic NMP solvents with the green biobased solvent ethyl acetate and Cyrene, respectively, while molecular bromine was substituted with *N*-bromosuccinimide (NBS).^376^ The synthetic pathway began with 3-chloropropiophenone, in the presence of NBS with ethyl acetate as the solvent, which gave the corresponding secondary bromide. The resulting product underwent alkylation by *tert*-butylamine in Cyrene and then protonated to isolate bupropion as a hydrochloride salt.

**Scheme 107:**
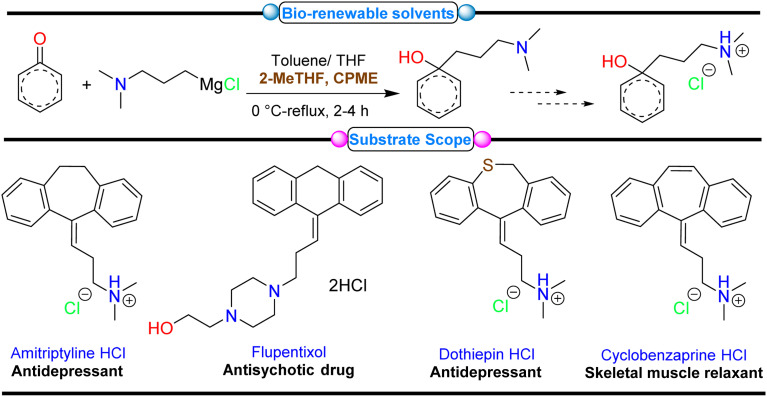
Synthesis of API intermediates by Grignard reaction using CPME as a bio-renewable solvent.

Kaki *et al.* highlighted the significance of green chemistry by validating the use of biomass-derived solvents, such as 2-MeTHF and CPME, during the synthesis of key active pharmaceutical intermediate (API) intermediates (Scheme 107).^377^ This work significantly contributed to the field of pharmaceutical chemistry by employing the efficient and scalable production of amitriptyline (antidepressant),^378^ flupentixol (an antipsychotic drug),^379^ dothiepin (antidepressant),^380^ and cyclobenzaprine (muscle relaxant),^381^ in desired purity and yield. Furthermore, replacing conventional toxic solvents reduced residual toxicity risks in finished products, thereby improving safety profiles.

**Scheme 108:**
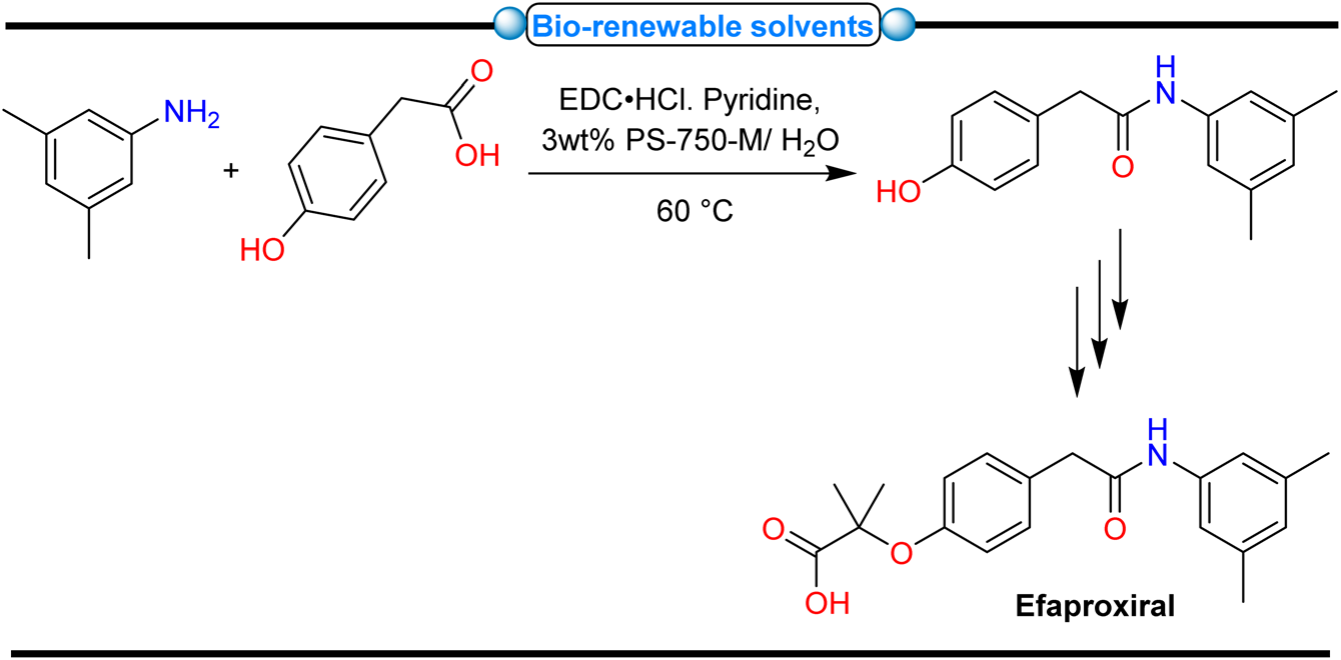
Amidation reaction as the key step during the synthesis of efaproxiral.

Handa *et al.* (2021) reported the synthesis of efaproxiral by using 1-ethyl-3-(3-(dimethylamino)propyl)carbodiimide (EDC) in a surfactant system (notably TPGS-750-M) (Scheme 108).^382^ Hydrophobic reactants were brought in molecular proximity within the micellar cores, thereby significantly accelerating the reaction rate. The key step in forming efaproxiral was the amide bond formation in water using nanomicelles. The method delivered moderate to excellent yields across a wide range of substrates with varying steric and electronic influences.

## 6. Industrial transition: scalability and economic viability

Despite the functional efficacy and sustainability advantages provided by the bio-renewable solvents in organic synthesis, their industrial integration is currently hindered by significant economic and technical barriers. The “green premium” remains a primary challenge, as the sophisticated biorefinery processes needed for these solvents cannot compete with the massive economies of scale of traditional solvents. Furthermore, the high boiling points of many bio-renewable solvents, though beneficial for reducing evaporative loss, often require energy-intensive purification strategies (*e.g.*, vacuum distillation). Functionally dense bio-renewable solvents lead to waste streams and poor process economics. Side reactions with strong bases or nucleophiles, leading us to re-optimise reaction conditions and kinetics. Future research should therefore focus on the development of inexpensive, high-volume, and functionally efficient bio-renewable solvents. The synthesis of these solvents from biomass feedstocks must also be made economically and environmentally appealing by coordinated research.

## 7. Conclusion

Research on bio-renewable solvents for organic transformations has grown significantly over the past two decades. Since solvents constitute an important class of industrial chemicals, replacing petroleum-derived solvents with biomass-derived alternatives can significantly enhance the sustainability of the organic chemical manufacturing industry and the economic viability of biorefineries. Although solvent-free methods, deep eutectic solvents, and ionic liquids are gaining increasing attention, conventional organic solvents remain indispensable, making renewable substitutes an attractive solution at least in the immediate future. Numerous types of organic transformations, including condensation, coupling, and multicomponent reaction, have been successfully carried out using bio-renewable solvents. The efficiency of bio-renewable solvents compared to conventional solvents is assessed based on reaction kinetics, selectivity towards a specific mechanistic pathway, and yield of the targeted product. Bio-renewable organic solvents can be classified by the functional groups present in their molecular structures, and their applicability to specific organic transformations can be determined. The functionalized bio-renewable solvent molecules, especially those with oxygen-containing functional groups, are produced from densely functionalized, highly oxygenated biomolecules with minimal redox interventions. GVL has received considerable attention as a bio-renewable solvent owing to its favorable physicochemical properties and ready availability from cellulosic biomass. Numerous studies demonstrate that the performance of bio-renewable solvents can match and even outperform their petroleum-derived counterparts. The preparation of bio-renewable solvents also offers advantages, such as utilization of abundant lignocellulosic and algal biomass, improved waste management, and reduced environmental impact. Despite their promise, the commercial adoption of bio-renewable solvents mandate comprehensive techno-economic assessments, life-cycle analyses, toxicity studies, and evaluations using green metrics. Currently, most bio-renewable solvents remain at the academic stage, although bio-based versions of established solvents such as THF and GBL are likely to achieve commercialization first. Future development should emphasize sustainable synthetic routes, solvent recovery strategies, and feedstock flexibility.

## Conflicts of interest

The authors declare that they have no known competing interests.

## Data Availability

No primary research results, software or code have been included, and no new data were generated or analysed as part of this review.
